# Supramolecular Self-Assembly of Engineered Polyproline Helices

**DOI:** 10.1021/acsmacrolett.3c00304

**Published:** 2023-06-26

**Authors:** Dominic F. Brightwell, Giada Truccolo, Kushal Samanta, Helena J. Shepherd, Aniello Palma

**Affiliations:** † School of Chemistry and Forensic Science, Supramolecular and Interfacial Chemistry, Ingram Building, The University of Kent, Canterbury CT2 7NZ, Kent, United Kingdom; ‡ School of Chemistry, University College Dublin, Belfield, Dublin 4, Ireland

## Abstract

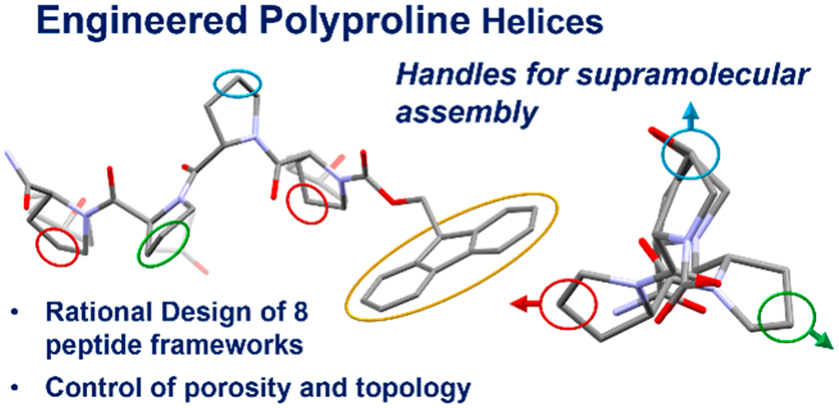

The ability to rationally design biomaterials to form desired supramolecular constructs presents an ever-growing research field, with many burgeoning works within recent years providing exciting results; however, there exists a broad expanse of promising avenues of research yet to be investigated. As such we have set out to make use of the polyproline helix as a rigid, tunable, and chiral ligand for the rational design and synthesis of supramolecular constructs. In this investigation, we show how an oligoproline tetramer can be specifically designed and functionalized, allowing predictable tuning of supramolecular interactions, to engineer the formation of supramolecular peptide frameworks with varying properties and, consequently, laying the groundwork for further studies utilizing the polyproline helix, with the ability to design desired supramolecular structures containing these peptide building blocks, having tunable structural features and functionalities.

The synthesis of hierarchical supramolecular functional materials is an exciting field of research with applications in biomedicine,^1,2^ separation and catalysis,^3−5^ and sensing.^6^ Pivotal to the successful design of these supramolecular constructs is the ability to synthesize building blocks with specific topologies. Pioneering work in the field of extended and discrete metal organic frameworks was instrumental in demonstrating the importance not only of the nature of the chemical handles but also of their relative position in space.^7,8^ While a high level of positional control of these handles can be achieved with relative ease on classical (poly)aromatic building blocks, the same cannot be stated for structured peptides as supramolecular building blocks. Recently, biomolecules such as peptides, lipids, and DNA/RNA have appeared in a number of reports as interesting building blocks in the synthesis of novel 2D and 3D biomaterials which assemble using supramolecular interactions.^6,9−13^ We are particularly interested in the use of structured peptides as supramolecular building blocks. Peptides can be prepared at scale with high purity, have canonical and noncanonical amino acids incorporated into their primary structure with high accuracy, and are biocompatible.^14^ This results in a vast array of accessible structures, creating an expanse of chemical space yet to be explored with a broad chemical diversity of potential building blocks and secondary structures. As such, the efforts to investigate this class of compounds as chiral, tuneable ligands has seen a surge in recent years.^4,5,15−19^ Peptides are typically flexible and chiral and present a multitude of chemical side chains, which can result in complex inter- and intramolecular interactions. Consequently, it can be challenging to obtain good quality single crystals for solid state analysis.^6^ Moreover, when structured peptides such as α-helices and β-sheets are used as supramolecular building blocks, it is known that they can suffer perturbation of their periodicity upon functionalization, which leads to the inhibition of predictable self-assembly. The nature of the amino acids, their position within the sequence, and side-chain-to-side-chain interactions are all aspects that need to be carefully considered to minimize the risk of perturbation of the secondary structures in order to achieve a predictable periodicity.^20^ With these challenges in hand, it is essential to have a thorough understanding of the peptide secondary structure, and the resulting interacting moieties, to predict the assembly of the peptide ligands within supramolecular constructs. It is within this context that we propose the use of polyproline helices as supramolecular building blocks. The polyproline II helix is both rigid and stable in short sequences and has three repeating helical faces, creating predictable and accessible handles for functionalization and supramolecular assembly.^21−23^

We have recently demonstrated that super short polyproline helices (tetrameric peptides) can assemble into a reversibly porous supramolecular peptide framework (SPF) capable of engaging in stereoselective host encapsulation.^24^ Herein, we demonstrate the ability to utilize functionalized short polyproline helices as predictable ligands with an exceptional level of control, for the rational design of a series of H-bonding-driven supramolecular peptide frameworks. The design principles successfully applied to these peptides can be used to drive the design of more complex materials; the periodicity of the helix allows the expansion of the principles of assembly found in these minimalistic peptides to longer peptide chains, while the resilience of the helix to functionalization means these principles can be applied to various functional groups.

Minimalistic peptides have the potential to play a key role in the emerging field of bionanomaterials.^25−27^ We recently reported the first SPF formed by the self-assembly of a polyproline helical tetramer, Fmoc-(Pro)_4_-NH_2_, **P**_**4**_. Despite the short length of this tetrapeptide, **P**_**4**_ crystallized in the polyproline II helical form, a common secondary structure found in nature.^28^ The formation of this porous framework was driven by hydrogen-bond donor and acceptor (H_D_–H_A_) interactions between the hydrogens of the primary amide at the C-terminus and the carbonyl groups on the peptide backbone, as well as Fmoc–Fmoc association predominantly driven by dispersion interactions.^24^ These results gave us insight into the potential of short polyproline helices as supramolecular building blocks.

We aim to exploit the full potential of polyprolines as minimalistic peptides in the construction of emerging bionanomaterials. We are particularly interested in the functionalization of position 4 (or γ-carbon) of the proline amino acid as this position is exposed on the exterior of the helix^22^ and is capable, upon functionalization,^29^ of engaging in the formation of supramolecular interactions. Remarkably, analyzing the **P**_**4**_ supramolecular framework, we were able to successfully predict the effect of hydrogen donor (i.e., **H**_**D**_; −OH) interactions on the supramolecular assembly for a series of polyproline peptides. Led by design principles based off the **P**_**4**_ framework, a series of seven hydroxy-functionalized derivatives were synthesized using Fmoc-based solid-phase peptide (SPPS) techniques (Figure 1, SI 1). We anticipated that if the polyproline II conformation was retained for these peptides the spatial orientation of the hydroxyl group of a hydroxyproline residue would be highly predictable, thereby enabling future endeavors in the rational design of polyproline-based ligands to assemble into supramolecular bioconstructs. If successful, we would demonstrate the resilience and high level of positional control achievable using proline-based minimalistic peptides. Therefore, with this information it would be feasible to rationally design polyproline-based peptides with predictable geometries of functional motifs for the incorporation of further supramolecular interactions, to be utilized in supramolecular assembly. The repeating nature of the polyproline helix also means that, from the detailed investigation of functionalization of a minimalistic tetrameric oligoproline (four residues constituting one full turn of the polyproline II helix), it is possible to infer the structural details of longer peptide units with multiple helical turns and apply these findings as a strong foundation to design more complex polyproline-based peptides.

**Figure 1:**
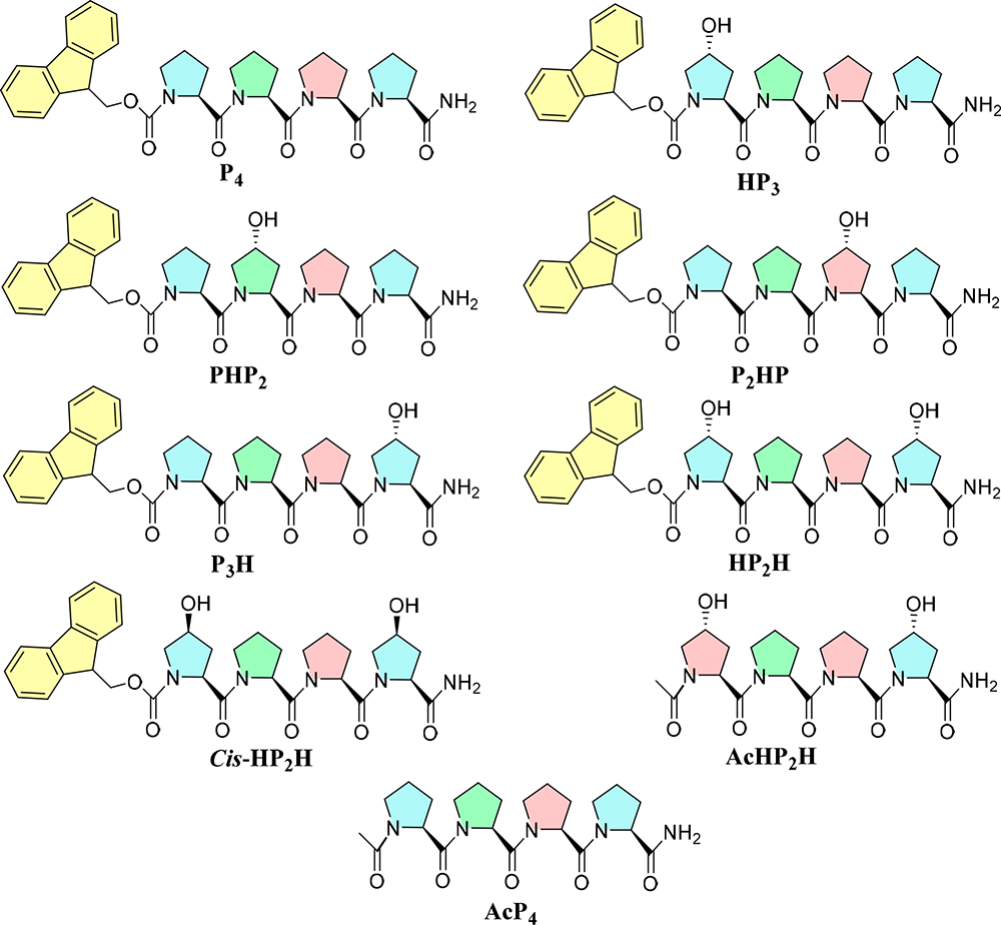
Chemical structures of synthesized oligoproline peptides. Helical faces are highlighted in different colors (i+3 periodicity). H in the peptide name indicates the position of the hydroxyproline starting from the N-terminus in the sequence.

Analyzing the crystal structure of **P**_**4**_, we were able to observe that the terminal prolines, Pro1 (N-terminus) and Pro4 (C-terminus), clearly have close contact with the neighboring peptide’s carbonyl groups along the *b*-axis (Figure 2a).^24^ We anticipated that a H-donor group such as the hydroxyl group introduced in position 4 of the first proline in the sequence would engage in intermolecular hydrogen-bonding interactions along the *b*-axis without significantly impacting the packing topology.

**Figure 2:**
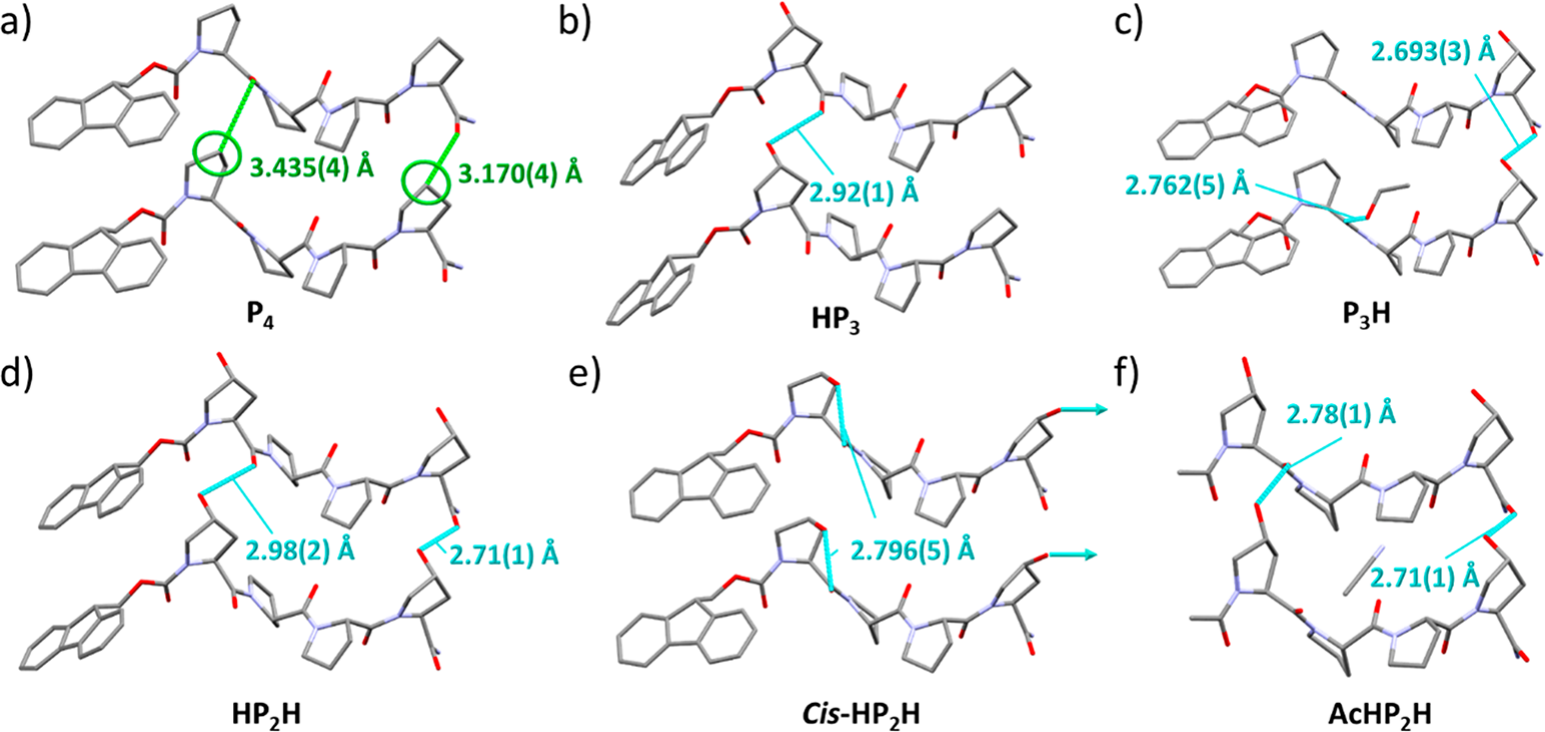
Crystal structures of peptides **P**_**4**_ (a), highlighting close contacts between Cγ (Pro_1_ and Pro_4_) and the adjacent peptide’s closest carbonyl groups, **HP**_**3**_ (b), **P**_**3**_**H** (c), **HP**_**2**_**H** (d), **Cis-HP**_**2**_**H** (e), and **AcHP**_**2**_**H** (f), showing the new hydrogen bond interactions formed by the additional hydroxyl moieties. The **cis-HP**_**2**_**H** C-terminus hydroxyl has an undefined hydrogen bond to disordered solvent within the channels of the framework.

Thus, it can be inferred that the crystal structures formed should adopt, through functionalization of the terminal residues, the same hydrogen bonds and Fmoc–Fmoc interactions as in the **P**_**4**_ structure, with additional H-bonding (O-H—O=C) along the same plane as the amide (N-H—O=C) H-bonding interactions, to form similar 2D sheets; this packing of the peptide units is highlighted in a simplified model in Figure 3b. These sheets should stack in various assemblies to form either porous, as in the **P**_**4**_ framework, or nonporous structures. With these details in mind, a series of peptides were synthesized by replacing the terminal prolines with hydroxyprolines, **HP**_**3**_, **P**_**3**_**H**, **HP**_**2**_**H**, **cis-HP**_**2**_**H**, **AcHP**_**2**_**H**, and **AcP**_**4**_ (Figure 1), varying not only the number of hydroxyl groups but also the N-terminal capping moiety (SI 3). The first peptide tested **HP**_**3**_, Fmoc-Hyp-Pro_3_-NH_2_ (Figure 1), crystallized to form a nonporous structure, driven by the addition of a hydrogen bond between the Pro1 hydroxyl to the neighboring peptide’s Pro1 carbonyl, forming 1D hydrogen-bonded tapes of the peptide along the *b*-axis (Figure 2b), similar to the tapes of peptide along the *b*-axis already present in **P**_**4**_ (Figure 2a). The amidated C-terminus formed the same hydrogen-bond interactions (-NH_2_—O=C) as found for the **P**_**4**_ framework, with interactions with the Pro2 and Pro3 carbonyls of adjacent antiparallel peptides. The assembly of the peptides in these hydrogen-bonded layers into 2D layers via Fmoc–Fmoc interactions is very similar to the crystal structure of **P**_**4**_. However, these 2D sheets then stack with a slight displacement to form the 3D framework, significantly changing the unit cell and reducing the void volume, resulting in no solvent-accessible channels present within the framework (Figure 4a).

**Figure 3:**
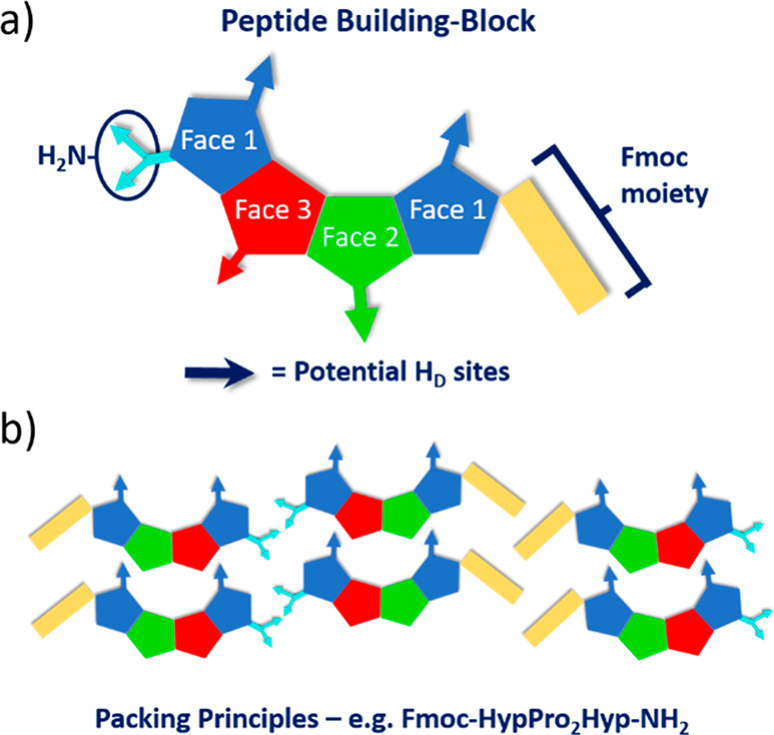
(a) Model of a general peptide building block with sites of intermolecular interaction shown. (b) General packing principles of hydrogen-bonded layers. Example: peptide **HP_2_H** shown.

**Figure 4:**
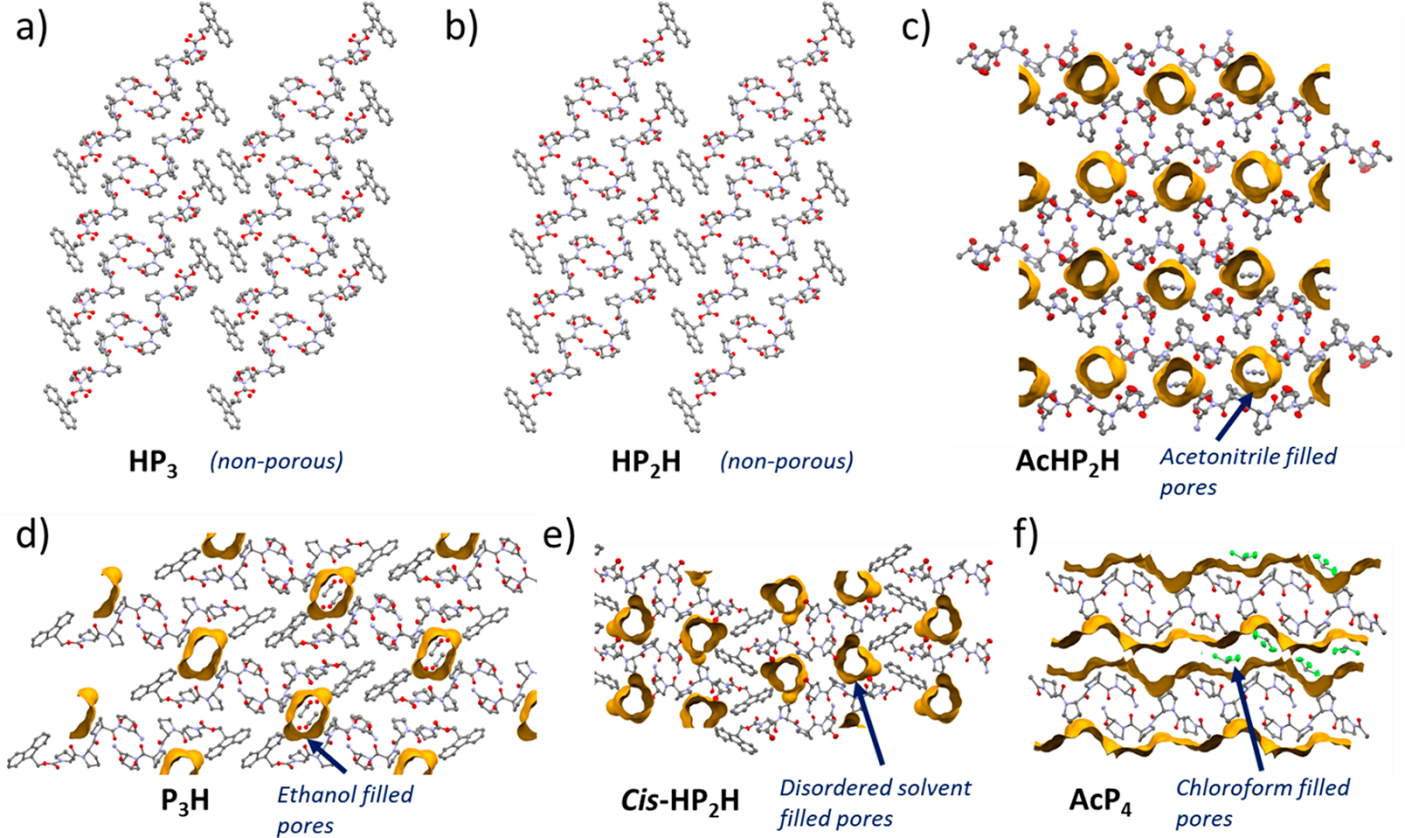
Crystal structures of peptides **HP**_**3**_ (a), **HP**_**2**_**H** (b), **AcHP**_**2**_**H** (c), **P**_**3**_**H** (d), **cis-HP**_**2**_**H** (e), and **AcP**_**4**_ (e), showing their extended structures viewed along the rows of peptides. The channels are highlighted in yellow; solvent-filled pores are partially shown; and atomic displacement parameters are shown at 50% probability.

The peptide **P**_**3**_**H**, Fmoc-Pro_3_-Hyp-NH_2_, crystallized as a porous structure with channels (volume 342.6 Å^3^, 10%/unit cell, probe *r* = 1.2 Å, grid spacing 0.4 Å; SI 3.1.5) isostructural to the **P**_**4**_ framework (Table 1) except for a doubling of the *c*-axis, to be expected from the doubling of *Z*′. These pores are a slightly larger size than those found in the original framework (volume 245.09 Å^3^, 13.8%/unit cell, probe *r* = 1.2 Å, grid spacing 0.4 Å; Figure 4d), which presents a good example of the accessibility of alternative structures via editing of the peptide monomers, allowing tuning of the pore environment for various functionalities. In this case, the asymmetric unit was comprised of two N-terminally hydrogen-bonded peptides. The new hydroxyl group has a hydrogen bond interaction with the Pro4 carbonyl of the adjacent parallel peptide, occurring similarly for both peptides within the asymmetric unit (OH—O=C, 2.693(3) and 2.762(5) Å, Figure 2c). The precise placement of the −OH groups along the same helical face, i.e., Pro1 and Pro4, produces H-bonding interactions toward neighboring carbonyls of Pro1 and Pro4 units, respectively, thus highlighting the exceptional control possible, producing interactions with specific predictable geometries.

**Table 1:** 

Structures	Porous (Y/N)	Crystal System	Space Group
**P**_**4**_^a^	Y	Monoclinic	*P*2_1_
**P**_**2**_**HP**^a^	Y		
**P**_**2**_**HP-P**_**4**_^a^	Y		
**P**_**3**_**H**^a^	Y		
**HP**_**3**_^b^	N	Monoclinic	*C*2
**HP**_**2**_**H**^b^	N		
**cis-HP_2_H**	Y	Orthorhombic	*P*2_1_2_1_2_1_
**AcHP_2_H**	Y	Orthorhombic	*P*2_1_2_1_2_1_
**AcP_4_**	Y	Monoclinic	*P*2_1_

a Isostructural to each other.

b Isostructural to each other.

As both **HP**_**3**_ and **P**_**3**_**H** peptides form similar hydrogen-bonded layers within their frameworks (Figure 2b,c), we predicted functionalization of both positions would allow for both hydrogen bonds to be present simultaneously within the framework without significant disruption of the packing, as such a peptide **HP**_**2**_**H**, Fmoc-Hyp-Pro_2_-Hyp-NH_2_, was synthesized. Single crystals of **HP**_**2**_**H** were successfully obtained in the same manner as **HP**_**3**_/**P**_**3**_**H**, and they were subsequently used for single-crystal analysis. The crystal structure contained both hydrogen bonds present in the previous structures as predicted, with similar hydrogen bond distances (Pro1 OH—O = C; 2.98(2) Å vs **HP**_**3**_; 2.92(1) Å. Pro4; 2.71(1) Å vs **P**_**3**_**H**; 2.693(3) Å, Figure 2d). However, the extended structure was isostructural with peptide **HP**_**3**_ (Table 1) with only small differences in the unit cell parameters (SI 3.1.7). The successful formation of this framework and retention of the polyproline II helix, despite 50% functionalization of the peptide, clearly highlight the resilience of the polyproline helix and how the ability to predict the geometry of new functionalities can be utilized to rationally design supramolecular constructs.

To observe the impact of cis-hydroxy, versus the trans-hydroxy previously used, on the packing topology, the peptide **cis-HP**_**2**_**H**, Fmoc-cis-Hyp-Pro_2_-cis-Hyp-NH_2_, was synthesized, with both hydroxyprolines cis rather than trans. In this case, we expected the 4*S*-hydroxyproline to prefer the endo conformation and internal hydrogen bond to the hydroxyproline’s amide carbonyl, thus restricting the formation of intermolecular interactions.^30^ The crystal structure obtained contains channels filled with disordered solvent (volume 628.9 Å^3^, 171%/unit cell, probe *r* = 1.2 Å, grid spacing 0.4 Å, Figure 4e, SI 3.1.8) and was not isostructural to any of the other peptide frameworks (Table 1). As expected, the N-terminal hydroxyl adopted endo ring puckering, forming an intramolecular hydrogen bond (OH—O=C, 2.793(4) Å, Figure 2e) toward the same hydroxyproline’s amide carbonyl, not engaging in the supramolecular assembly.

With the addition of two hydrogen bonding interactions in the **HP**_**2**_**H** structure, it seemed apparent that these hydrogen bonding interactions should still produce an extended 2D structure without the Fmoc–Fmoc interactions. As such, the peptide **AcHP**_**2**_**H**, Ac-Hyp-Pro_2_-Hyp-NH_2_, was synthesized, whereby the Fmoc protecting group on the N-terminus was replaced with an acetyl group (Figure 1). Sonication of a saturated solution in acetonitrile yielded an organogel (SI 3). Heating and slowly cooling a supersaturated solution of the peptide in acetonitrile, or slow evaporation of an acetonitrile solution, produced clusters of long fiber/needle-like crystals of the peptide, unlike those produced in any of the Fmoc-containing structures. These largely one-dimensional crystals were expectedly poorly diffracting; however, a crystal structure was obtained from a suitable crystal (SI 3.1.9), with the data giving a predicted powder pattern that matched well with powder X-ray diffraction (PDXRD) analysis of the bulk material (SI 3.2.9). Remarkably, the polyproline helix form II was retained for this peptide; moreover, as anticipated, analysis of the single-crystal data showed the retention of the same hydrogen bonding interactions present in the **HP**_**2**_**H** structure (Figure 2d,f), but with the loss of the Fmoc interactions, there are no other significant interactions extending along two of the axes (Figure 4c) while still forming channels within the framework (SI 3.1.9). Remarkably, comparing the structures of **AcHP**_**2**_**H** and **HP**_**2**_**H**, we can clearly conclude that, while not necessary to the formation of the peptide framework nor to the stability of the polyproline helix in such short peptides, the terminal groups can also be functionalized with chemical handles (e.g., Fmoc/acetyl) to increase the level of control of the supramolecular assembly process for these short peptides.

Due to the successful crystallization of the peptide **AcHP**_**2**_**H**, without the contribution of the Fmoc moiety, the peptide **AcP**_**4**_ (Figure 1), Ac-Pro_4_-NH_2_, was synthesized to demonstrate the contribution of the two terminal hydroxyprolines, versus the C-terminal amide −NH_2_, to the self-assembly of the peptide unit. Crystalline needles of **AcP**_**4**_ were obtained to be suitable for single-crystal X-ray diffraction (SCXRD) analysis. This data (SI 3.1.10) showed the formation of a similar assembly to **AcHP**_**2**_**H** (Figure 4f) but adopted a significantly different unit cell. The structure has large channels (volume 572.37 Å^3^, 36.1%/unit cell, probe *r* = 1.2 Å, grid spacing 0.4 Å) containing two CHCl_3_ molecules per peptide, coordinating to the Pro1 and Pro4 carbonyl groups (Cl3CH—O=C, 2.993(6) and 3.026(7) Å, SI 3.1.10Figure S47). This clearly demonstrates the ability of the peptide to crystallize without the presence of sterically bulky, rigid groups (e.g., Fmoc), with a minimally functionalized tetramer forming a porous framework. This example finally proves that an unfunctionalized proline tetramer can adopt the polyproline II helix.^31−33^

Peptide **PHP**_**2**_, Fmoc-Pro-Hyp-Pro_2_-NH_2_, did not crystallize effectively. We hypothesize that the absence of favorably positioned **H**_**A**_ groups close to the Pro2 C4 position (closest carbonyl **H**_**A**_, C—O 4.447(4) Å) in the **P**_**4**_ framework is impacting the assembly process. However, PDXRD of the precipitate, from the attempted crystallization of **PHP**_**2**_ in EtOAc/EtOH, showed partial crystallinity (SI 3.2.3, Figure S50).

The Pro3 residues of the **P**_**4**_ peptide face the channels of the framework and as such are prime candidates for functionalization to affect host–guest interactions (Figure 5a), without disruption of any supramolecular interactions forming the extended framework. Therefore, functionalization at this position should have a minimal impact on the adopted structure, giving rise to the same crystalline framework. As such, peptide **P**_**2**_**HP**, Fmoc-Pro_2_-Hyp-Pro-NH_2_, was synthesized and successfully crystallized with the same packing as the **P4** framework. The hydroxyl groups line the previous channels of the framework, with the main structural difference being the hydroxyl forcing *exo* puckering of the attached pyrrolidine ring, typical for this functionality^34^ and the subsequent reduction in pore volume (volume 161.8 Å^3^, 9.2%/unit cell, probe *r* = 1.2 Å, grid spacing 0.4 Å, Figure 5e, SI 3.1.4). This change in properties of the framework was exemplified via thermal activation studies, carried out on the **P**_**4**_ framework previously, whereby encapsulated solvent could be removed, resulting in the collapse of the channels forming a nonporous structure.^24^ In this case, heating the **P**_**2**_**HP** framework under reduced pressure at 45 °C did not show the formation of a second crystalline phase (Figure S51, SI 3.2.4), showing how the solvent-filled voids are now trapped in place by the additional hydroxyl moieties. This ability to functionalize the pores of the framework with no disruption of the peptide helix, thereby allowing tuning of the selectivity of the pores, has clear applications toward specific host–guest interactions.

**Figure 5:**
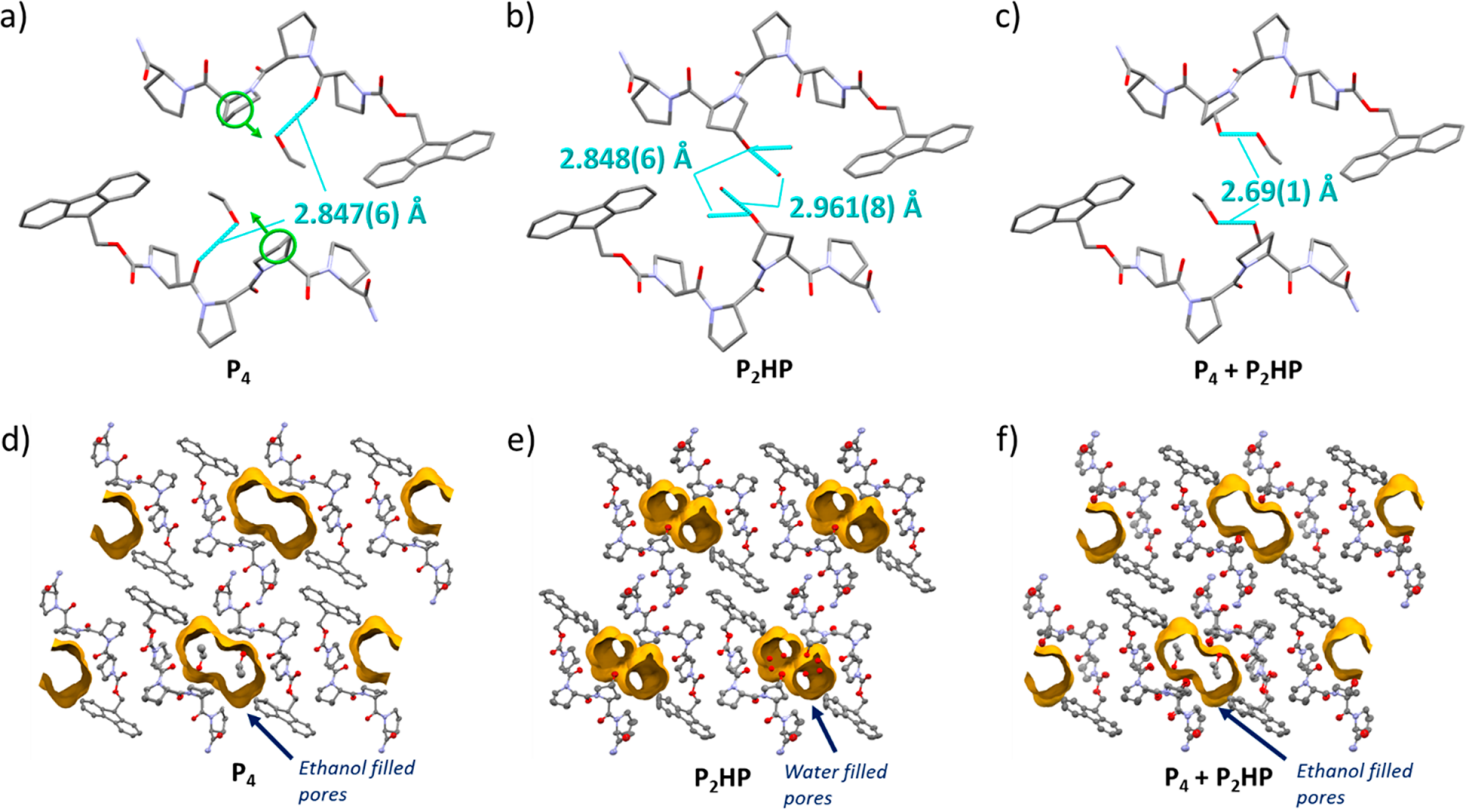
Crystal structures of **P**_**4**_ (a), **P**_**2**_**HP** (b), and **P**_**4**_**+P**_**2**_**HP** (c) mixed crystal, depicting two peptide units adjacent to the framework channels, viewed along the *b*-axis, mercury. Hydrogen bond interactions are shown. (c) is slightly off the *b*-axis to show both hydrogen bonds to different ethanol molecules. Crystal structures of **P**_**4**_ (d), **P**_**2**_**HP** (e), and **P**_**4**_**+P**_**2**_**HP** (f) mixed crystal, showing their closely matched extended structures viewed along the *b*-axis. The channels are highlighted in yellow, and one solvent-filled pore is shown for each. Atomic displacement parameters are shown at 50% probability.

As **P**_**4**_ and **P**_**2**_**HP** are isostructural, we theorized that both peptides would crystallize together, to yield either a cocrystalline material or a solid solution.^35^ If successful, then the two could be combined to create a porous framework spiked with the hydroxyproline moiety. When combined, the two peptides crystallized via cooling a supersaturated solution of the peptides (1:1 molar ratio) in ethanol. SC-XRD analysis of the crystals formed clearly showed the presence of both peptides (Figure 5f, SI 3.1.6). Within the pores of this SPF, the hydroxyl moiety had a reduced chemical occupancy of the oxygen atom (0.375), indicating the lack of differentiation between the two peptides during the self-assembly process, resulting in the formation of a solid solution,^35^ with **P**_**2**_**HP** randomly dispersed throughout the extended **P**_**4**_ framework. This result is not trivial, as it paves the way to the synthesis of discrete or extended supramolecular structures using different polyproline building blocks simultaneously and potentially synergistically. Upon thermal activation, the mixed framework **P**_**4**_**+P**_**2**_**HP** showed hybrid behavior compared to the two single peptide frameworks, with the powder pattern upon activation for **P**_**4**_**+P**_**2**_**HP** bearing similarities to both activated single peptide frameworks (i.e., **P**_**4**_ and **P**_**2**_**HP**) with some formation of the new characteristic peaks seen in the **P**_**4**_ “activated” diffractogram (Figures S53–56, SI 3.2.6), while a portion of the original peaks remained. This suggested only partial “activation” and collapse of the pores, thus showing that the additional hydroxy moiety alters the pore properties, restricting the collapse of the channels.

In conclusion, we have synthesized a series of hydroxyproline-based derivatives of an oligoproline tetramer, successfully forming novel H-bonding driven supramolecular peptide frameworks. Using rational design, based on the original supramolecular framework, for the placement of additional functional groups, we were able to consistently predict the position of the hydroxyl groups in relation to the polyproline II helix and therefore anticipate their effect on the topology of the H-bonding supramolecular network formed. This approach is not trivial, and our results are remarkable as we use a peptide-based supramolecular building block. Moreover, engineering polyproline peptides, through side chain functionalization and peptide capping, we can modulate the nature of the frameworks (i.e., porous or nonporous) as well as the size and the properties of the pores (e.g., solid solution framework) while functionalizing up to 50% of the peptide’s backbone. The resilience of the polyproline II helix is crucial in our methodology as it allows us to use their predictable geometries for the rational design of discrete and extended supramolecular three-dimensional structures. This work lays the groundwork for further studies focusing on the polyproline helix to rationally design structural units capable of forming desired supramolecular structures with tunable structural features and functionalities.

## Data Availability

CCDC-2127750,^24^ 2238152, 2238155, 2238160, 2238161, 2238180, 2238252, 2234312, and 2264145 contain the supplementary crystallographic data for this paper, including structure factors and refinement instructions, and can be obtained free of charge from The Cambridge Crystallographic Data Centre, 12 Union Road, Cambridge CB2 1EZ, UK (e-mail: deposit@ccdc.cam.ac.uk), or via https://www.ccdc.cam.ac.uk/getstructures.

## Supporting Information Available

Experimental methods and analytical data (PDF)
CIF file for **AcHP_2_H** (CIF)
CIF file for **AcP_4_** (CIF)
CIF file for ***cis*-HP_2_H** (CIF)
CIF file for **HP_2_H** (CIF)
CIF file for **HP_3_** (CIF)
CIF file for **P_2_HP** (CIF)
CIF file for **P_2_HP-P_4_** (CIF)
CIF file for **P_3_H** (CIF)

## Supplementary Material

mz3c00304_si_001.pdf

mz3c00304_si_002.cif

mz3c00304_si_003.cif

mz3c00304_si_004.cif

mz3c00304_si_005.cif

mz3c00304_si_006.cif

mz3c00304_si_007.cif

mz3c00304_si_008.cif

mz3c00304_si_009.cif
