# Supplementary material for: Supramolecular Self-Assembly of Engineered Polyproline Helices
Source: ACS Macro Lett. 2023 Jun 26;12(7):908–14. doi: 10.1021/acsmacrolett.3c00304 (PMC10357564; doi:10.1021/acsmacrolett.3c00304)
Supplement: Supplementary file 1 — mz3c00304_si_001.pdf [file mz3c00304_si_001.pdf]

Supporting Information

Supramolecular Self-assembly of Engineered Polyproline Helices

Dominic F Brightwell,[a] Giada Truccolo,[a] Kushal Samanta,[a] Helena J Shepherd[a] and Aniello Palma*[a,b]

a) School of Physical Sciences, Supramolecular Interfacial and Synthetic Chemistry, Ingram building, The University of Kent, Canterbury CT2 7NZ, Kent, United Kingdom

b) School of Chemistry, University College Dublin, Belfield, Dublin 4, Ireland

Emails: a.palma@kent.ac.uk; aniello.palma@ucd.ie
Homepage: https://www.palmaresearchgroup.com/

Abstract

The ability to rationally design biomaterials to form desired supramolecular constructs presents an ever-growing research field, with many burgeoning works within recent years providing exciting results, however, there exists a broad expanse of promising avenues of research yet to be investigated. As such we have set out to make use of the polyproline helix as a rigid, tuneable, and chiral ligand for the design and synthesis of supramolecular constructs. In this investigation we show how an oligoproline tetramer can be specifically designed and functionalised, allowing predictable tuning of supramolecular interactions, to engineer the formation of supramolecular peptide frameworks with varying properties. Consequently, laying the groundwork for further studies utilising the polyproline helix, with the ability to design desired supramolecular structures containing these peptide building-blocks, having tuneable structural features and functionalities.

Table of Contents

SI 1. Synthesis of peptide units: 3
SI 2. Analytical data: 6
SI 2.1. NMR Spectra: 6
SI 2.2. HPLC and Mass spectra: 24
SI 3. X-ray Diffraction data: 40
SI 3.1 Single-crystal XRD data: 40
SI 3.1.1 P_4 SC-XRD: 42
SI 3.2 Powder XRD data: 65
References 72

SI 1. Synthesis of peptide units:

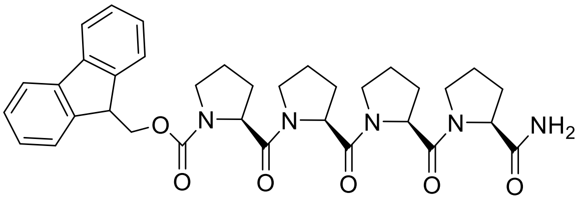

$\mathbf{P_4}$

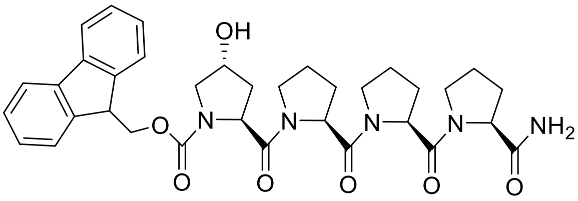

$\mathbf{HP_3}$

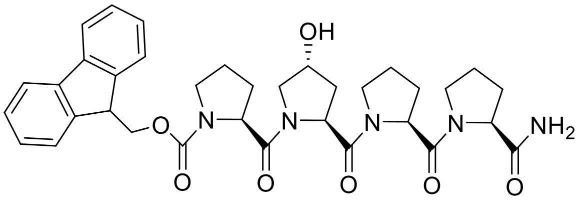

$\mathbf{PHP_2}$

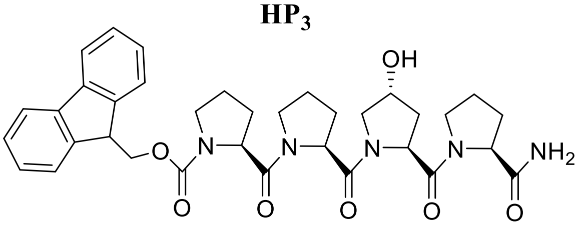

$\mathbf{P_2HP}$

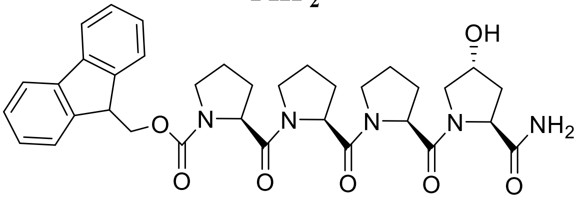

$\mathbf{P_3H}$

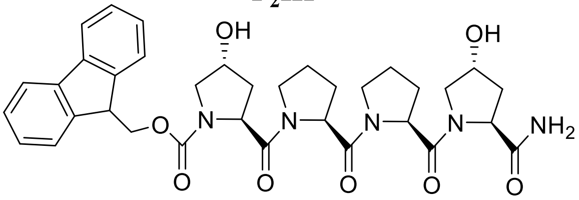

$\mathbf{HP_2H}$

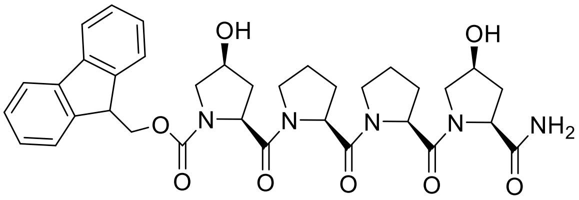

***Cis*-HP$_2$H**

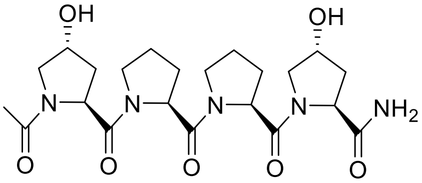

$\mathbf{AcHP_2H}$

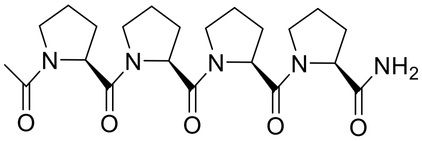

$\mathbf{AcP_4}$

Materials *– Rink Amide MBHA resin (100-200 mesh, 0.3 mmol/g) 1% DVB, Fmoc-L-amino acids, N,N-Diisopropylethylamine (DIPEA), acetic anhydride (Acac) and benzotriazol-1-yl-oxytripyrrolidinophosphonium hexafluorophosphate (PyBOP) were obtained from Fluorochem Ltd (Derbyshire, UK).*

Peptide Synthesiser Method – All peptides were synthesised on a 0.1 mmol scale on Rink amide MBHA resin using a Liberty^{TM} microwave peptide synthesiser (CEM) utilising Fmoc solid-phase peptide synthesis techniques and repeated steps of single deprotections, and couplings interspaced with washings (4 x 4 ml DMF). The synthesis was paused after the final coupling step and the resin removed from the reaction vessel before stopping the synthesis (to prevent gradual loss of the Fmoc group). Deprotection: 20 % piperidine in DMF (4.5 ml) for 5 min with 30 W microwave irradiation at 90 ^{o}C. Coupling: Fmoc-amino acid (1.5 ml, 0.2 M, 3 eq.), DIC (1.2 ml, 0.5 M, 6 eq.), Oxyma Pure (0.6 ml, 0.5 M, 3 eq.) in DMF, and DMF (3 ml) for 5 min at 90 ^{o}C with 30 W microwave irradiation. **Capping procedure:** Resin washed with DMF (x 5) and suspended in DMF with Acac (50 eq) and DIPEA (50 eq). Resin agitated for 30 mins before filtering and washing the resin multiple times with DMF.

Peptide Cleavage - The resin was then washed with DCM (x 5), before the Fmoc-protected peptide was cleaved from the resin with TFA (95 % in DCM) for 1.5 h. The resin was then washed with the cleavage cocktail (x 2) and the filtrate was concentrated by evaporation before precipitation in cold Et_2O and centrifugation. The solution was then decanted and the solid repeatedly washed with cold Et_2O to isolate the peptide, as a white solid in a quantitative yield after drying under vacuum. Peptides were then used without any further purification, > 99 % purity by analytical reverse-phase HPLC.

P_4, **^{1}H NMR** (400 MHz, MeOD) δ 7.85 (dd, *J* = 14.4, 7.3 Hz, 2H), 7.70 – 7.58 (m, 2H), 7.48 – 7.31 (m, 4H), 4.76 – 4.68 (m, 1H), 4.66 – 4.61 (m, 0.5H), 4.58 (dd, *J* = 9.0, 4.1 Hz, 0.5H), 4.46 – 4.36 (m, 2H), 4.34 – 4.25 (m, 1H), 4.19 (t, *J* = 4.6 Hz, 0.5H), 4.08 (dd, *J* = 8.7, 3.2 Hz, 0.5H), 3.87 – 3.78 (m, 1H), 3.78 – 3.70 (m, 1H), 3.63 (q, *J* = 7.0 Hz, 4H), 3.59 – 3.52 (m, 1H), 3.51 – 3.42 (m, 1H), 3.42 – 3.35 (m, 1H), 2.32 – 1.74 (m, 14H). **^{13}C NMR** (101 MHz, MeOD) δ = 177.0, 172.8, 172.6, 172.3, 172.1, 156.6, 156.2, 145.4, 142.6, 128.8, 128.2, 126.2, 125.7, 120.9, 68.7, 67.0, 61.2, 59.6, 59.3, 59.1, 58.3, 48.3, 48.0, 47.8, 30.70, 30.0, 29.1, 28.8, 25.8, 25.2, 24.0, 18.4. FT-IR = ν_{max}/cm^{-1} 2956.88 (C-H), 2881.65 (C-H), 1683.86 (C=Os), 1624.06 (C=Os). m/z calcd. for $[M+H]^+$ $C_{35}H_{42}N_5O_6^+$: 628.3130; found: $[M+H]^+$ 628.3137, $[M+Na]^+$ 650.2938, $[2M+Na]^+$ 1277.6010

HP_3, ^{1}H NMR (400 MHz, MeOD) δ 7.83 (dd, *J* = 15.9, 7.7 Hz, 2H), 7.67 – 7.57 (m, 2H), 7.47 – 7.37 (m, 2H), 7.37 – 7.28 (m, 2H), 4.76 – 4.56 (m, 3H), 4.47 – 4.15 (m, 5H), 3.90 – 3.43 (m, 7H), 3.09 – 3.00 (m, 1H), 2.35 – 2.12 (m, 4H), 2.12 – 1.84 (m, 10H). ^{13}C NMR (101 MHz, MeOD) δ 177.01, 172.61, 172.55, 172.26, 172.03, 156.50, 145.15, 142.59, 128.85, 128.77, 128.19, 126.15, 125.66, 120.99, 120.92, 70.85, 69.81, 68.89, 67.26, 61.23, 59.81, 59.65, 59.58, 59.42, 58.00, 56.27, 56.11, 38.99, 30.73, 29.22, 29.10, 28.84, 25.91, 25.67. m/z calcd. for $[M+H]^+$ $C_{35}H_{42}N_5O_7^+$: 644.3079; found: $[M+H]^+$ 644.2, $[M+Na]^+$ 666.1 , $[2M+Na]^+$

PHP_2, ^{1}H NMR (400 MHz, MeOD) δ 7.82 (t, *J* = 7.5 Hz, 2H), 7.67 – 7.55 (m, 2H), 7.47 – 7.25 (m, 4H), 4.81 (t, *J* = 7.8 Hz, 0.5H), 4.73 – 4.61 (m, 1.5H), 4.57 (dd, *J* = 8.5, 3.2 Hz, 0.5H), 4.53 – 4.30 (m, 4.5H), 4.25 (t, *J* = 6.3 Hz, 1H), 3.89 – 3.69 (m, 3H), 3.64 (dd, *J* = 10.2, 4.8 Hz, 3H), 3.58 – 3.35 (m, 3H), 2.69 (s, 1H), 2.35 – 2.15 (m, 4H), 2.14 – 1.80 (m, 11H). ^{13}C NMR (101 MHz, MeOD) δ 177.01, 172.93, 172.55, 172.06, 145.43, 142.54, 129.88, 128.80, 128.20, 128.17, 126.14, 126.01, 122.04, 120.94, 120.67, 108.22, 71.26, 70.83, 68.75, 68.49, 61.27, 59.69, 58.58, 56.16, 55.65, 37.31, 30.89, 30.73, 29.26, 25.94. m/z calcd. for $[M+H]^+$ $C_{35}H_{42}N_5O_7^+$: 644.3079; found: $[M+H]^+$ 644.3, $[M+Na]^+$ 666.3, $[2M+H]^+$ 1287.8, $[2M+H+Na]^{2+}$ 1310.6

P_2HP, ^{1}H NMR (400 MHz, MeOD) δ 7.83 (dd, *J* = 15.5, 7.7 Hz, 2H), 7.67 – 7.55 (m, 2H), 7.47 – 7.28 (m, 4H), 4.81 – 4.67 (m, 2H), 4.63 – 4.47 (m, 2H), 4.46 – 4.32 (m, 2H), 4.32 – 4.23 (m, 1H), 4.17 (t, *J* = 4.6 Hz, 0.5H), 4.07 (dd, *J* = 8.8, 3.3 Hz, 0.5H), 3.89 – 3.69 (m, 3H), 3.69 – 3.64 (m, 1H), 3.56 – 3.38 (m, 2H), 2.97 (dd, *J* = 11.8, 5.0 Hz, 1H), 2.33 – 1.68 (m, 14H). ^{13}C NMR (101 MHz, MeOD) δ 176.95, 172.45, 145.69, 145.11, 142.65, 128.77, 128.18, 126.14, 125.62, 120.90, 71.16, 68.76, 67.02, 61.31, 59.37, 59.06, 37.54, 30.77, 30.10, 28.76, 25.87, 25.64, 24.07. m/z calcd. for $[M+H]^+$ $C_{35}H_{42}N_5O_7^+$: 644.3079; found: $[M+H]^+$ 644.3, $[M+Na]^+$ 666.2, $[2M+2H]^{2+}$ 1288.6, $[2M+H+Na]^{2+}$ 1310.2

P_3H, ^{1}H NMR (400 MHz, MeOD) δ 7.87 (dd, *J* = 16.1, 8.2 Hz, 2H), 7.71 – 7.59 (m, 2H), 7.53 – 7.44 (m, 2H), 7.43 – 7.34 (m, 2H), 4.75 – 4.61 (m, 2H), 4.60 – 4.53 (m, 1H), 4.53 – 4.39 (m, 2H), 4.31 – 4.05 (m, 2H), 3.86 – 3.74 (m, 3H), 3.71 – 3.57 (m, 2H), 3.22 (s, 1H), 2.69 (dd, *J* = 16.0, 6.3 Hz, 1H), 2.35 – 2.13 (m, 4H), 2.12 – 2.01 (m, 4H), 2.00 – 1.68 (m, 7H). ^{13}C NMR (101 MHz, MeOD) δ 172.80, 157.66, 146.53, 145.16, 142.68, 128.76, 128.17, 126.15, 125.59, 120.96, 120.90, 71.55, 71.06, 68.75, 67.78, 66.99, 60.13, 59.67, 59.36, 59.06, 56.08, 38.99, 29.10, 25.71, 24.06. m/z calcd. for $[M+H]^+$ $C_{35}H_{42}N_5O_7^+$: 644.3079; found: $[M+H]^+$ 644.2, $[2M+Na]^+$ 1309.7

HP_2H, ^{1}H NMR (400 MHz, MeOD) δ 7.83 (dd, *J* = 15.9, 7.8 Hz, 2H), 7.68 – 7.57 (m, 2H), 7.42 (dt, *J* = 12.1, 7.4 Hz, 2H), 7.37 – 7.28 (m, 2H), 4.76 – 4.56 (m, 3H), 4.48 (dt, *J* = 10.7, 7.8 Hz, 2H), 4.37 – 4.22 (m, 2.5H), 4.18 (t, *J* = 4.8 Hz, 0.5H), 3.89 – 3.61 (m, 5H), 3.50 (dt, *J* = 18.0, 11.3 Hz, 2H), 3.08 – 2.98 (m, 1H), 2.40 – 2.13 (m, 4H), 2.13 – 1.81 (m, 8H). ^{13}C NMR (101 MHz, MeOD) δ 176.82, 172.66, 172.61, 172.27, 172.08, 156.49, 145.53, 145.16, 142.59, 142.59, 128.77, 128.19, 128.19, 120.99, 71.08, 71.08, 69.80, 67.22, 60.13, 59.65, 59.43, 57.95, 57.95, 56.25, 56.11, 38.99, 28.89, 27.28, 25.87, 25.79, 25.74, 25.65. m/z calcd. for $[M+H]^+$ $C_{35}H_{42}N_5O_8^+$: 660.3028; found: $[M+H]^+$ 660.2, $[M+Na]^+$ 682.2, $[2M+H]^+$ 1319.6, $[2M+H+Na]^{2+}$ 1342.3

$AcHP_2H$, ^{1}H NMR (400 MHz, MeOD) δ 4.79 – 4.65 (m, 3H), 4.54 – 4.43 (m, 3H), 3.91 – 3.49 (m, 8H), 2.53 – 2.16 (m, 5H), 2.13 – 1.97 (m, 10H). ^{13}C NMR (101 MHz, MeOD) δ 204.99, 200.82, 200.63, 200.43, 200.27, 99.26, 99.13, 88.29, 87.90, 86.22, 85.52, 84.26, 67.15, 66.30, 57.36, 57.29, 54.01, 53.96, 50.34. m/z calcd. for $[M+H]^+$ $C_{22}H_{34}N_5O_7^+$: 480.2453 ; found: $[M+H]^+$ 480.2, $[M+Na]^+$ 502.2

***Cis*-HP_2H**, ^{1}H NMR (400 MHz, MeOD) δ 7.85 (dd, *J* = 15.0, 7.5 Hz, 2H), 7.68 – 7.58 (m, 2H), 7.43 (dt, *J* = 13.0, 6.2 Hz, 2H), 7.35 (dd, *J* = 13.4, 6.0 Hz, 2H), 4.79 – 4.71 (m, 1H), 4.64 (ddd, *J* = 15.1, 10.2, 4.3 Hz, 2H), 4.49 – 4.37 (m, 3H), 4.37 – 4.14 (m, 3H), 3.94 (ddd, *J* = 15.0, 10.6, 5.0 Hz, 1H), 3.88 – 3.71 (m, 2H), 3.64 (dt, *J* = 13.4, 6.4 Hz, 4H), 3.58 – 3.38 (m, 2H), 2.54 – 2.23 (m, 4H), 2.20 – 1.77 (m, 10H). ^{13}C NMR (101 MHz, MeOD) δ 177.45, 173.09, 172.96, 156.64, 145.61, 145.29, 145.09, 144.97, 142.67, 128.87, 128.82, 128.26, 128.20, 126.77, 126.11, 125.82, 125.65, 125.53, 120.98, 71.54, 70.30, 68.81, 67.24, 60.40, 59.90, 59.66, 59.53, 58.35, 57.85, 56.46, 56.01, 38.26, 38.11, 37.59, 29.32, 28.87, 25.87, 25.67. m/z calcd. for $[M+H]^+$ $C_{35}H_{42}N_5O_8^+$: 660.3028 ; found: $[M+H]^+$ 660.3, $[M+Na]^+$ 680.3

AcP_4, ^{1}H NMR (400 MHz, $CDCl_3$) δ 8.29 (s, 0.5H), 6.78 (s, 0.5H), 5.68 (s, 0.5H), 5.41 (s, 0.5H), 4.79 – 4.61 (m, 2H), 4.58 (d, *J* = 6.3 Hz, 0.5H), 4.43 (t, *J* = 6.8 Hz, 0.5H), 4.28 (d, *J* = 7.9 Hz, 0.5H), 3.93 – 3.43 (m, 7H), 2.69 (s, 3H), 2.58 (dd, *J* = 12.5, 6.2 Hz, 0.5H), 2.35 – 2.26 (m, 0.5H), 2.26 – 2.10 (m, 5H), 2.09 (s, 3H), 2.07 – 1.72 (m, 8H). ^{13}C NMR (101 MHz, $CDCl_3$) δ 174.34, 173.92, 172.23, 171.27, 170.69, 170.55, 170.02, 60.84, 59.50, 59.05, 58.16, 58.04, 57.95, 48.45, 48.39, 47.54, 47.37, 47.29, 47.16, 46.83, 31.47, 28.80, 28.49, 28.23, 27.89, 27.03, 25.38, 25.31, 25.15, 25.00, 24.91, 24.74, 22.16. m/z calcd. for $[M+H]^+$ $C_{22}H_{34}N_5O_5^+$: 448.2554; found: $[M+H]^+$ 448.5, $[M+Na]^+$ 470.5

SI 2. Analytical data:

SI 2.1. NMR Spectra:

NMR experiments were recorded on a Bruker Avance II 400 MHz spectrometer in MeOD.

SI 2.1.1 P_4 NMR spectra:

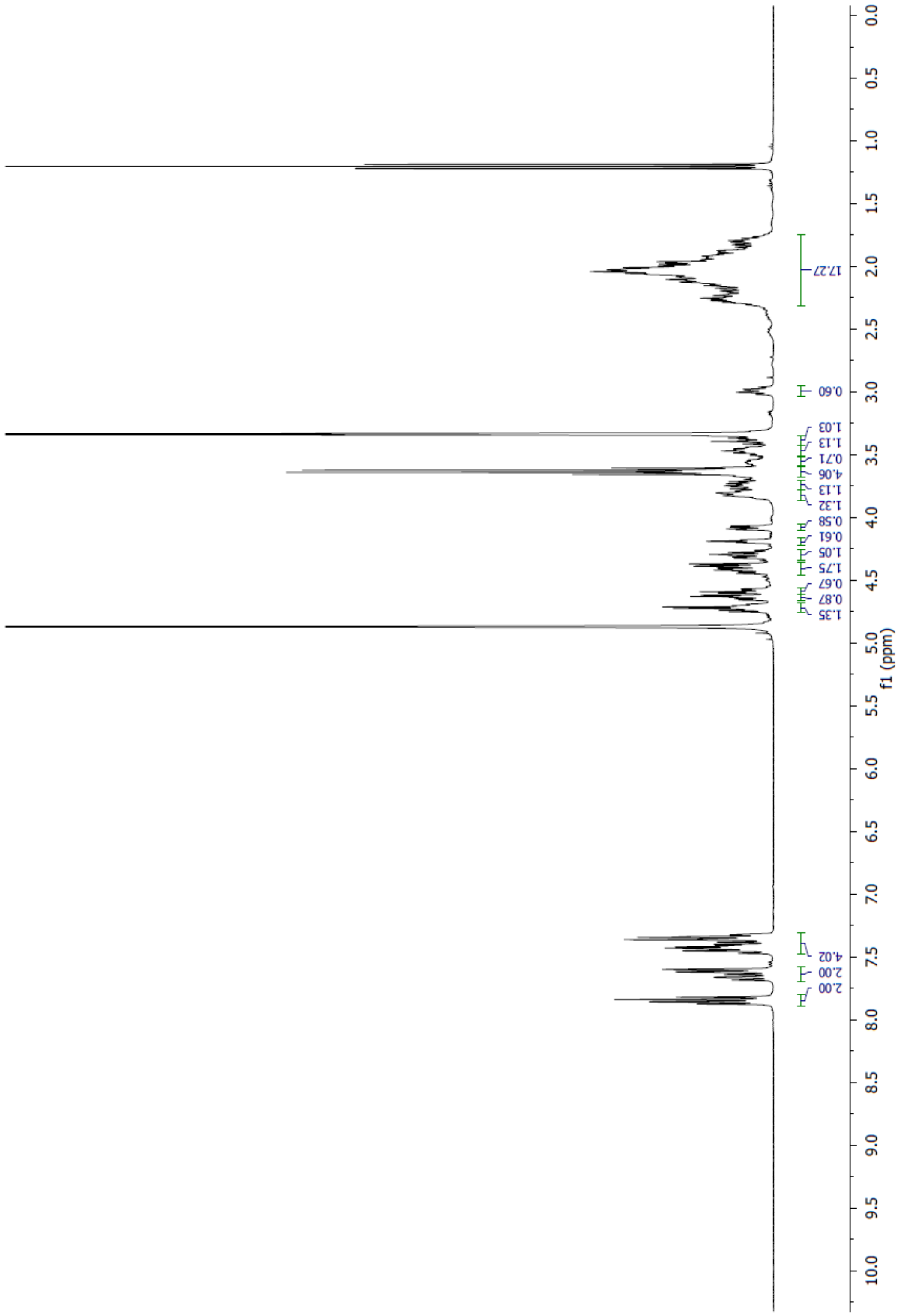

^{1}H NMR of **PP_4 (400 MHz, MeOD)**

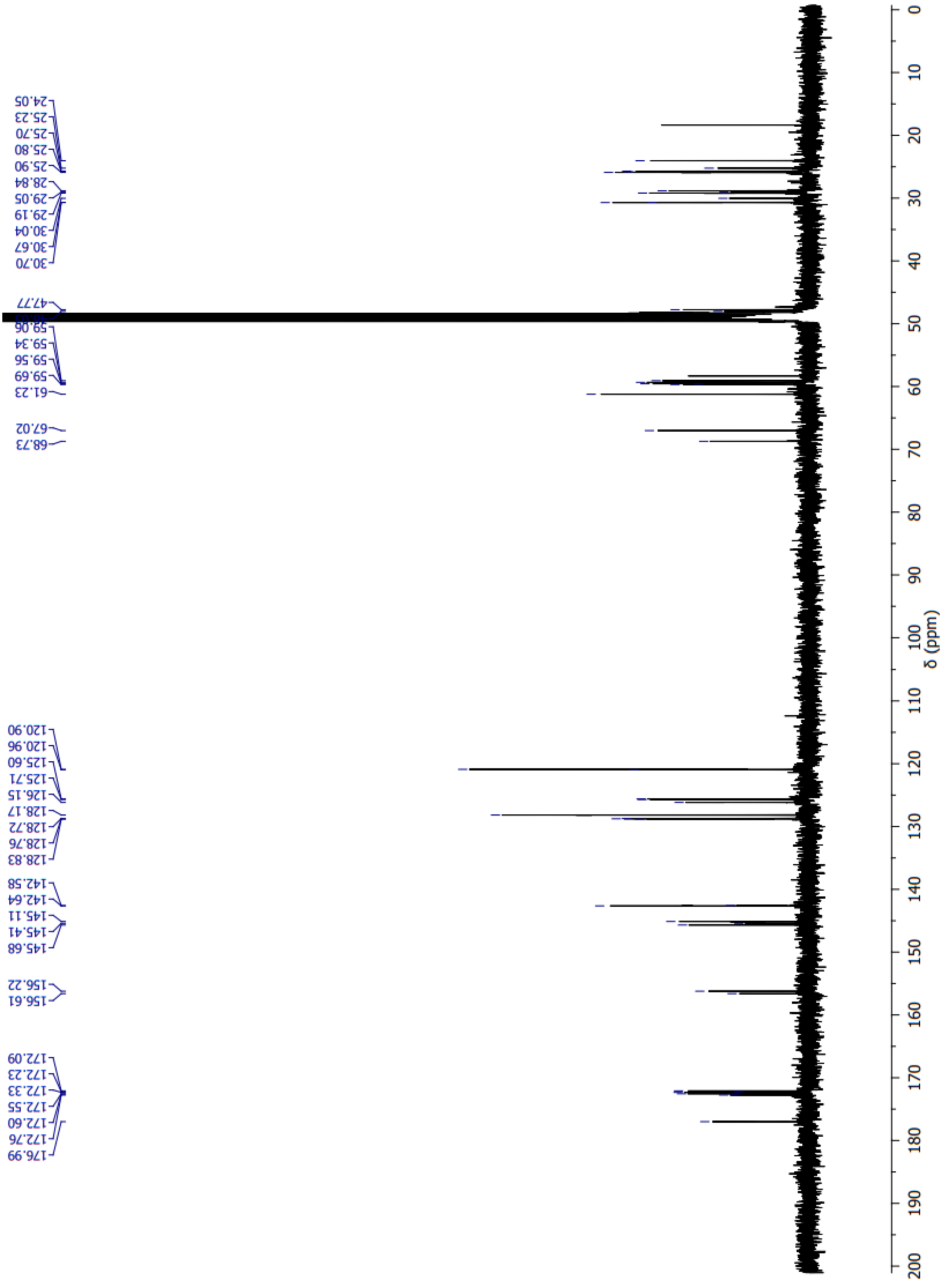

^{13}C NMR of **PP_4 (101 MHz, MeOD)**

SI 2.1.2 HP_3 NMR spectra:

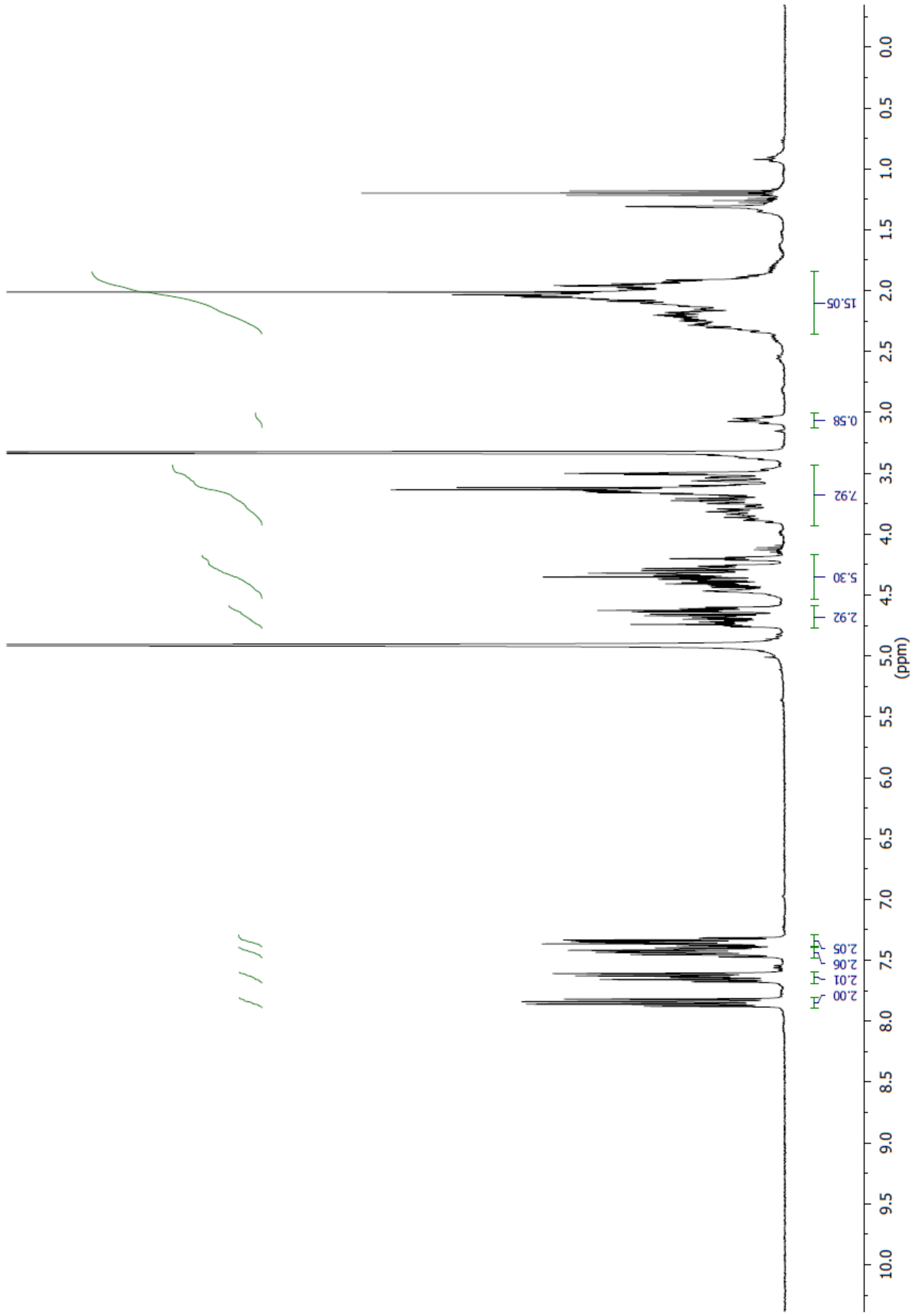

^{1}H NMR of **HP_3 (400 MHz, MeOD)**

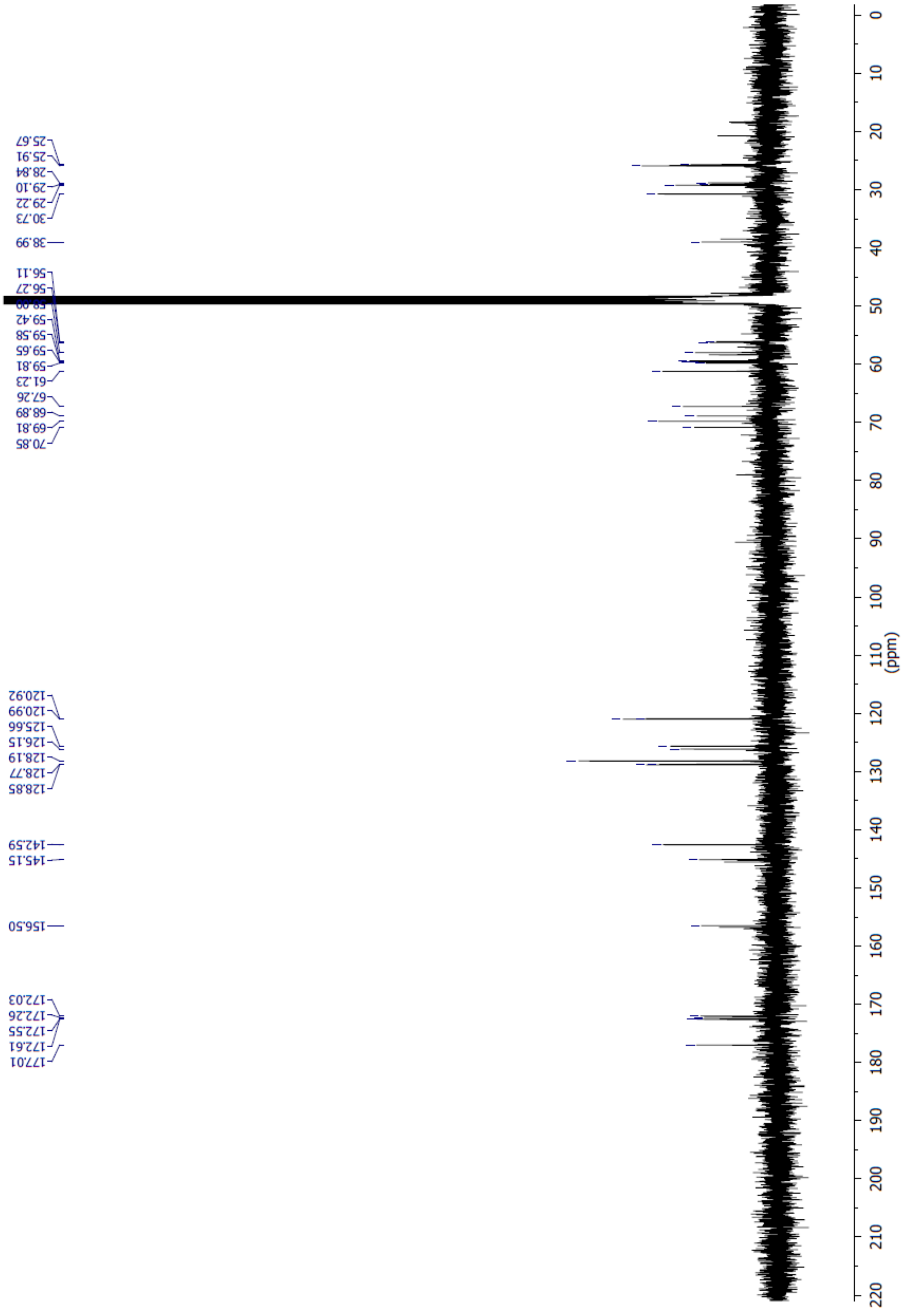

^{13}C NMR of **HP_3 (101 MHz, MeOD)**

SI 2.1.3 PHP$_2$ NMR spectra:

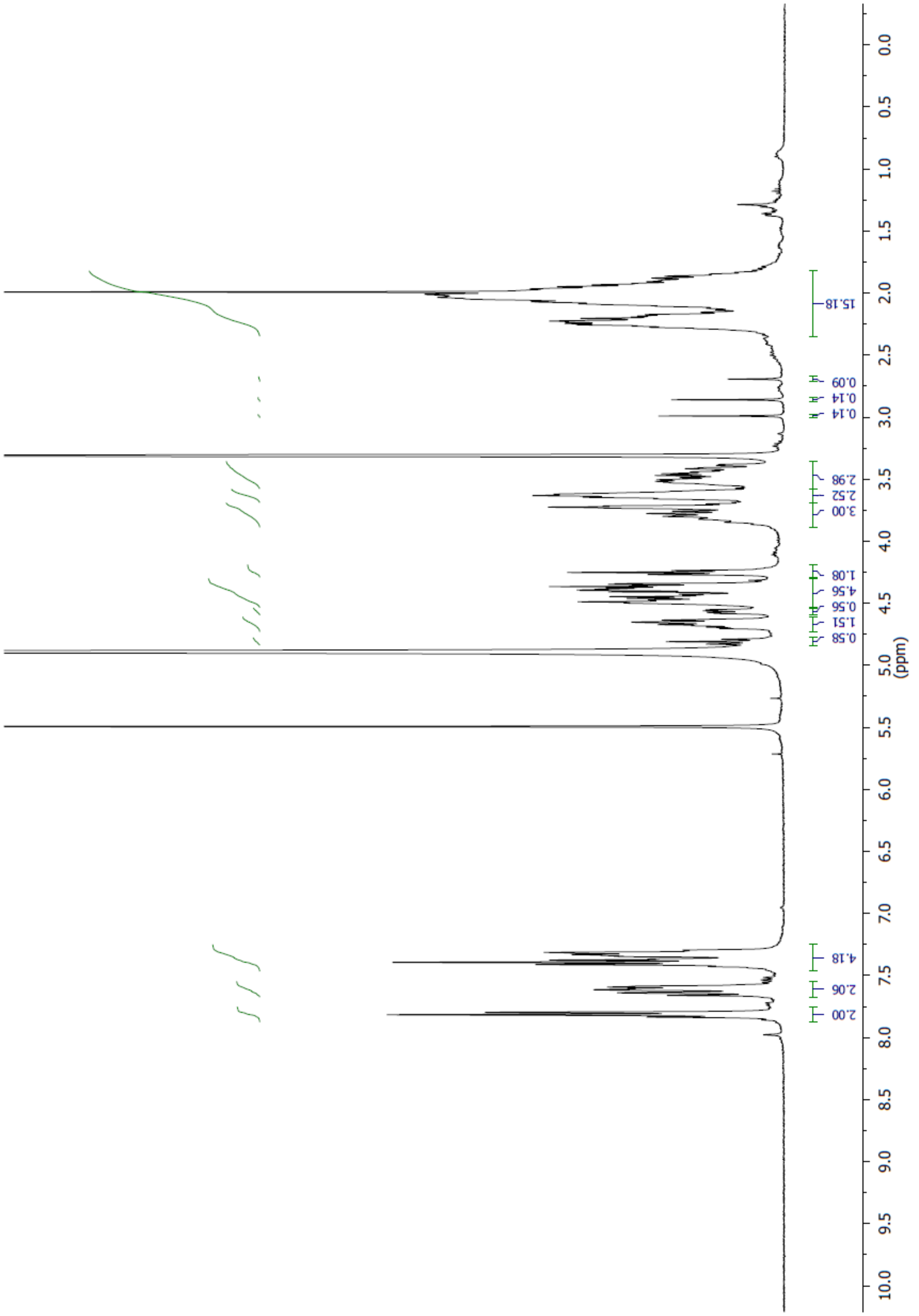

^{1}H NMR of **PHP$_2$ (400 MHz, MeOD)**

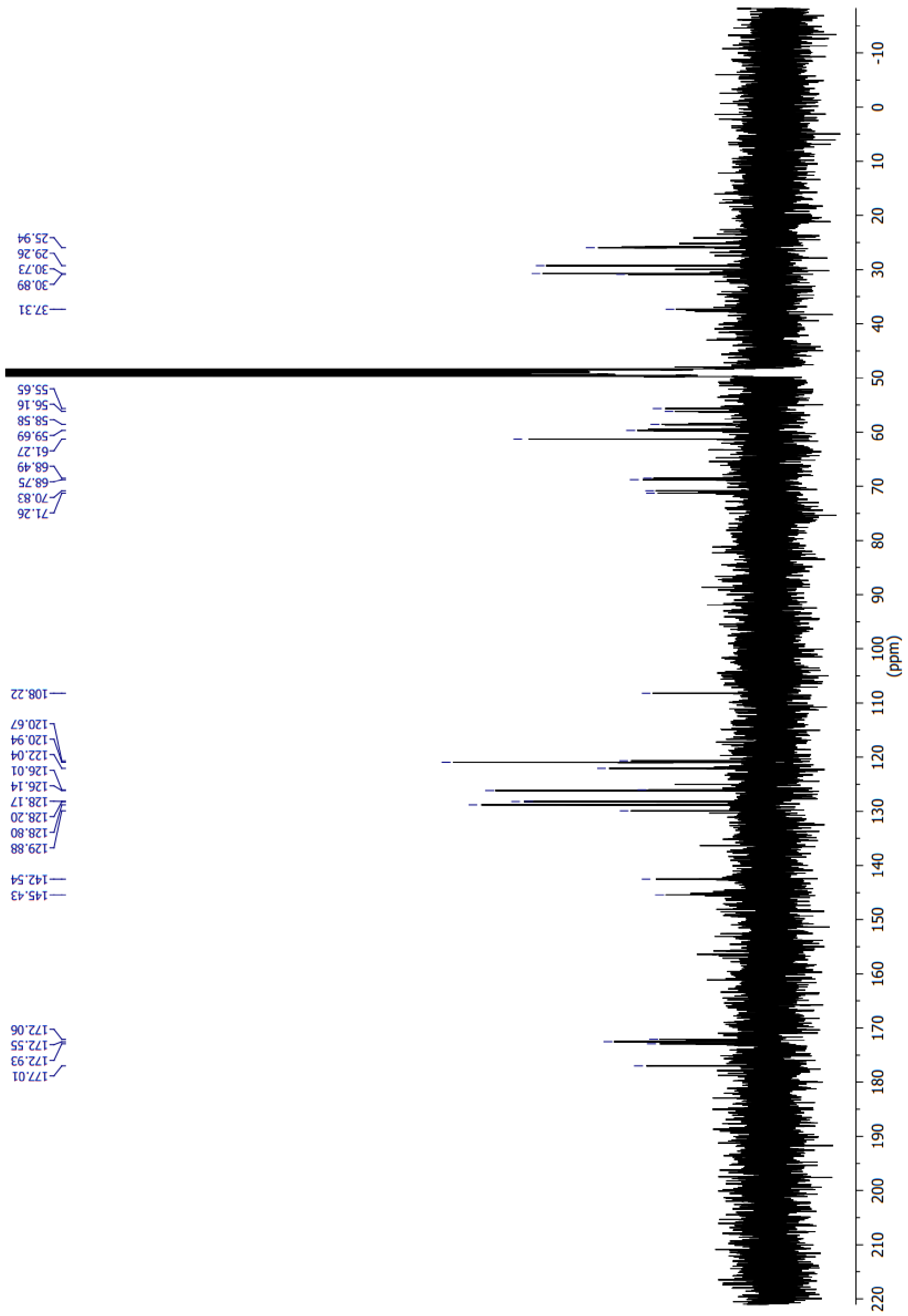

^{13}C NMR of **PHP_2 (101 MHz, MeOD)**

SI 2.1.4 P_2HP NMR spectra:

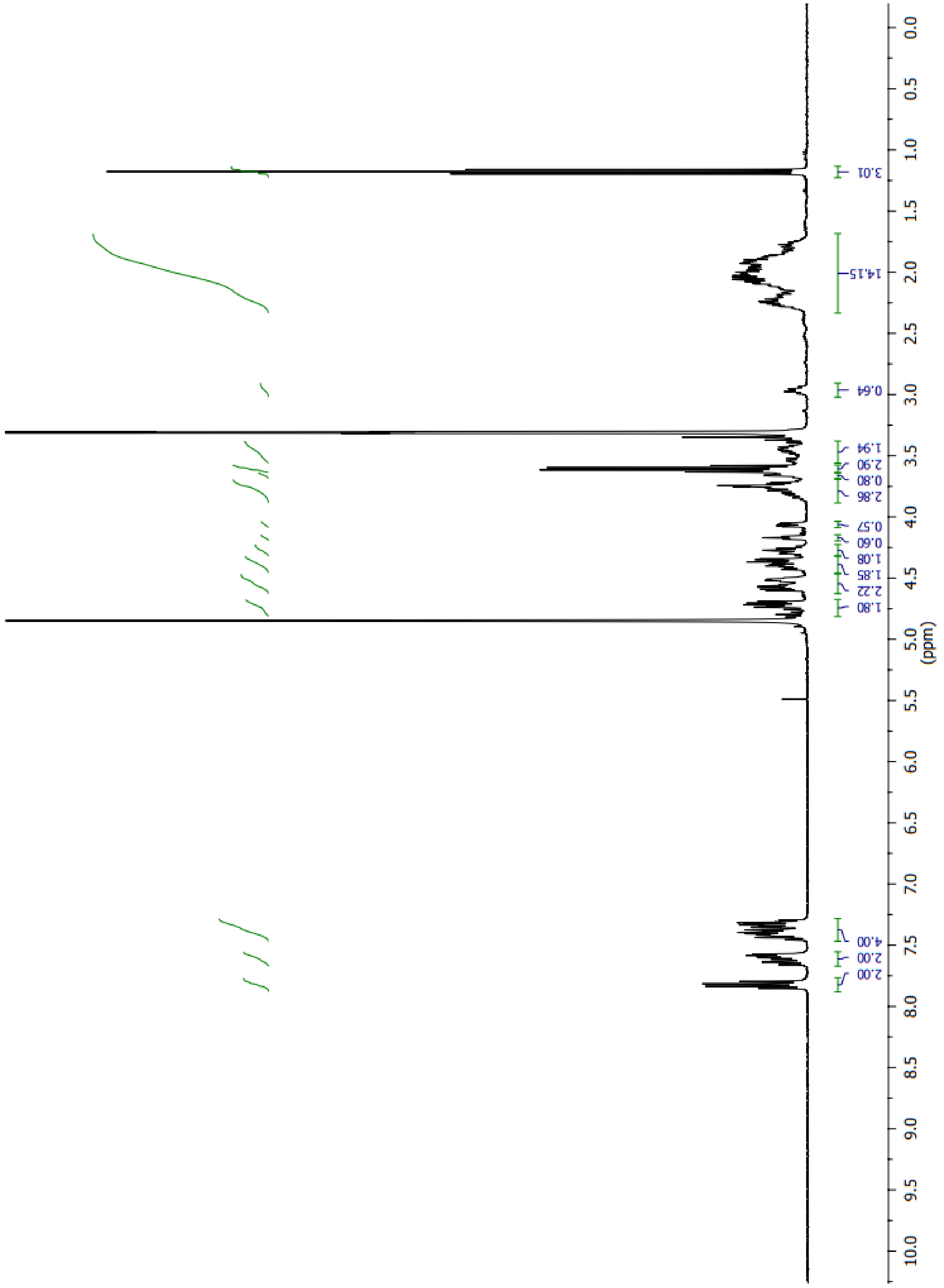

^{1}H NMR of **P_2HP (400 MHz, MeOD)**

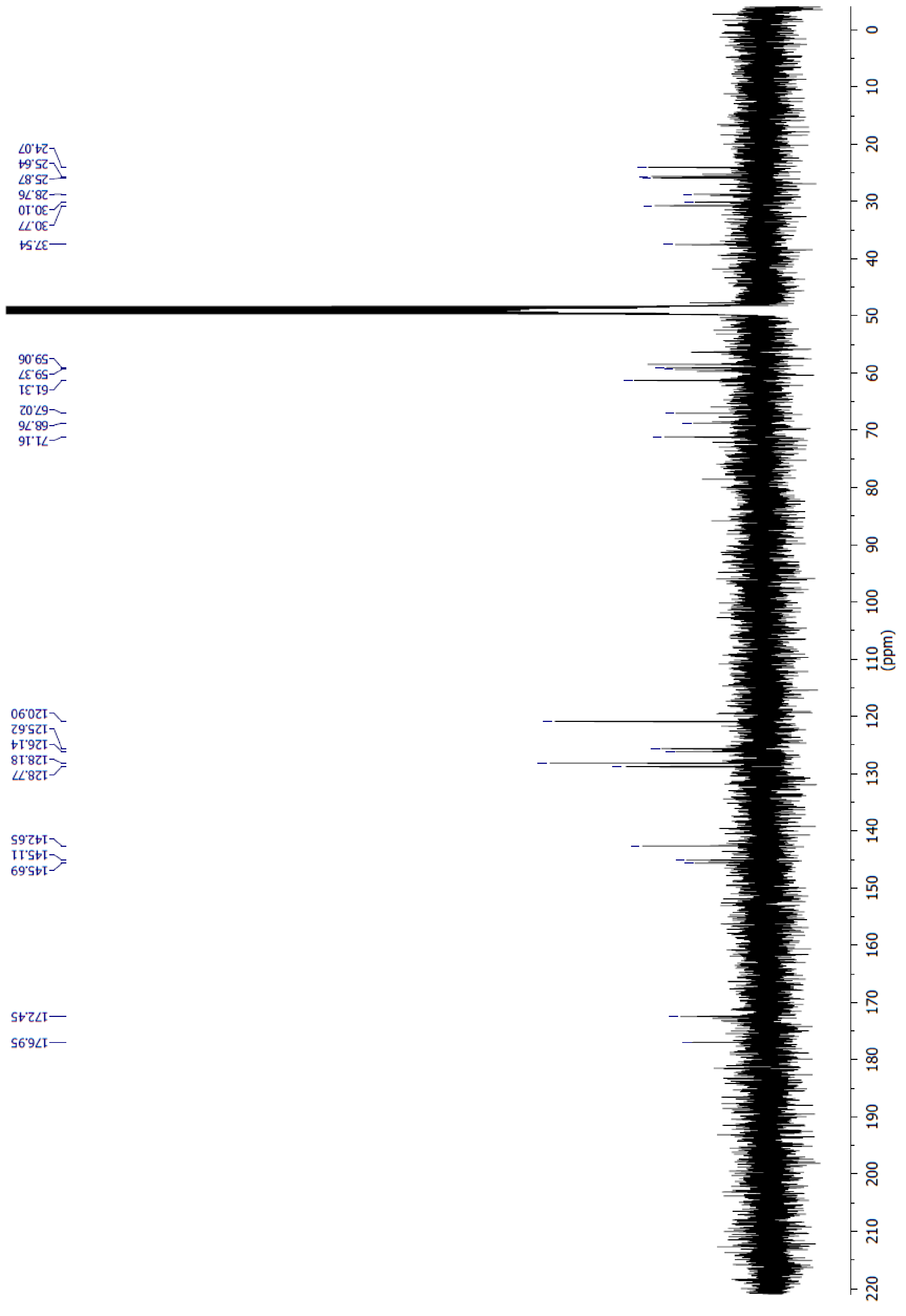

^{13}C NMR of **P_2HP (101 MHz, MeOD)**

SI 2.1.5 P_3H NMR spectra:

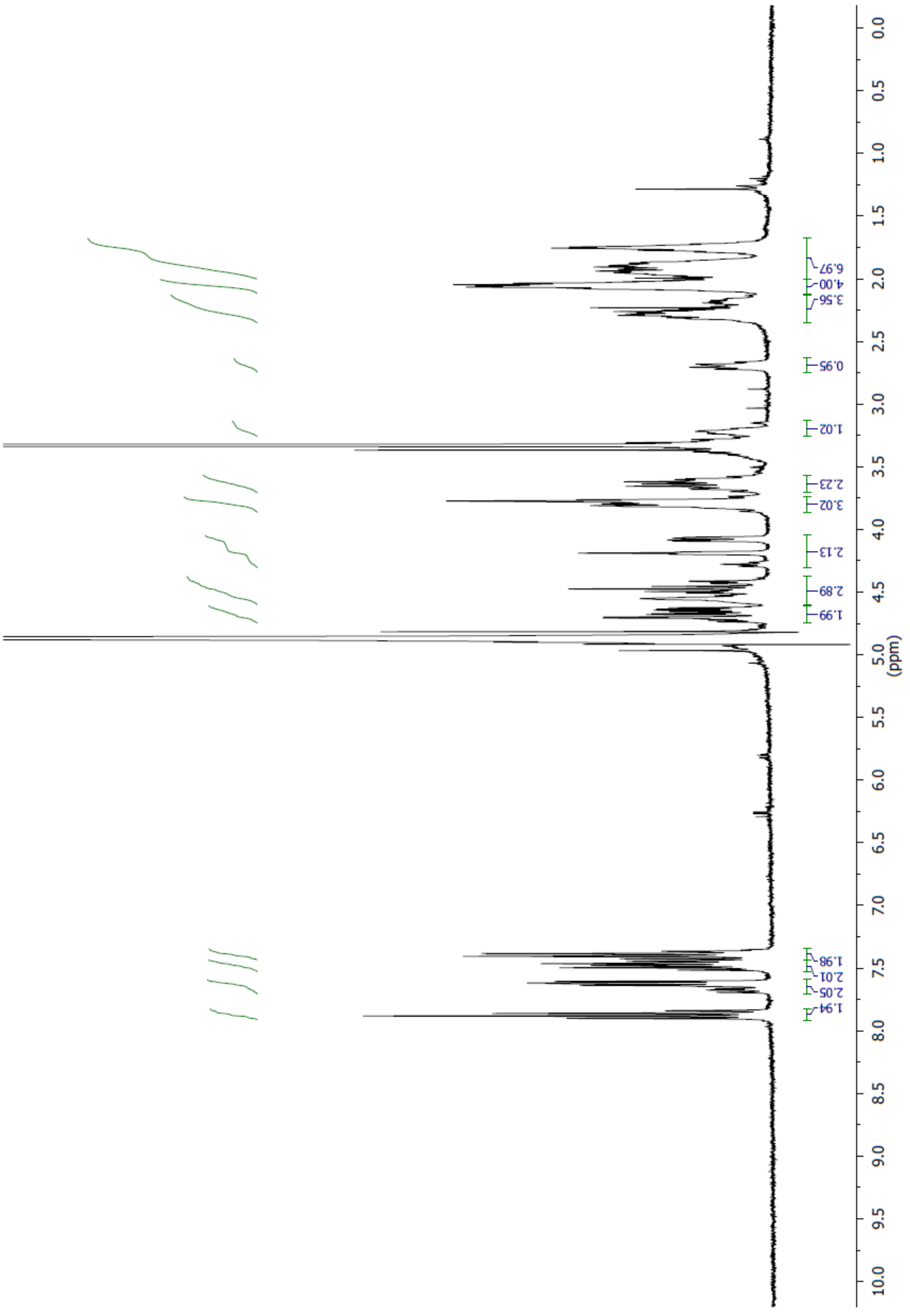

^{1}H NMR of **P_3H (400 MHz, MeOD)**

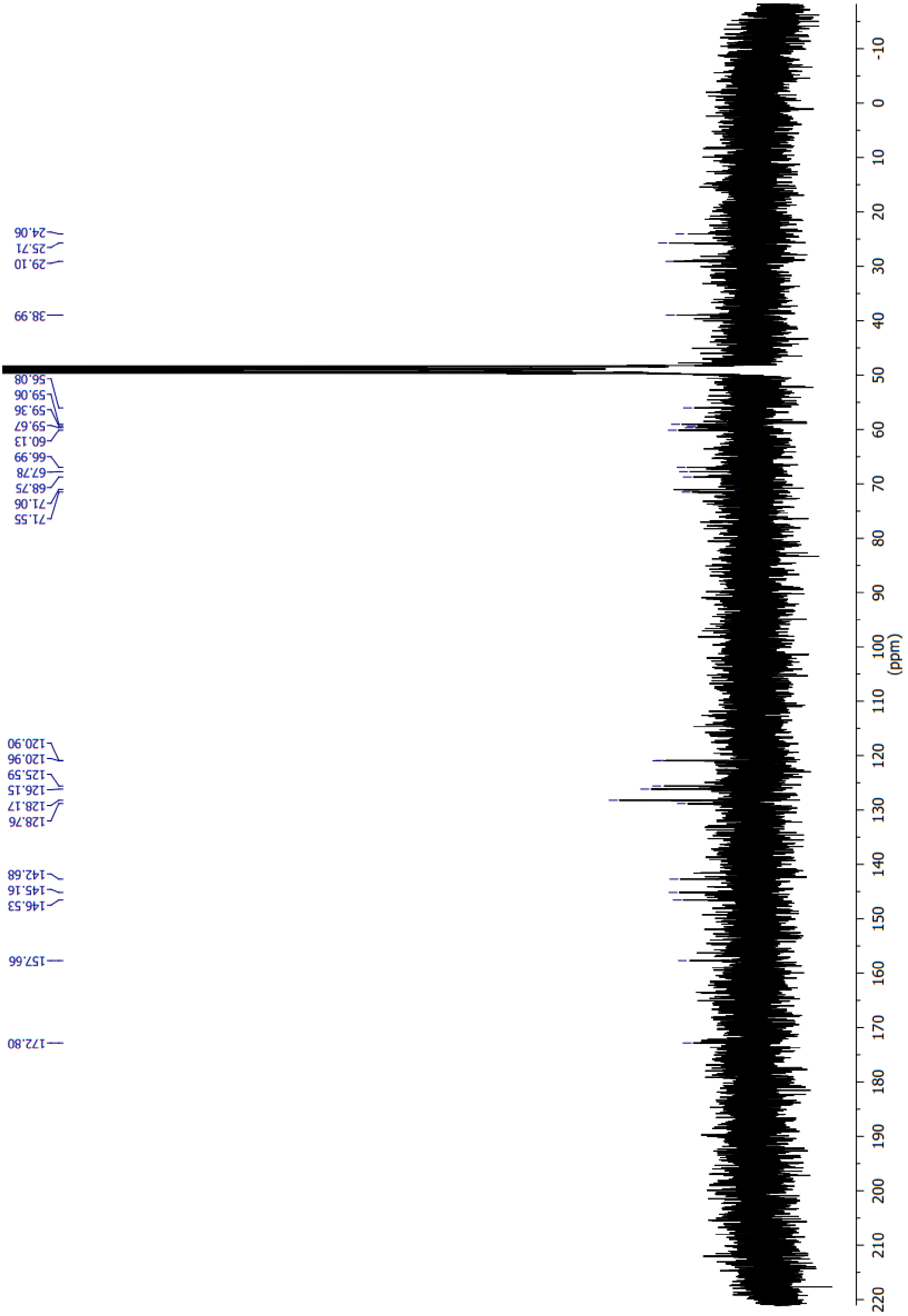

^{13}C NMR of **P_3H (101 MHz, MeOD)**

SI 2.1.6 HP_2H NMR Spectra:

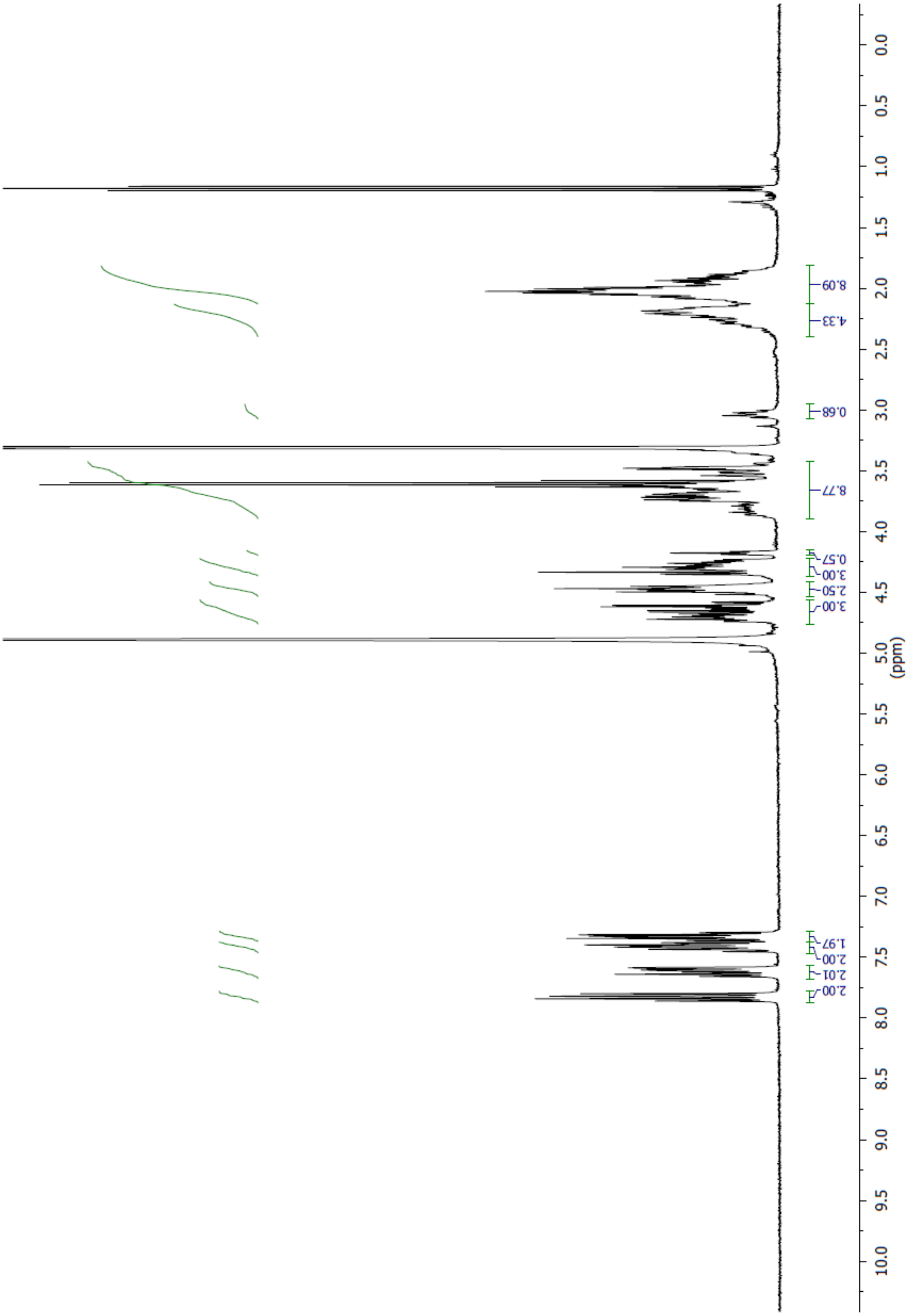

^{1}H NMR of **HP_2H (400 MHz, MeOD)**

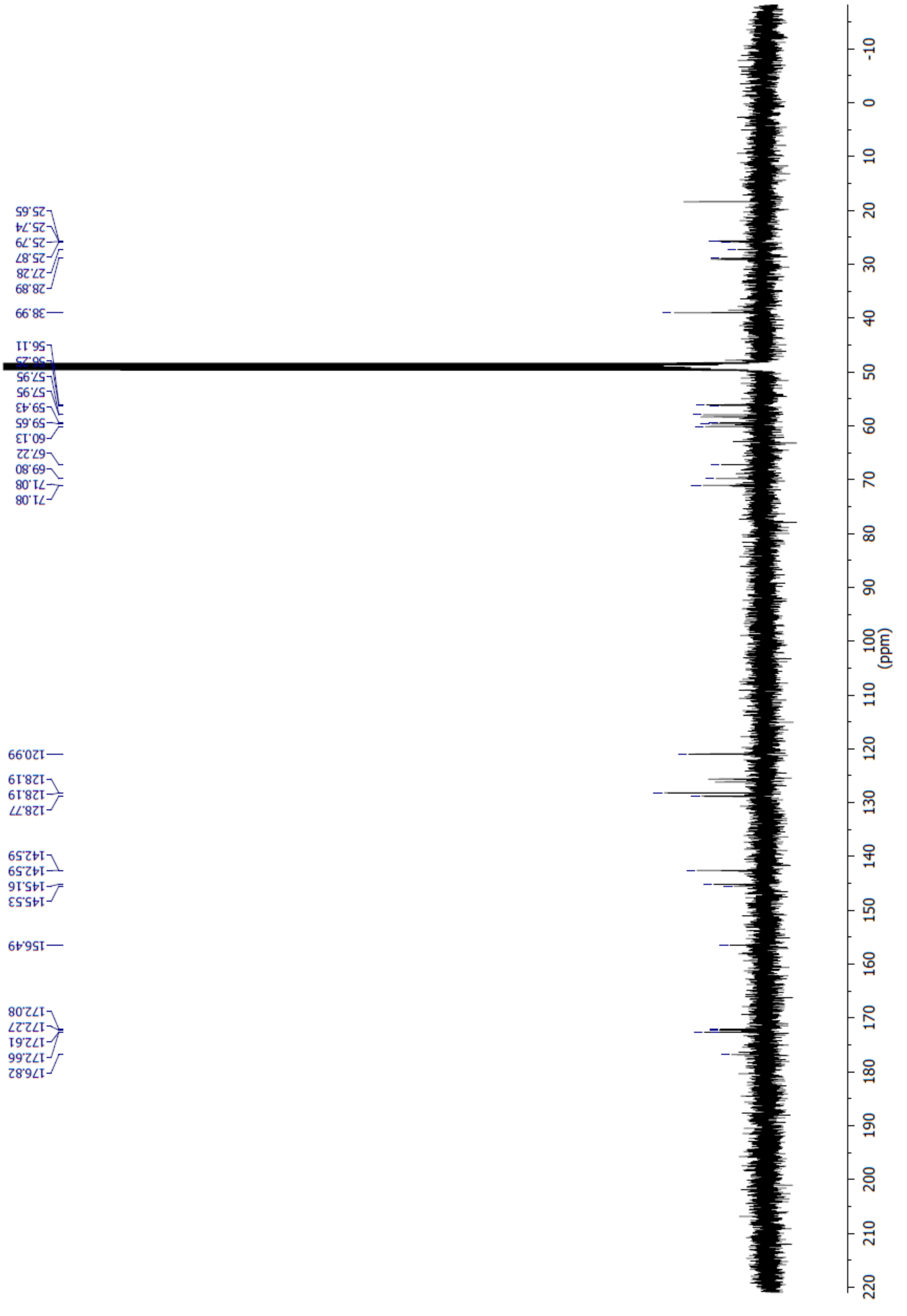

^{13}C NMR of **HP$_2$H (101 MHz, MeOD)**

SI 2.1.7 AcHP$_2$H NMR Spectra:

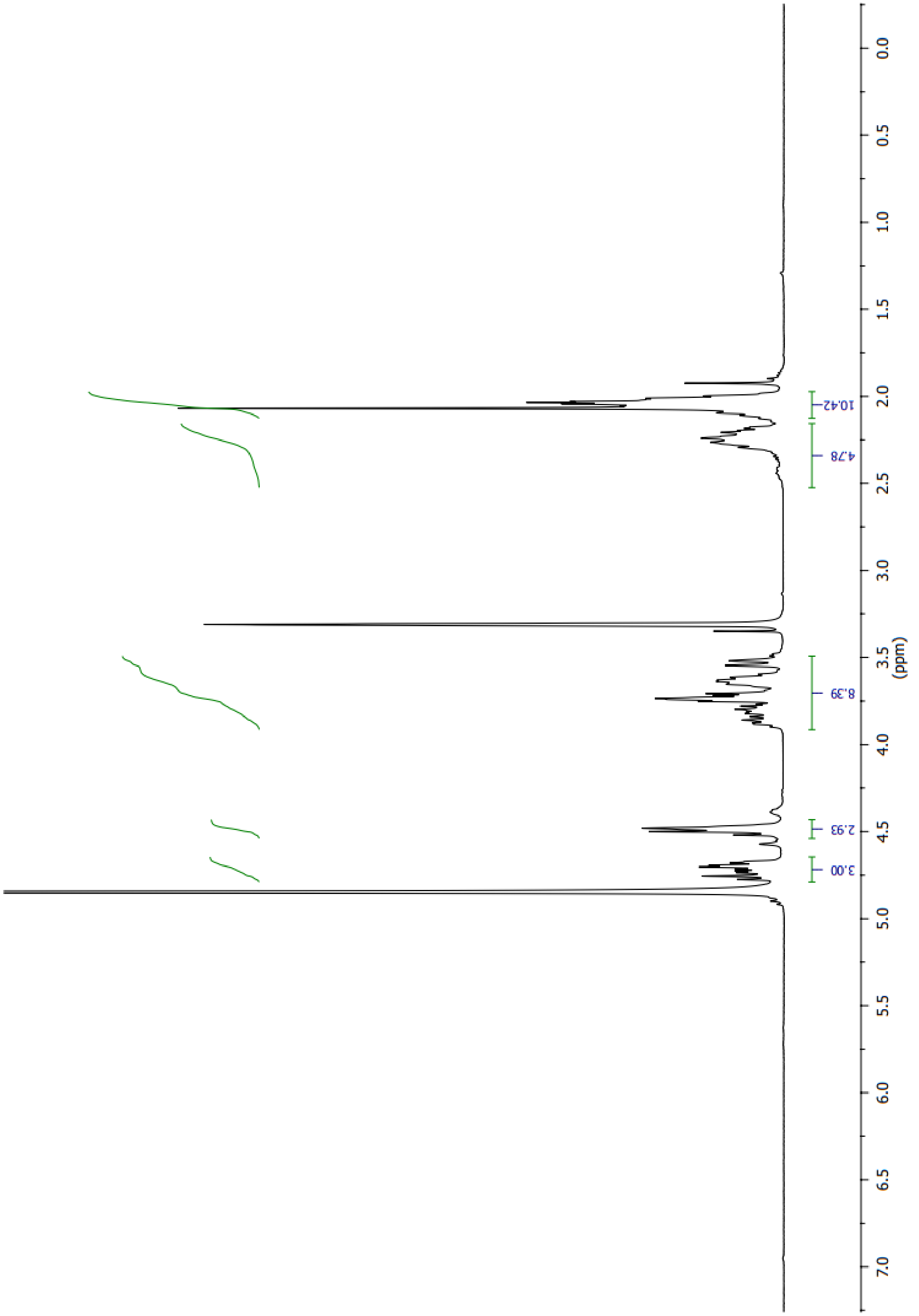

^{1}H NMR of **AcHP$_2$H (400 MHz, MeOD)**

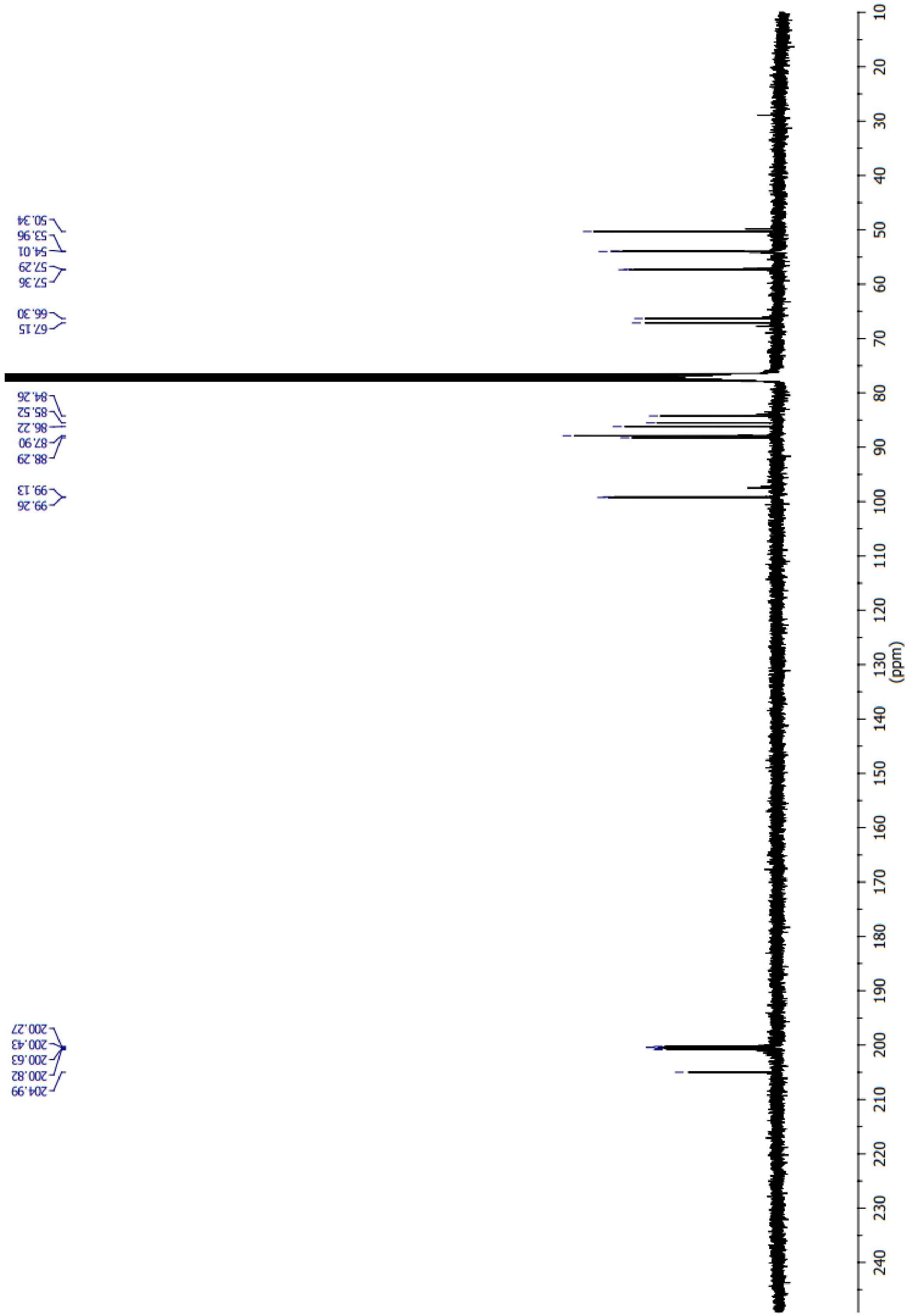

^{13}C NMR of **AcHP$_2$H (101 MHz, MeOD)**

SI 2.1.7 Cis-HP$_2$H NMR Spectra:

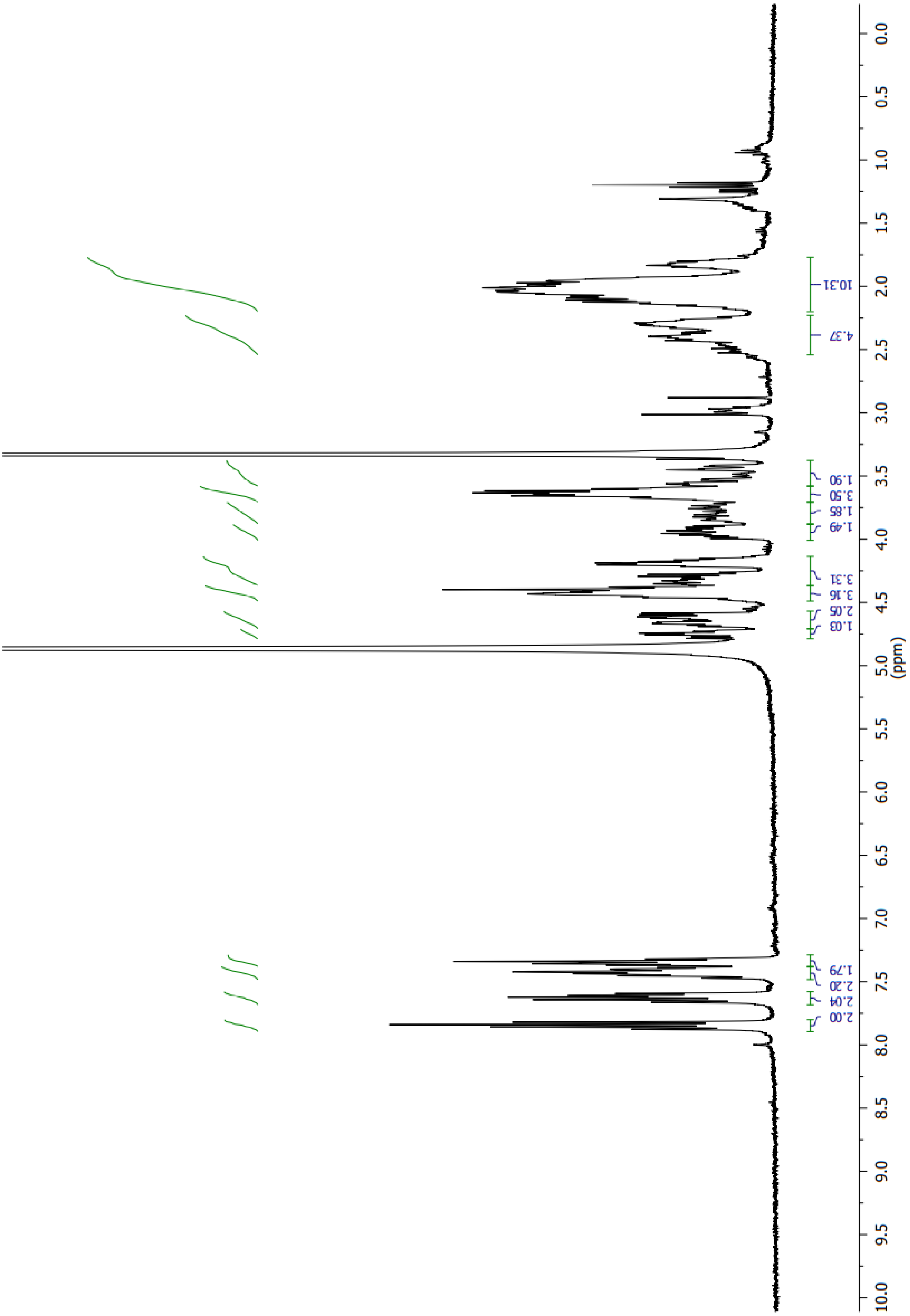

^{1}H NMR of **Cis-HP$_2$H (400 MHz, MeOD)**

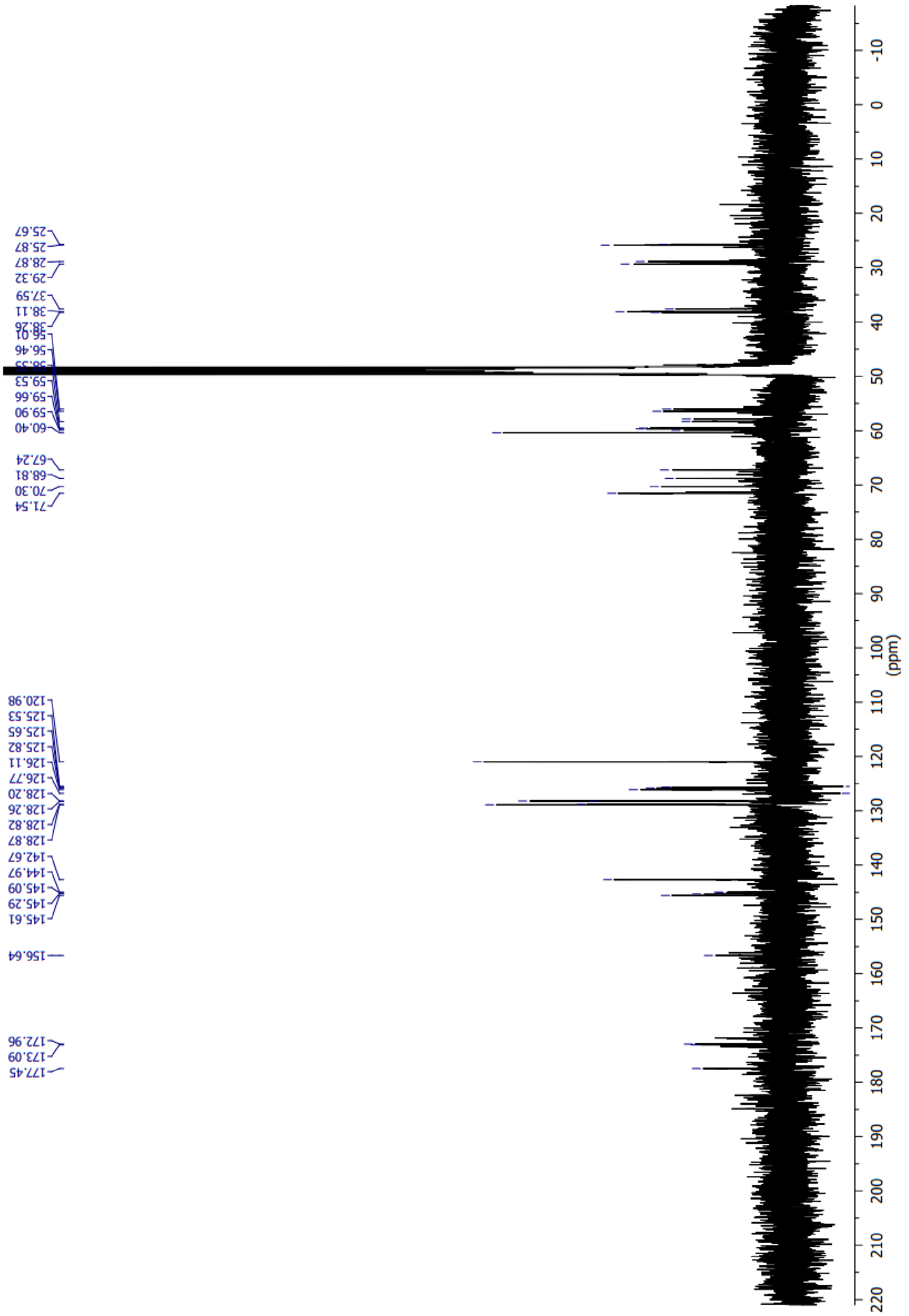

^{13}C NMR of **Cis-HP$_2$H (101 MHz, MeOD)**

SI 2.1.8 AcP$_4$ NMR Spectra:

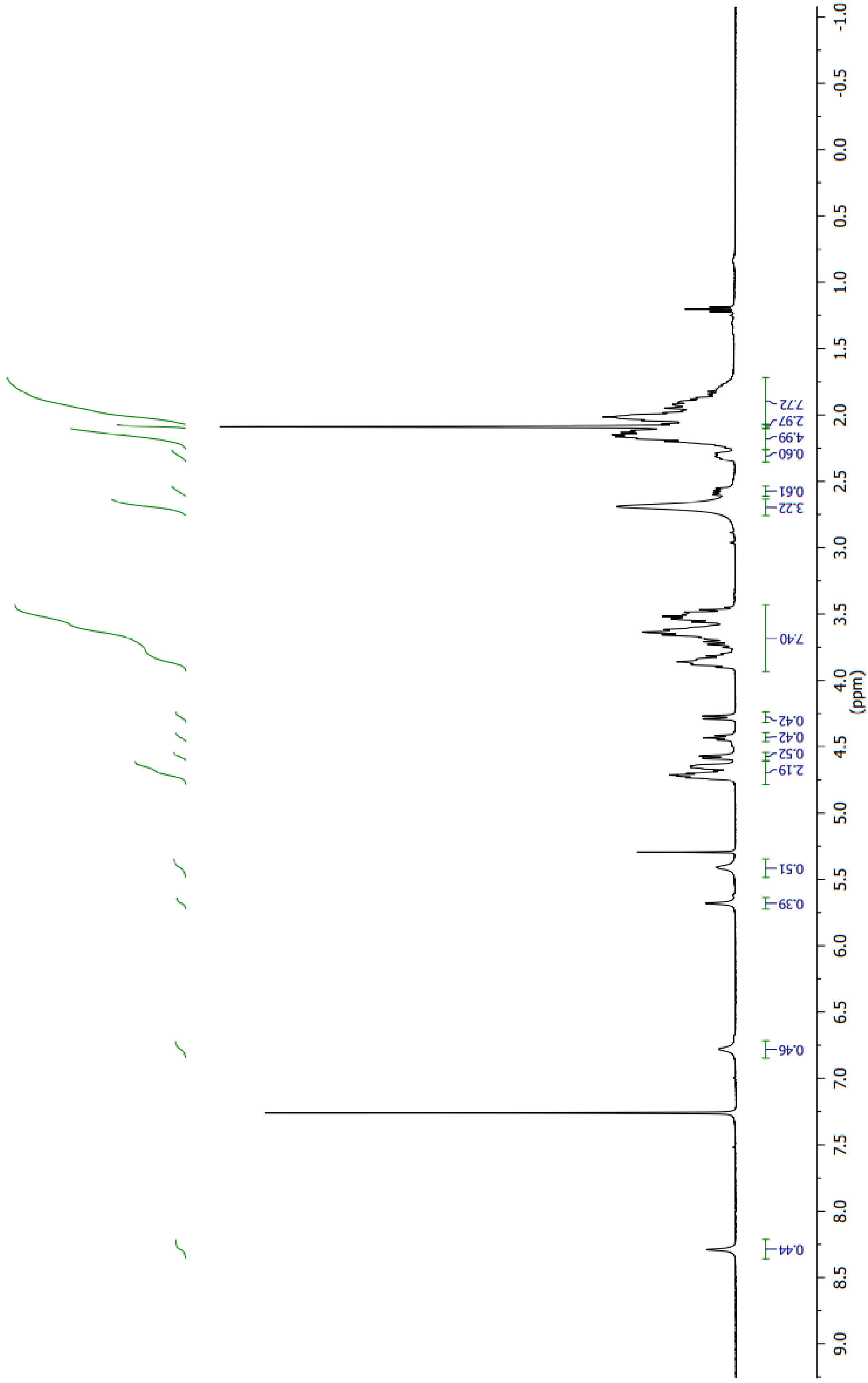

^{1}H NMR of **AcP$_4$ (400 MHz, CDCl$_3$)**

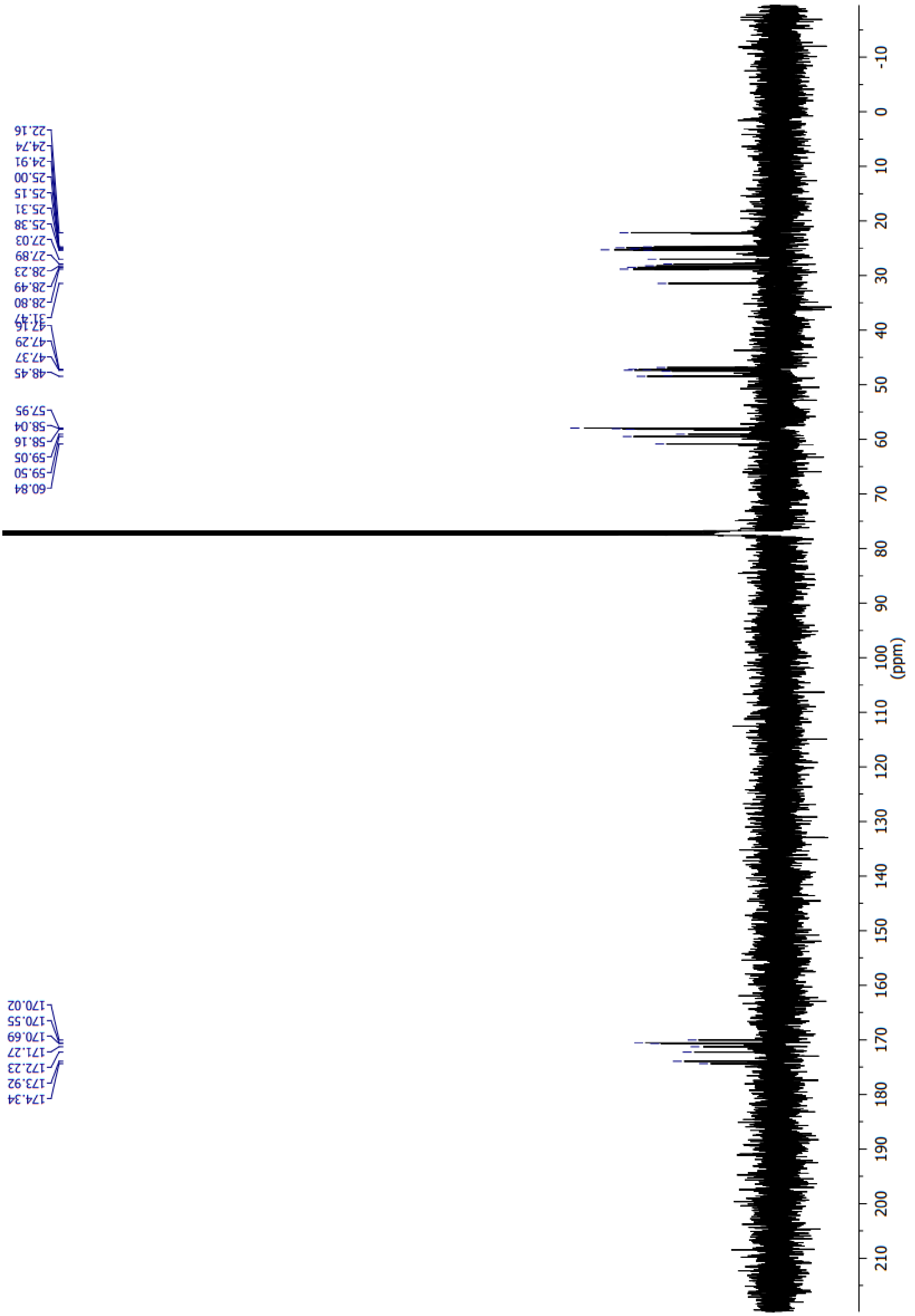

^{13}C NMR of **AcP_4 (101 MHz, $CDCl_3$)**

SI 2.2. HPLC and Mass spectra:

The peptides **P_4** and **P_2HP** were separated on a 2.1 x 150 mm, 3.6 um, XB-C18 Aeris Widepore column, from Phenomenex, at 50 °C on a Waters H-Class Acquity UPLC. Gradient: 5% B for 2.55 minutes then from 5% B to 95% B over 15 minutes, and held at 95% B for 2 minutes. Where A is Water (0.1 % formic acid) and B is acetonitrile (0.1 % formic acid). Flow rate was 0.25 ml/min. The flow was directed into directed into the electrospray source of a Waters G2-Si mass spectrometer, operating in positive ion mode, at 2.5 kV and mass spectra recorded from 100-3000 m/z. Data was analysed with Waters Mass Lynx software.

Peptides **P_3H**, **AcHP_2H**, ***Cis*-HP_2H** and **AcP_4** were separated via RP-HPLC using a HiChrom KR100 5C18 5263 column at 40 °C on a Dionex UltiMate 3000. Gradient: 5% B for 5 minutes then from 5% B to 100% B over 20 minutes, and held at 100% B for 5 minutes. Where A is Water (0.1 % formic acid) and B is methanol (0.1 % formic acid). Flow rate is 1.0 ml/min. Wavelength: 225 nm. The flow was directed into directed into the electrospray source of a Thermo Scientific MSQ Plus Mass Detector, operating in positive ion mode, at 75 kV and mass spectra recorded from 100-2000 m/z.

All other peptides, unless otherwise stated, were separated on a 2.1 x 150 mm, 3.6 um, XB-C18 Aeris Widepore column, from Phenomenex, at 30 °C on an Agilent 1100 HPLC. Gradient: 5% B for 5 minutes then from 5% B to 100% B over 25 minutes, and held at 100% B for 5 minutes. Where A is Water (0.1 % formic acid) and B is acetonitrile (0.1 % formic acid). Flow rate is 0.2 ml/min. The flow was directed into directed into the electrospray source of a Bruker micrOTOF-QII mass spectrometer, operating in positive ion mode, at 4.5 kV and mass spectra recorded from 150-3000 m/z. Data was analysed with Bruker's Compass Data Analysis software.

SI 2.2.1 HRMS Spectra – P_4

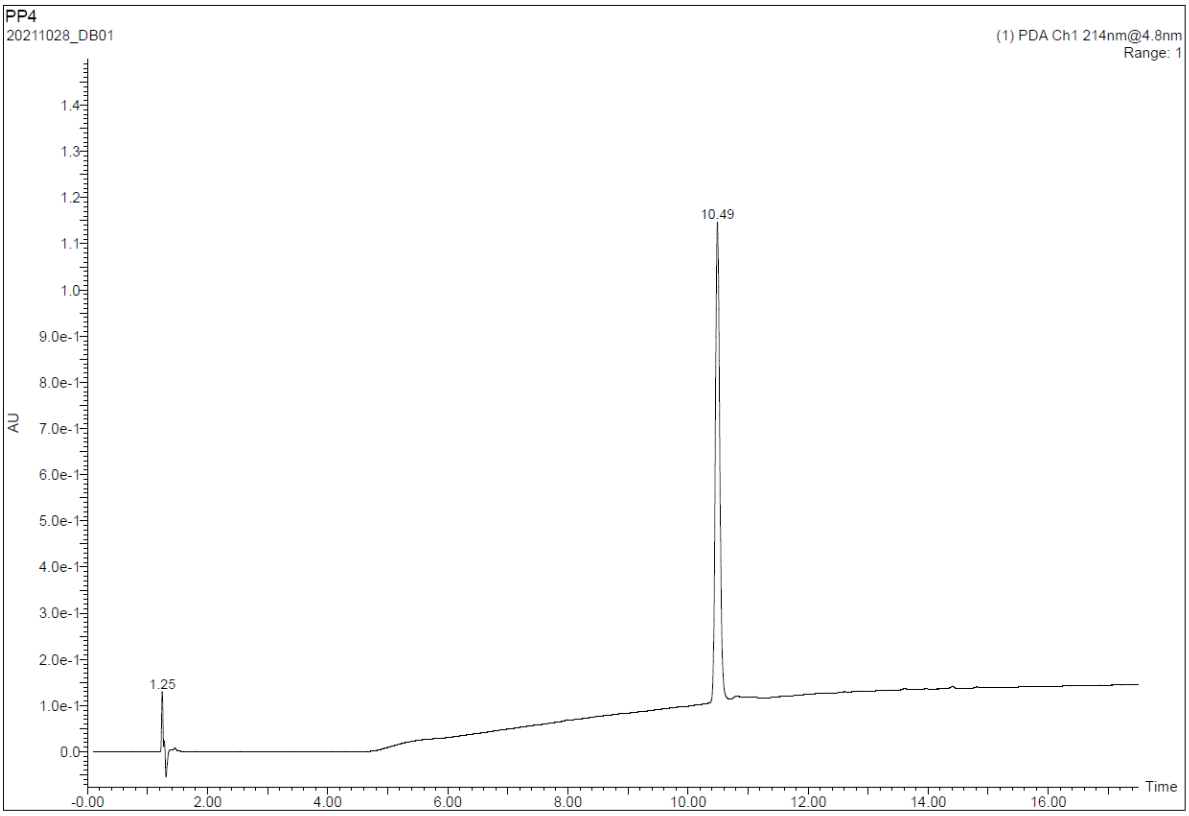

Figure S1 – UHPLC UV-Vis trace of peptide **P_4**, rt = 10.49 min, 214 nm

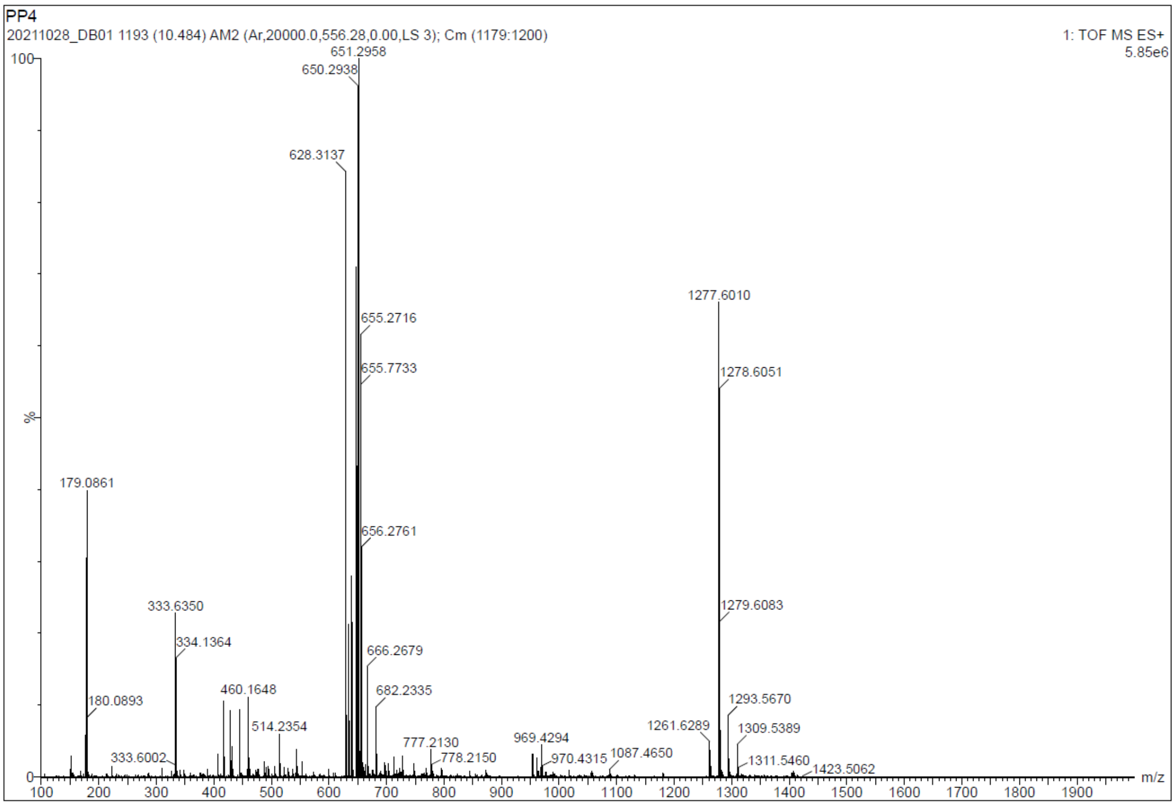

Figure S2 – Mass spectrum (100-2000 m/z) of peak at 10.49 min, **P_4** UHPLC

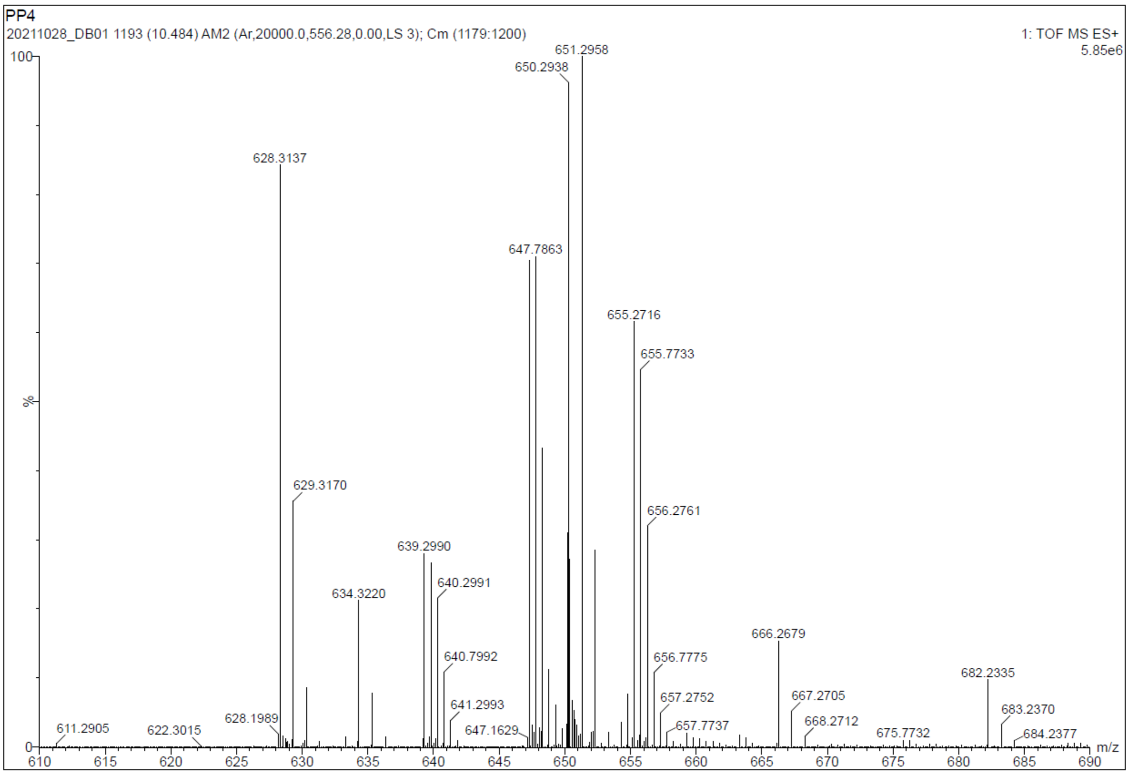

Figure S3 – Mass spectrum (610-690 m/z) of peak at 10.49 min, **P_4** UHPLC

SI 2.2.2 HRMS Spectra – HP_3

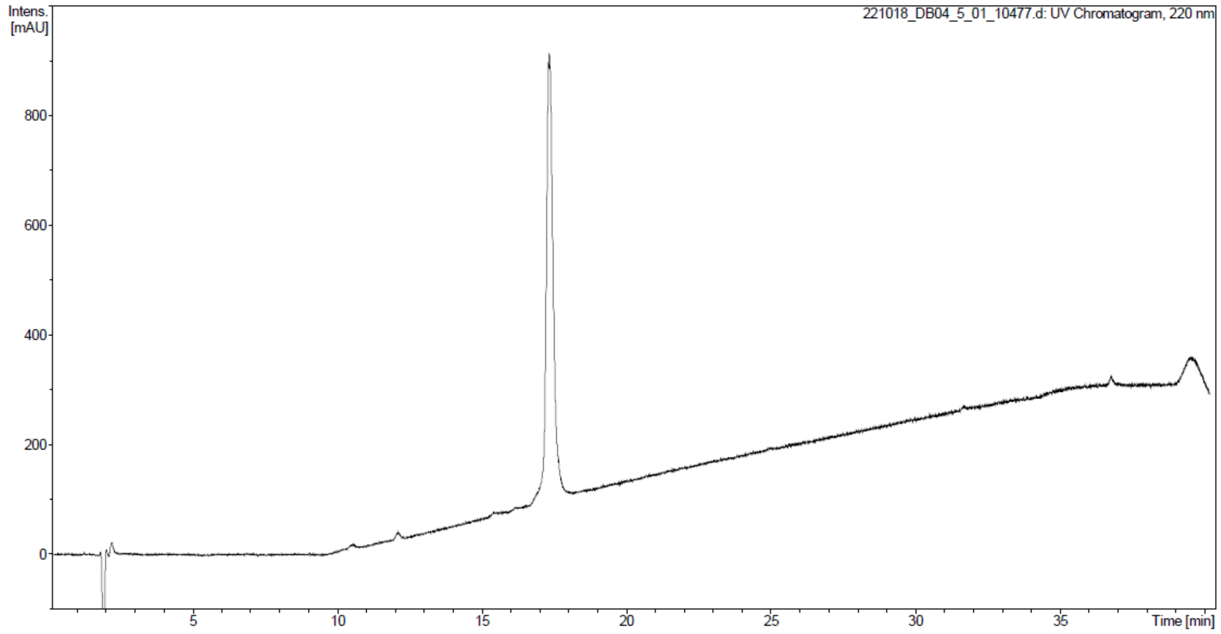

Figure S4 – HPLC UV-Vis trace of peptide **HP_3**, rt = 17.35 min, 220 nm

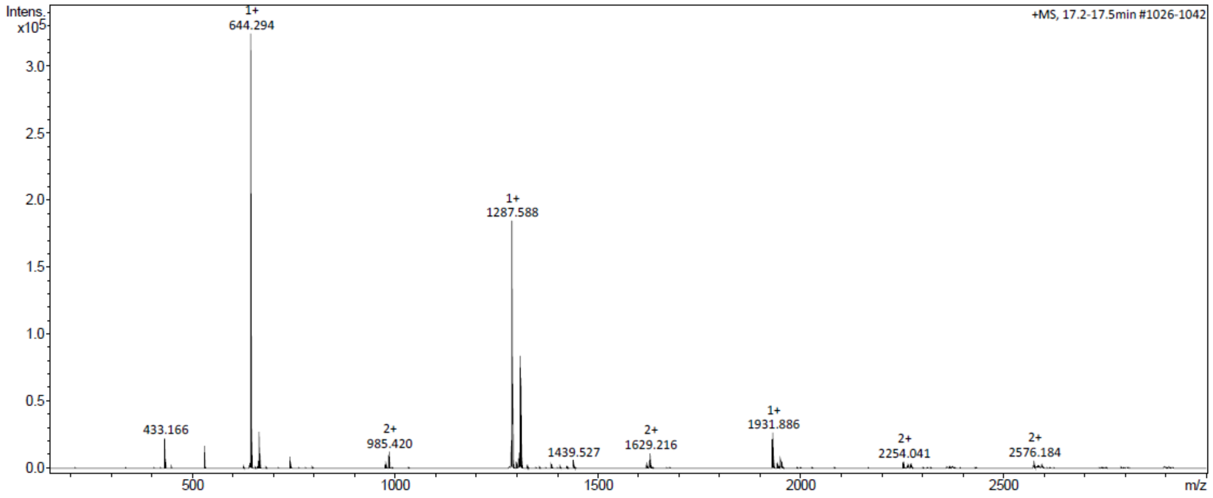

Figure S5 – Mass spectrum (100-2000 m/z) of peak at 17.35 min, **HP_3** HPLC

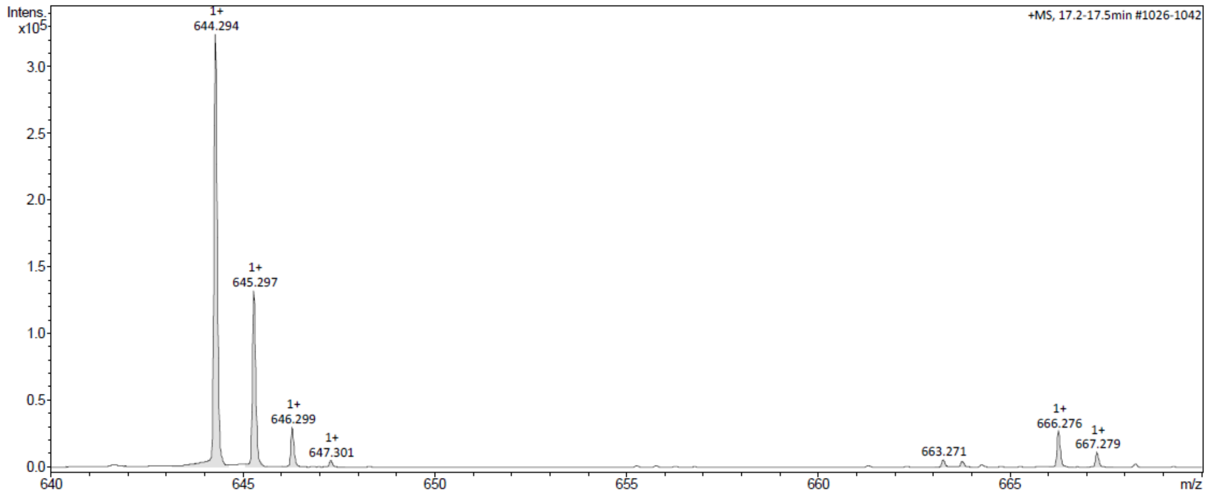

Figure S6 – Isotopic distribution spectrum of peak at 17.35 min (m/z 644.294), **HP₃** HPLC

SI 2.2.3 HRMS Spectra – PHP$_2$

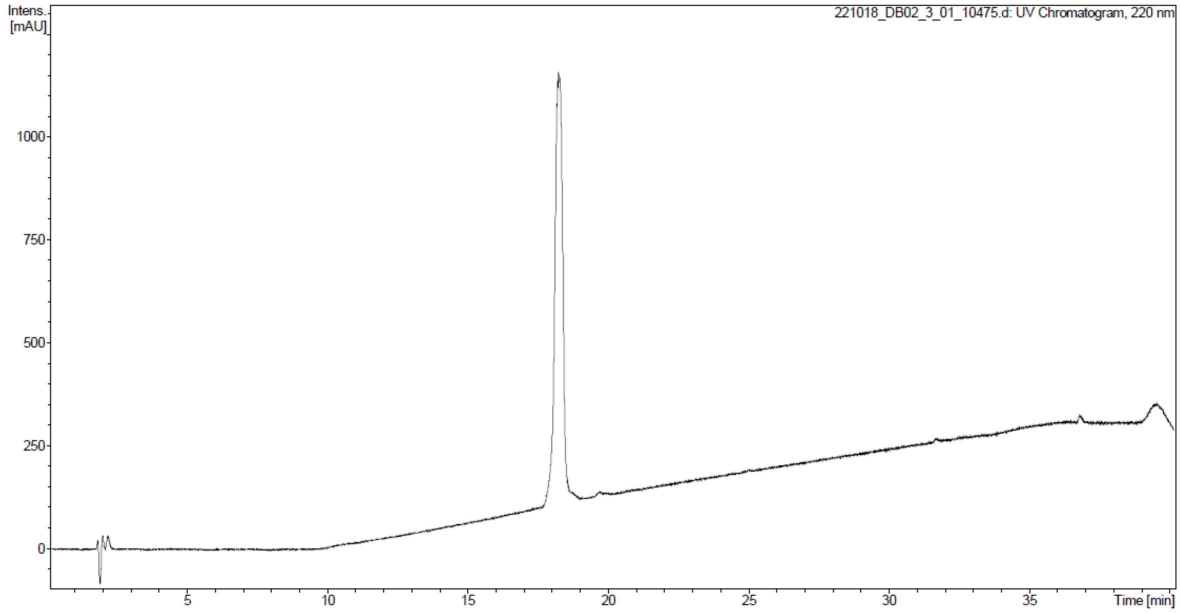

Figure S7 – HPLC UV-Vis trace of peptide **PHP$_2$**, rt = 18.25 min, 220 nm

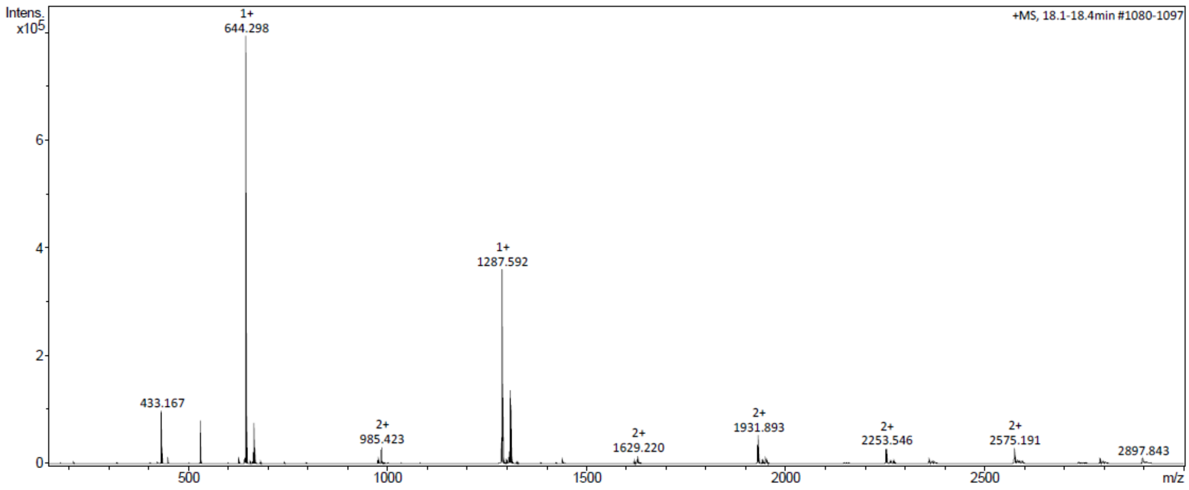

Figure S8 – Mass spectrum (100-2000 m/z) of peak at 18.25 min, **PHP$_2$** HPLC

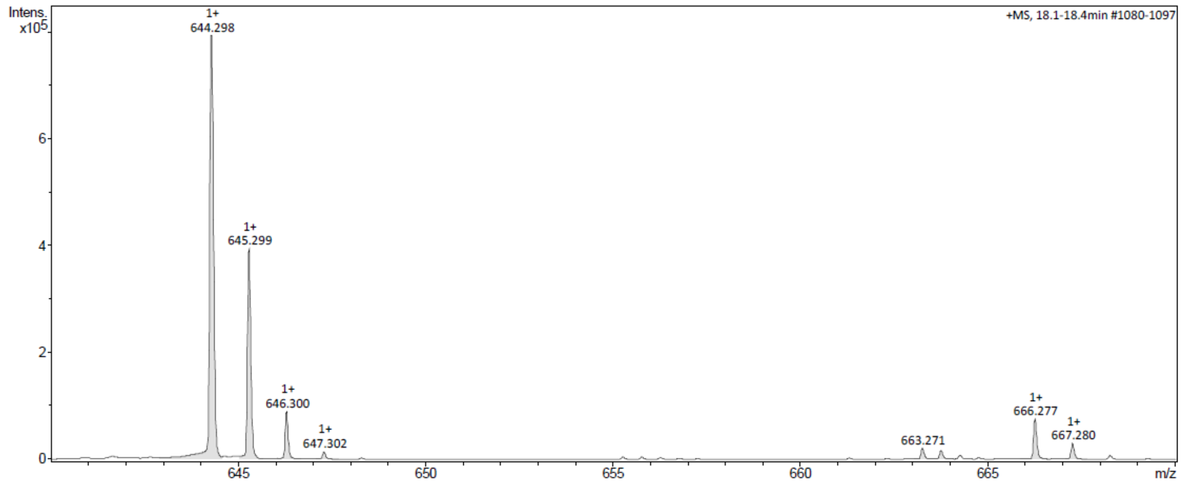

Figure S9 – Isotopic distribution spectrum of peak at 18.25 min (m/z 644.298), **PHP$_2$** HPLC

SI 2.2.4 HRMS Spectra – P_2HP

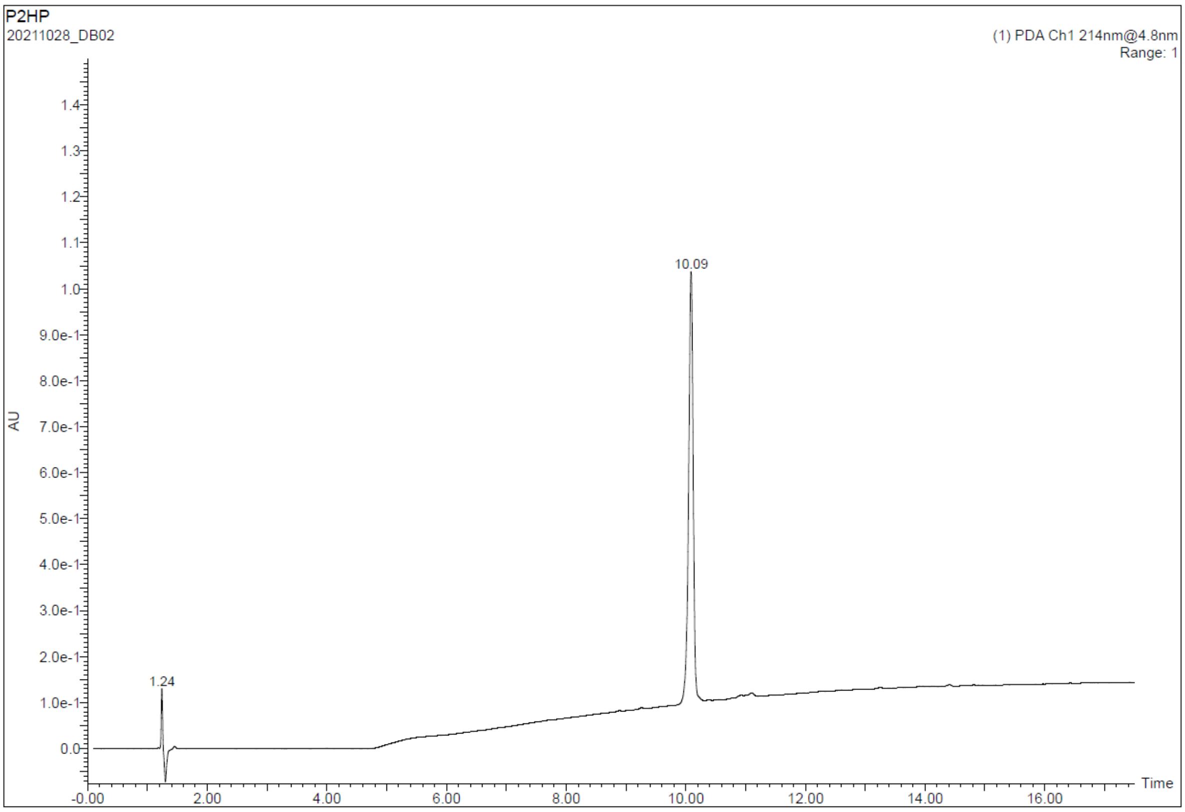

Figure S10 – UHPLC UV-Vis trace of peptide **P_2HP**, rt = 10.09 min, 214 nm

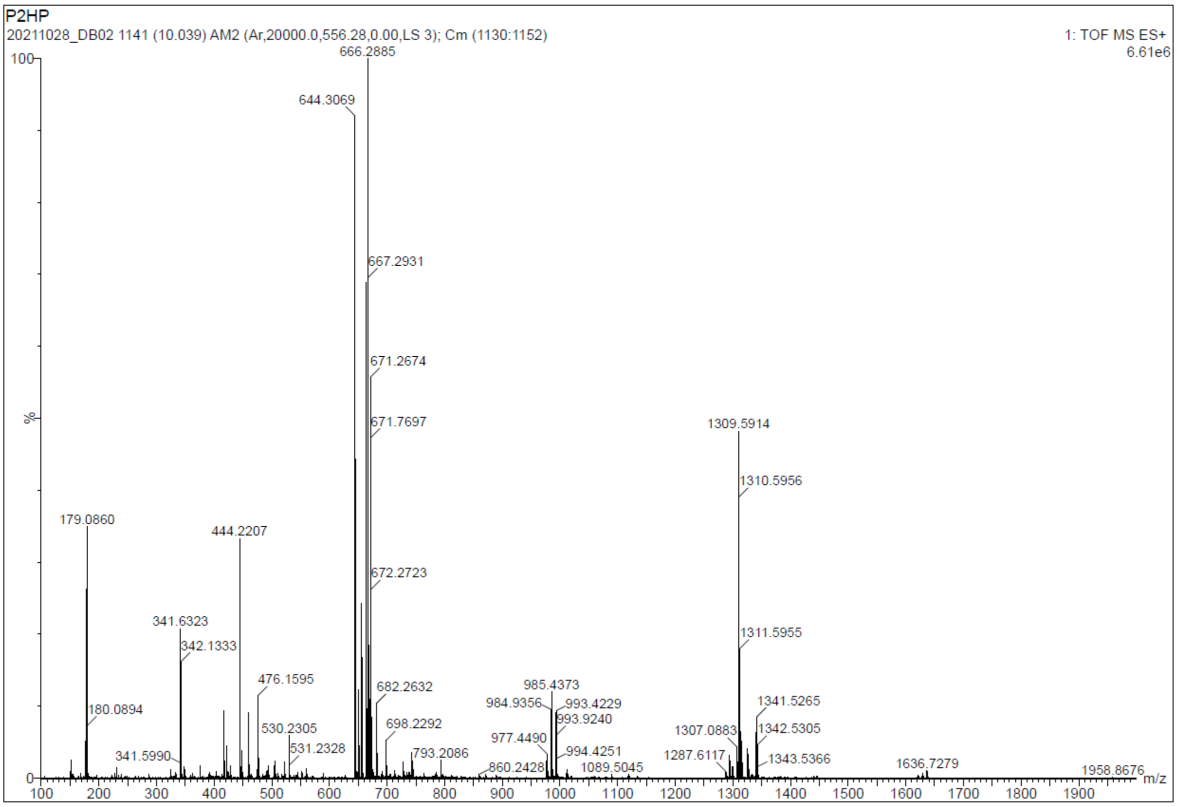

Figure S11 – Mass spectrum (100-2000 m/z) of peak at 10.09 min, **P_2HP** UHPLC

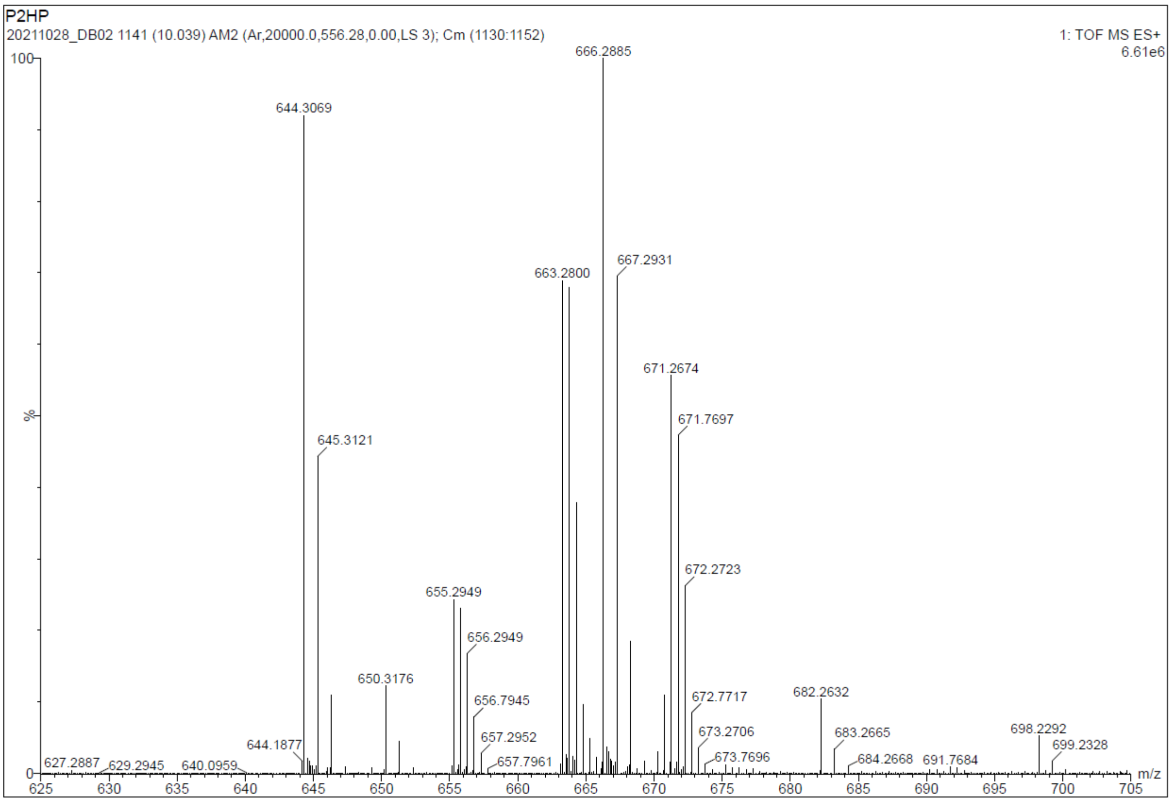

Figure S12 – Mass spectrum (625-705 m/z) of peak at 10.09 min, **P_2HP** UHPLC

SI 2.2.5 HRMS Spectra – P_3H

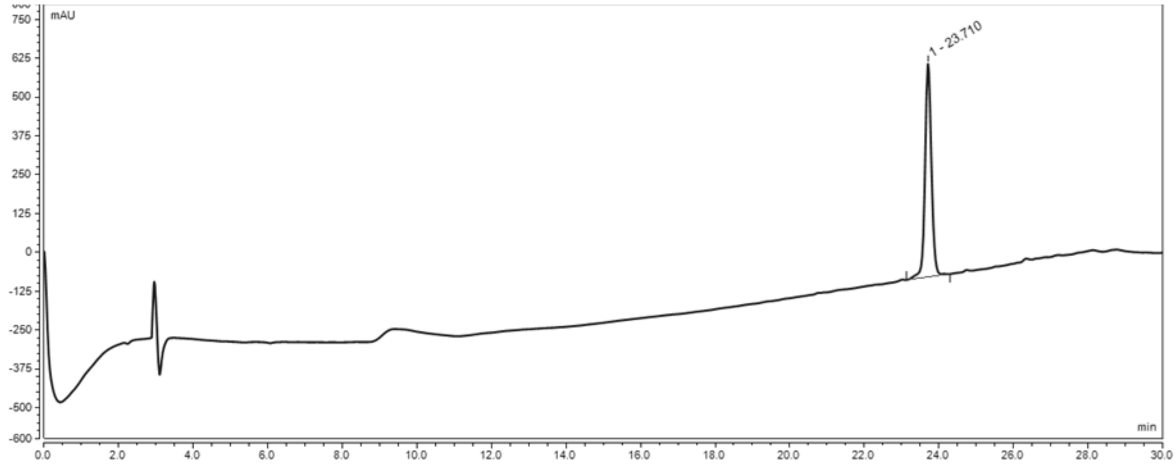

Figure S13 – HPLC UV-Vis trace of peptide **P_3H**, rt = 23.71 min, 225 nm

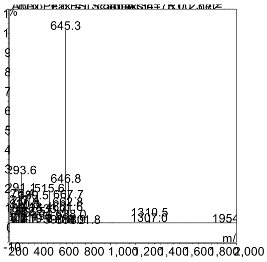

Figure S14 – Mass spectrum (200-2000 m/z) of peak at 23.71 min, **P_3H** HPLC

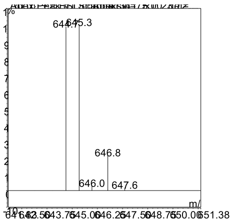

Figure S15 – Enlarged M+ ion region, Isotopic distribution of peak at 23.71 min (m/z 645.3), **P_3H**

SI 2.2.6 HRMS Spectra – HP_2H

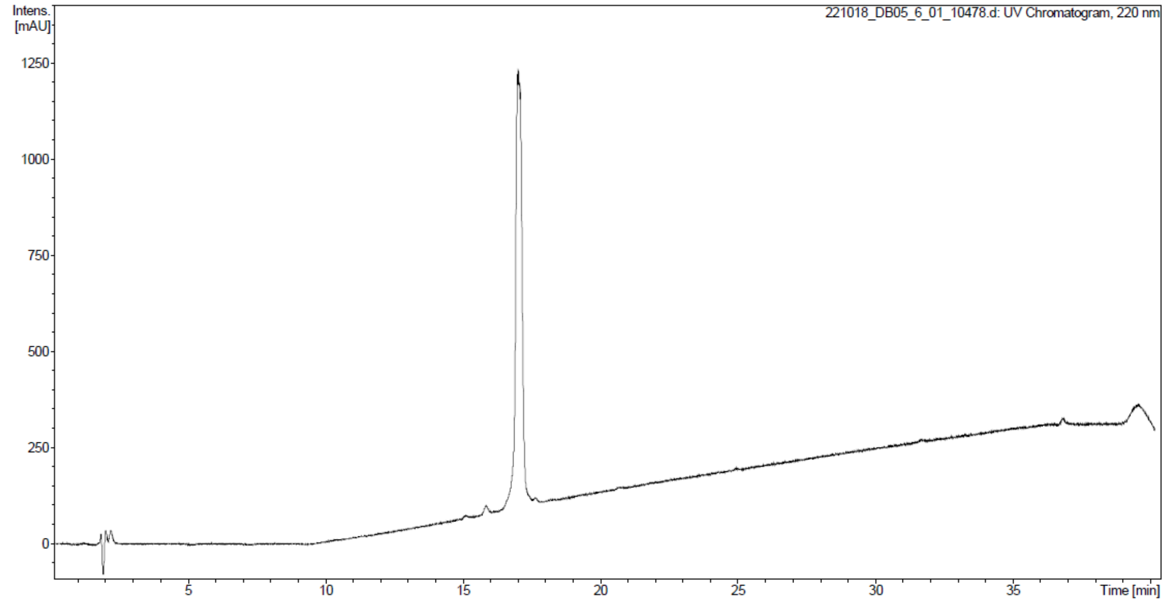

Figure S16 – HPLC UV-Vis trace of peptide **HP_2H**, rt = 17.05 min, 220 nm

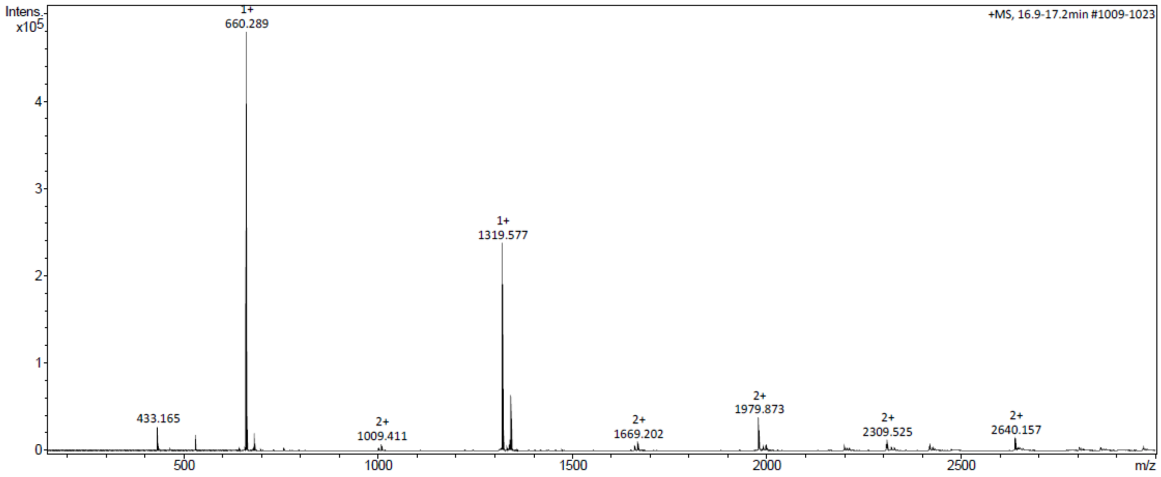

Figure S17 – Mass spectrum (100-2000 m/z) of peak at 17.05 min, **HP_2H** HPLC

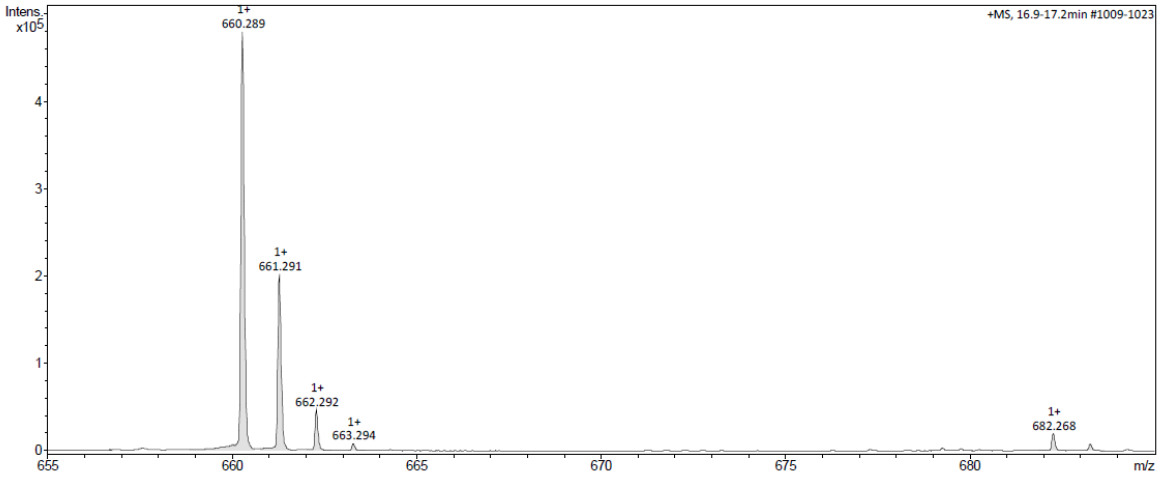

Figure S18 – Isotopic distribution spectrum of peak at 17.05 min (m/z 660.289), **HP_2H** HPLC

SI 2.2.7 MS Spectra – AcHP$_2$H

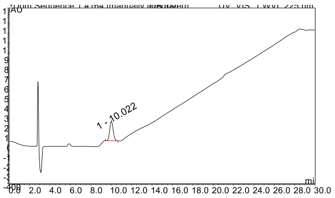

Figure S19 – HPLC UV-Vis trace of peptide **AcHP$_2$H**, rt = 10.022 min, 225 nm

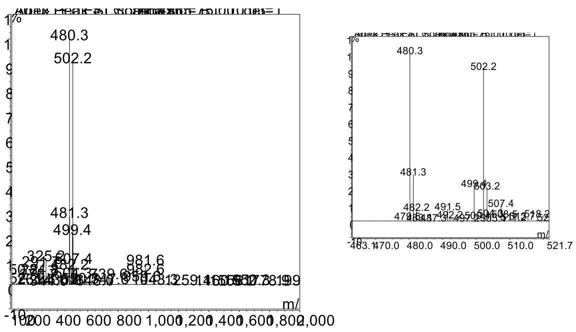

Figure S20 – Mass spectrum (100-2000 m/z) of peak at 10.022 min, **AcHP$_2$H** HPLC. M+ ion peak area enlarged.

SI 2.2.8 MS Spectra – Cis-HP_2H

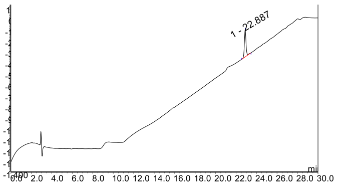

Figure S21 – HPLC UV-Vis trace of peptide ***Cis*-$\mathbf{HP_2H}$**, rt = 22.887 min, 225 nm

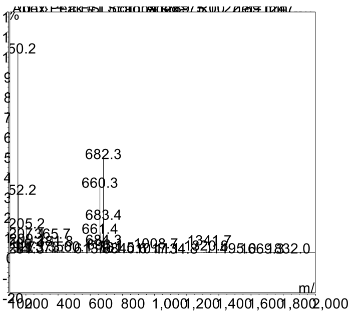

Figure S22 – Mass spectrum (100-2000 m/z) of peak at 22.887 min, ***Cis*-HP$_2$H** HPLC.

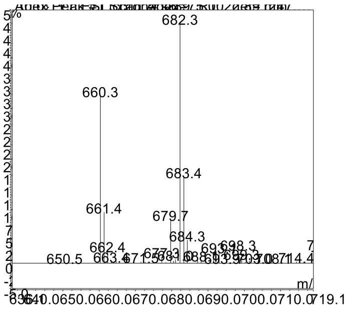

Figure S23 – M+ ion peak area enlarged region of MS, peak at 22.887 min, ***Cis***-**HP_2H**.

SI 2.2.9 MS Spectra – AcP_4

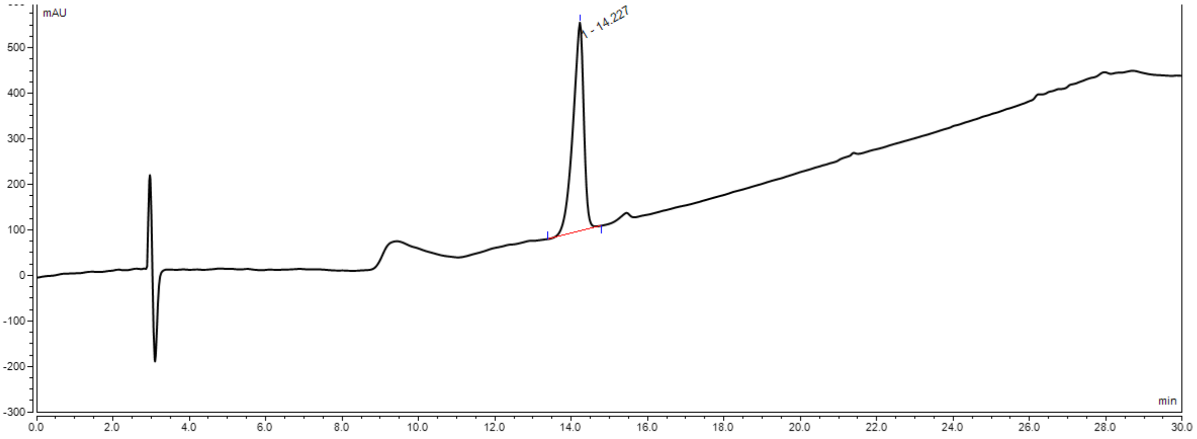

Figure S24 – HPLC UV-Vis trace of peptide **AcP_4**, rt = 14.227 min, 225 nm

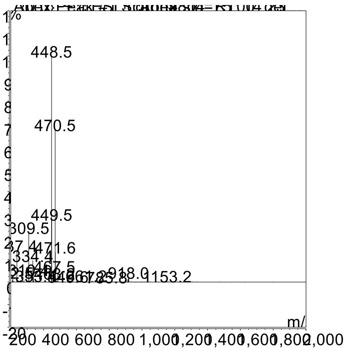

Figure S25 – Mass spectrum (200-2000 m/z) of peak at 14.23 min, **AcP$_4$**.

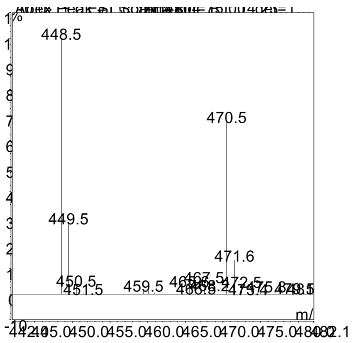

Figure S26 – M+ ion peak area enlarged region of MS, peak at 14.23 min, **AcP$_4$**.

SI 3. X-ray Diffraction data:

Crystallisation conditions:

Peptide **P_4** was fully dissolved (≈25-30 $mgml^{-1}$) in hot EtOH before slowly cooling the solution from 60+ °C overnight, forming crystals (colourless planks) in solution.[1]

Peptide **P_2HP** was crystallised by slowly cooling from a hot EtOH/EtOAc solution, forming crystals (colourless planks) in solution.

Peptides **HP_3**, **PHP_2**, **P_3H** and **HP_2H** were crystallised by slow evaporation from an EtOH/EtOAc solution, forming crystals (colourless planks). However, no crystals of **PHP_2** suitable for SCXRD analysis were found.

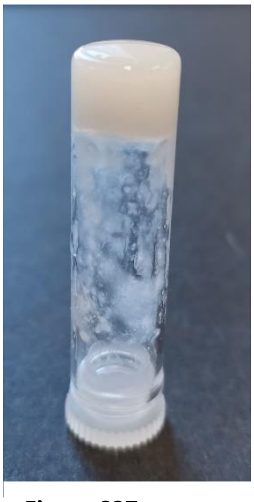

Figure S27 – Sample of peptide **$AcHP_2H$** in chloroform (6 mg ml^{-1}) after sonication.

Peptide **$AcHP_2H$** was dissolved in hot acetonitrile before slowly evaporating to form colourless needles. Sonication of a supersaturated solution instead forms an organogel, this occurred in acetonitrile, chloroform, and dichloromethane, with the strongest gelation in chloroform (*Figure S27*).

Crystallisation of peptide **AcP_4** was attempted in MeOH, EtOH, EtOH/EtOAc, ACN, and EtOH/ACN, producing a non-crystalline glassy solid or a viscous oil. The peptide crystallised readily from slow evaporation of a $CHCl_3$ solution to form colourless needles, however, the crystal quality was poor and unsuitable for single crystal analysis. The peptide was crystallised successfully from vapour diffusion of Et_2O into a solution of the peptide in $CHCl_3$ to produce colourless needles which were analysed via SCXRD analysis.

All crystalline samples were stable outside of their mother liquor. Crystals of peptide **$AcHP_2H$** melted under a stream of room temperature N_2 (290 K), likely due to the loss of ACN from the framework. Crystals of peptide **Cis-HP_2H**, degraded under a room temperature N_2 flow (300 K) also likely due to loss of encapsulated solvent within the framework pores.

SI 3.1 Single-crystal XRD data:

Single crystal XRD data for all peptides were collected on a Rigaku Oxford Diffraction SuperNova AS2 single crystal diffractometer using Cu Kα (λ = 1.54184) radiation, **$AcHP_2H$** and **AcP_4** used Mo Kα (λ = 0.71073). The crystals were mounted on a Mitegen micromount in Paratone immersion oil and temperature controlled using an Oxford Cryosystems 800-series Cryostream.

Using the software Olex2,[2] the structures were solved with the ShelXT structure solution program using intrinsic phasing and refined with the ShelXL refinement package using least squares minimization.[3–5]

CCDC-2127751,[1] 2234312, 2238152, 2238155, 2238160-1, 2238180, 2238252 and 2264145 contain the supplementary crystallographic data for this paper, including structure factors and refinement instructions, and can be obtained free of charge from The Cambridge Crystallographic Data Centre, 12 Union Road, Cambridge CB2 1EZ, UK (e-mail: depos-it@ccdc.cam.ac.uk), or via https://www.ccdc.cam.ac.uk/getstructures.

*Table S1 - Comparative table of crystallographic data for all crystal structures: **

Identification code	**PP_4-SPF**	**P2HP**	**P2HP-P4**	**P3H**	**HP3**	**HP2H**	**Cis-HP2H**	**AcHP2H**	**AcP4**
Empirical formula	$C_{37}H_{47}N_5O_7$	$C_{35}H_{45}N_5O_9$	$C_{36.5}H_{42.36}N_5O_{7.13}$	$C_{36}H_{41}N_5O_8$	$C_{35}H_{41}N_5O_7$	$C_{35}H_{40}N_5O_8$	$C_{35}H_{41}N_5O_8$	$C_{24}H_{36}N_6O_7$	$C_{24}H_{35}Cl_6N_5O_5$
Formula weight	673.79	679.76	665.19	671.74	643.73	658.72	659.73	520.59	686.27
Temperature /K	150(2)	150.15	290.0(2)	150.00(10)	150.15	295	150.15	294.0(5)	150.0(7)
Crystal system	monoclinic	monoclinic	monoclinic	monoclinic	monoclinic	monoclinic	orthorhombic	orthorhombic	monoclinic
Space group	$P2_1$	$P2_1$	$P2_1$	$P2_1$	C2	C2	$P2_12_12_1$	$P2_12_12_1$	$P2_1$
a/Å	16.4182(3)	16.6440(5)	16.6077(8)	16.8723(2)	22.1654(7)	22.476(2)	6.4160(2)	6.3773(10)	13.0167(8)
b/Å	6.27740(10)	6.3071(2)	6.3463(3)	6.45220(10)	6.3098(2)	6.2932(7)	12.0836(6)	18.232(4)	6.2940(6)
c/Å	18.2112(3)	17.8450(5)	18.3211(9)	32.1004(4)	23.0675(7)	23.245(3)	47.4708(17)	22.857(5)	19.6114(12)
α/°	90	90	90	90	90	90	90	90	90
β/°	109.152(2)	110.012(3)	109.933(5)	101.2140(10)	92.401(3)	92.036(10)	90	90	99.377(6)
γ/°	90	90	90	90	90	90	90	90	90
Volume/$Å^3$	1773.03(6)	1760.18(10)	1815.31(16)	3427.84(8)	3223.37(17)	3285.8(6)	3680.3(3)	2657.6(9)	1585.2(2)
Z	2	2	2	4	4	4	4	4	2
$\rho_{calc}g/cm^3$	1.262	1.283	1.217	1.302	1.326	1.332	1.191	1.301	1.438
μ/mm^{-1}	0.716	0.770	0.699	0.766	0.765	0.788	0.085	0.097	0.584
F(000)	720.0	724.0	707.0	1424.0	1368.0	1396.0	1400.0	1112.0	712.0
CCDC No.	2127750	2238161	2238180	2238152	2238155	2238160	2238252	2234312	2264145

**Peptides that form isostructural structures are highlighted in the same colour*

SI 3.1.1 P_4 SC-XRD:

X-ray diffraction data for peptide **P₄** is repeated where necessary from ref:(*Chem. – A Eur. J.*, 2022, **28**, e202202368),[1] for ease of comparison to other crystal structures, in both this section 3.1 and section 3.2, the collapsed structure after thermal activation is available from the above reference and CCDC-2156434. The crystal structure data was obtained from plank-shaped colourless crystals, crystallised by dissolving $\mathbf{P_4}$ in hot EtOH (60 °C, ≈25 mgml^{-1}) and allowing to slowly cool to room temperature forming crystals overnight within the solution. The crystals were stable outside of solution at room temperature showing no signs of deterioration over the timeframe of the experiment. To model the disordered solvent, the ethanol molecule was split into two components with a total occupancy of 1. The distances between the oxygen-carbon and carbon-carbon atoms were set to the expected values of 1.43 Å and 1.51 Å respectively. Upon refinement, these distances were then fixed to the same value between the two components and the anisotropic displacement parameters were set to be equivalent within the molecule.

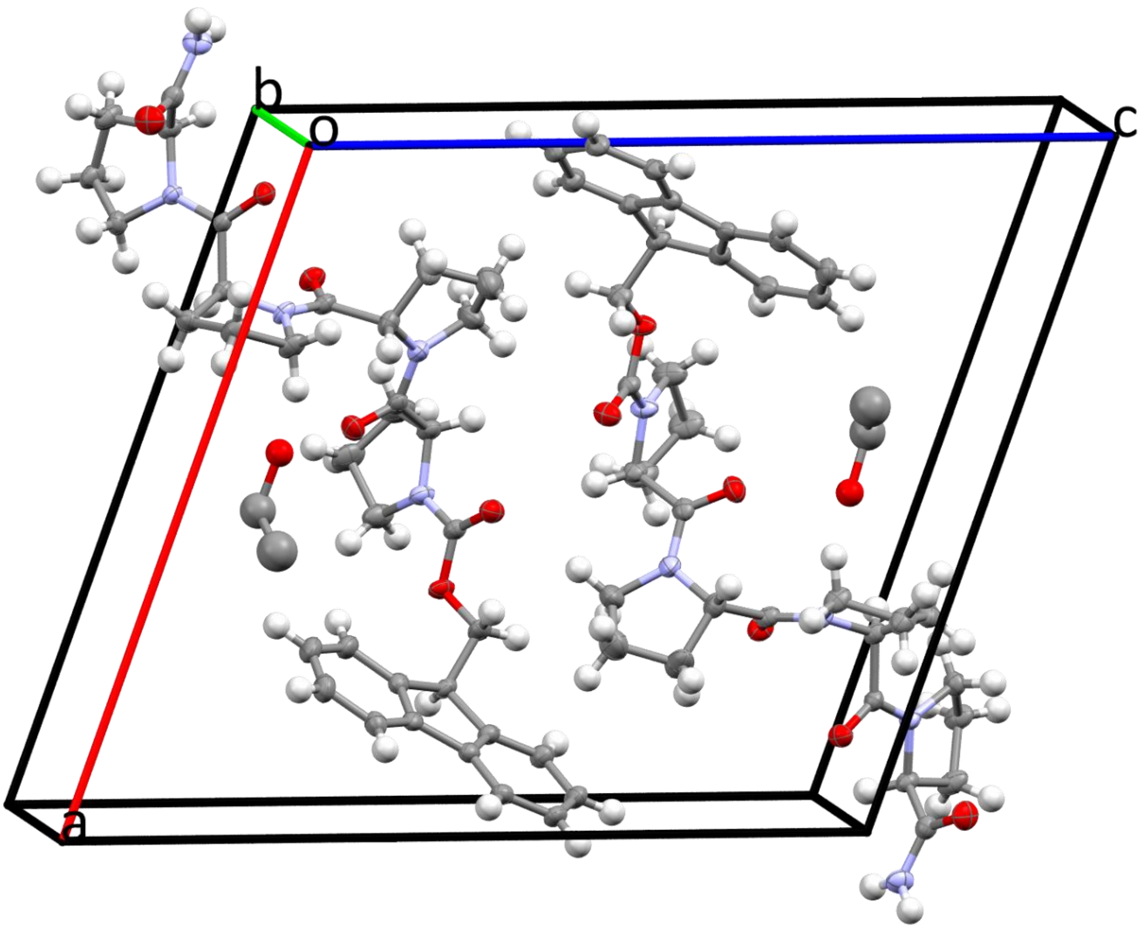

Figure S28 – Crystal structure of peptide P_4 showing the unit cell, all molecules whose centroids fit are shown, atomic displacement parameters are shown at 50 % probability . .

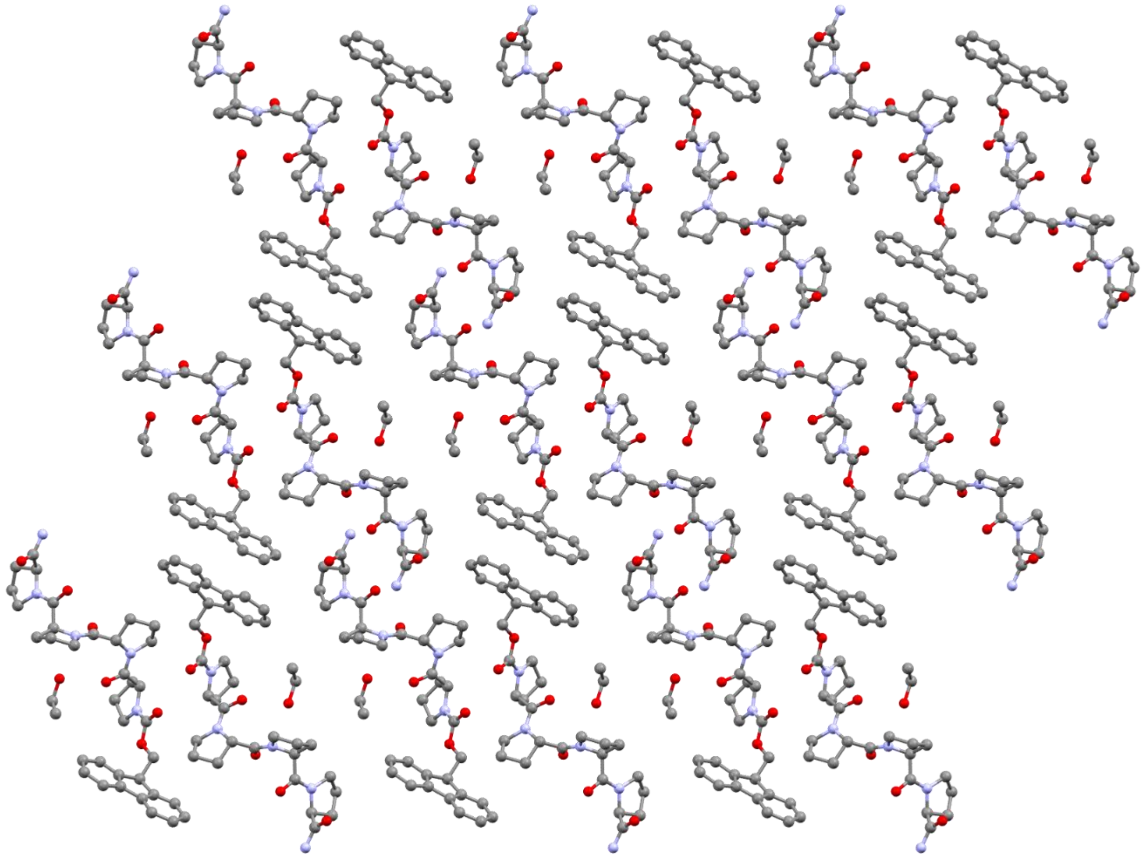

Figure S29 – Crystal structure of peptide P_4 showing the packed extended structure (3x3x3), view along the *b* axis, hydrogens not shown, atomic displacement parameters are shown at 50 % probability .

P_4 (PP4-SPF)[1]was slowly cooled from 290 K to 150 K with a ramp rate of 280 K/h

Identification code	**PP_4-SPF**
Empirical formula	$C_{37}H_{47}N_5O_7$
Formula weight	673.79
Temperature/K	150(2)
Crystal system	monoclinic
Space group	$P2_1$
a/Å	16.4182(3)
b/Å	6.27740(10)
c/Å	18.2112(3)
α/°	90
β/°	109.152(2)
γ/°	90
Volume/Å^3	1773.03(6)
Z	2
ρ_{calc}g/cm^3	1.262
μ/mm^{-1}	0.716
F(000)	720.0
Crystal size/mm^3	0.217 × 0.063 × 0.054

Radiation	Cu Kα (λ = 1.54184)
2Θ range for data collection/°	8.84 to 146.496
Index ranges	$-16 \le h \le 20$, $-7 \le k \le 7$, $-22 \le l \le 19$
Reflections collected	20137
Independent reflections	6927 [R_{int} = 0.0225, R_{sigma} = 0.0217]
Data/restraints/parameters	6927/8/444
Goodness-of-fit on F^2	1.040
Final R indexes [I>=2σ (I)]	R_1 = 0.0383, wR_2 = 0.1041
Final R indexes [all data]	R_1 = 0.0397, wR_2 = 0.1055
Largest diff. Peak/hole / e Å^{-3}	0.56/-0.55
Flack parameter	0.01(5)
CCDC No.	2127750

SI 3.1.2 HP_3 SC-XRD:

The crystal structure data was obtained from colourless plank crystals, the crystal quality was relatively poor, the crystal was mounted on a Mitegen micromount in Paratone immersion oil and cooled to 150 K using an Oxford Cryosystems 800-series Cryostream. The crystal structure was obtained with some regions of significant disorder. The linker to the fluorenyl group was significantly disordered as such the atom C13-14 were split and EADP applied. Split SAME was also carried out on O2. The Pro1 pyrrolidine ring was disordered, as such C16-19 were split and EADP applied, split SAME was applied to O8. The pro3 ring was also disordered as such C26-28 were split and DELU applied. Only small voids are present (40.80 Å^3, 1.3 %-unit cell volume, Probe radius 1.2 Å, grid spacing 0.5 Å) within the structure that do not extend through the framework, forming a non-porous structure. The fmoc region is disordered and this extends to the neighbouring peptide's Pro3 sidechain, while the C-terminus is well ordered.

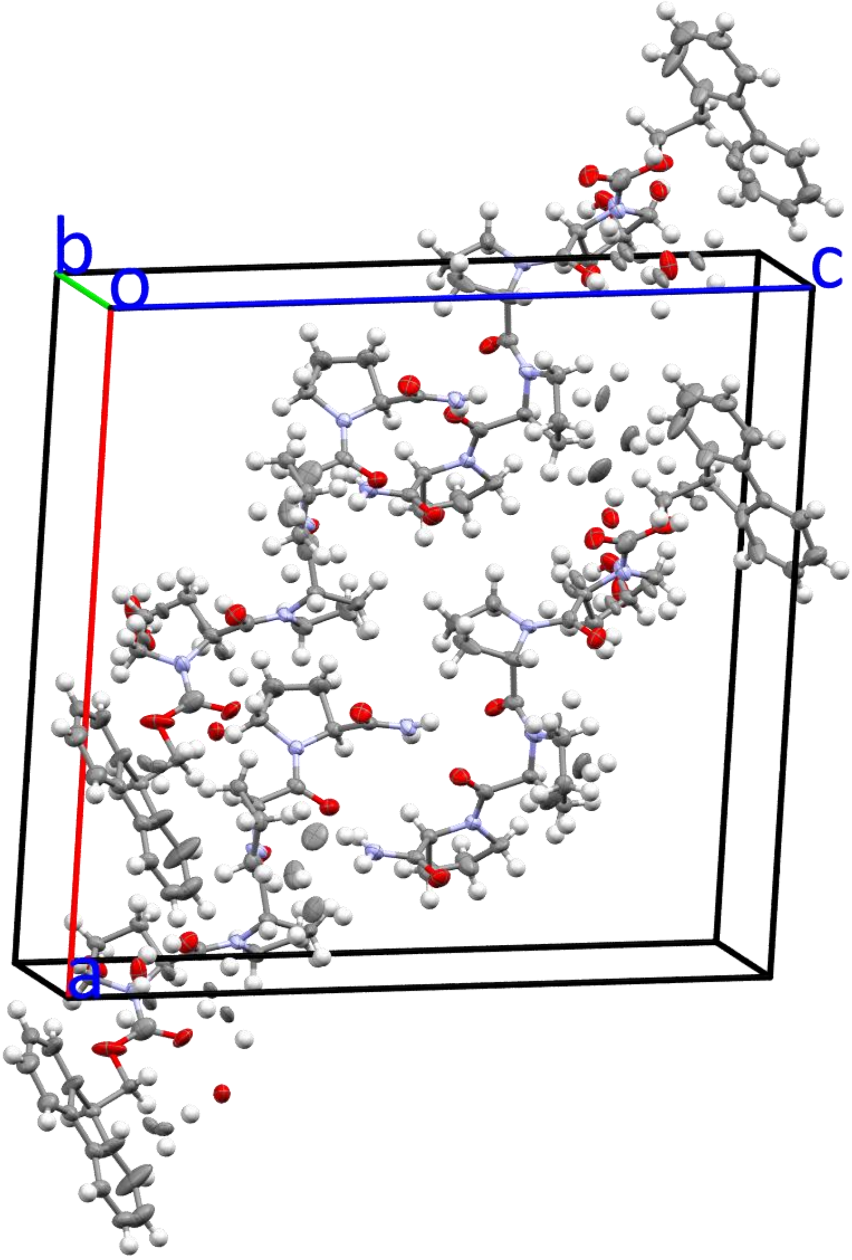

Figure S30 – Crystal structure of peptide **HP$_3$** showing the unit cell, Atomic displacement parameters are shown at 50 % probability .

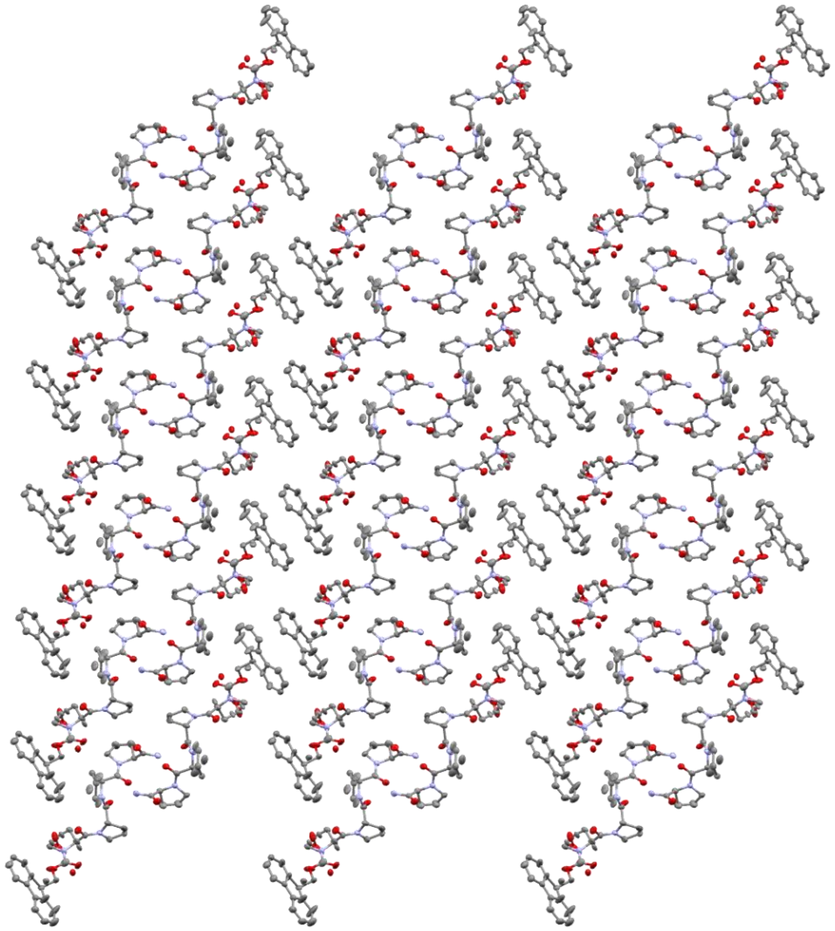

Figure S31 – Crystal structure of peptide **HP$_3$** showing the packed extended structure (3x3x3), view along the *b* axis, hydrogens not shown, atomic displacement parameters are shown at 50 % probability.

**Crystal data and structure refinement for HP$_3$**	
Identification code	HP3
Empirical formula	$C_{35}H_{41}N_5O_7$
Formula weight	643.73
Temperature/K	150.15
Crystal system	monoclinic
Space group	C2
a/Å	22.1654(7)
b/Å	6.3098(2)
c/Å	23.0675(7)
α/°	90
β/°	92.401(3)
γ/°	90
Volume/Å^3	3223.37(17)
Z	4
ρ_{calc}g/cm^3	1.326
μ/mm^{-1}	0.765
F(000)	1368.0
Crystal size/mm^3	0.21 × 0.07 × 0.06

Radiation	CuKα (λ = 1.54184)
2Θ range for data collection/°	7.672 to 136.628
Index ranges	-26 ≤ h ≤ 26, -7 ≤ k ≤ 7, -27 ≤ l ≤ 27
Reflections collected	11914
Independent reflections	5885 [R_{int} = 0.0822, R_{sigma} = 0.0962]
Data/restraints/parameters	5885/22/493
Goodness-of-fit on F^2	1.096
Final R indexes [I>=2σ (I)]	R_1 = 0.0883, wR_2 = 0.2371
Final R indexes [all data]	R_1 = 0.1103, wR_2 = 0.2675
Largest diff. peak/hole / e Å^{-3}	0.35/-0.38
Flack parameter	-0.5(5)
CCDC No.	2238155

SI 3.1.3 PHP$_2$ SC-XRD:

Crystallised from slow-evaporation of a propan-1-ol solution forming long fibrous crystals, however these were not suitable for SC-XRD analysis. Crystallised poorly from an ethanol/ethyl acetate solution and methanol/water solution. However, poor quality powder diffraction data was obtained, see SI 3.2.

Analysing the other positions on the **P$_4$** backbone, the Pro2 residue on the **P$_4$** backbone is aligned with adjacent peptides' aromatic groups and the closest hydrogen bond acceptor (**H$_A$**) is 4.5 Å from the C4 position, which suggests the current crystal structure is unlikely to satisfy the hydrogen bond donation of a new hydroxyl at this position (SI 3.1.3, *Figure* S32).

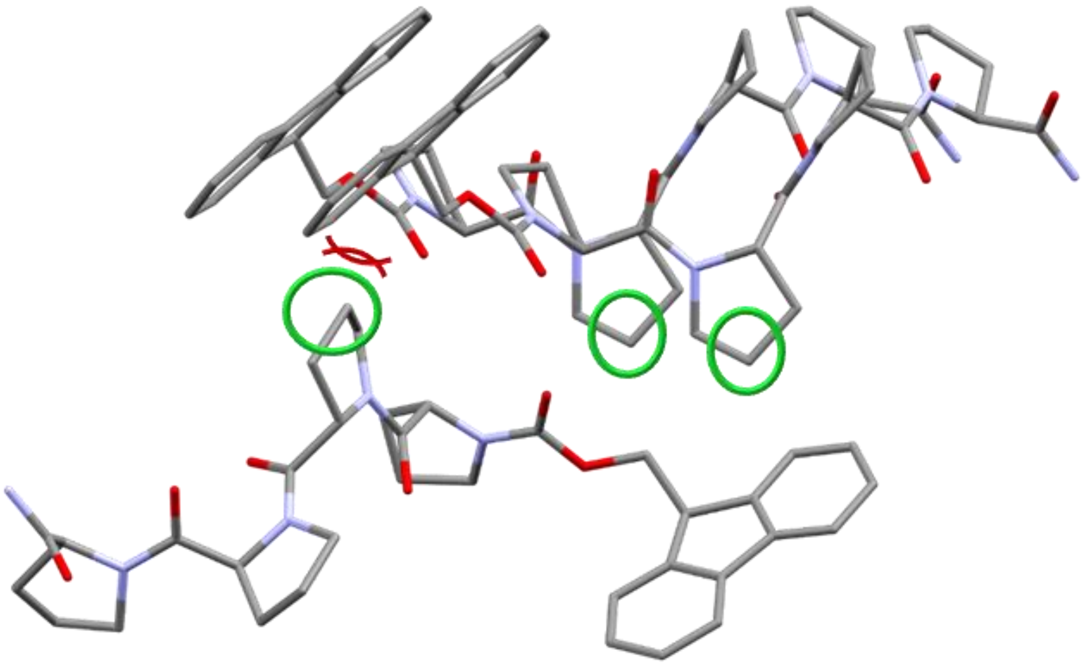

Figure S32 – Crystal structure of **P$_4$**, highlighting the C4 positions on the Pro2 residue (green), the location of the new hydroxy moiety in the **PHP$_2$** peptide, and the lack of close contact hydrogen bond acceptors.

SI 3.1.4 P$_2$HP SC-XRD:

The crystal structure data was obtained from colourless plank crystals, the crystal was mounted on a Mitegen micromount in Paratone immersion oil and cooled to 150 K using an Oxford Cryosystems 800-series Cryostream. **P_2HP** crystal structure was isostructural to **P_4**, and two water molecules were modelled within the voids of the framework. One of the water molecules was disordered as such split SAME was applied to O9. EADP was applied O2-C15. This reduced pore size is too small to accommodate EtOH molecules, as such the framework is selective towards H_2O molecules in the wet solvent during crystallisation, which can be clearly modelled in the crystal structure (*Figure S33*).

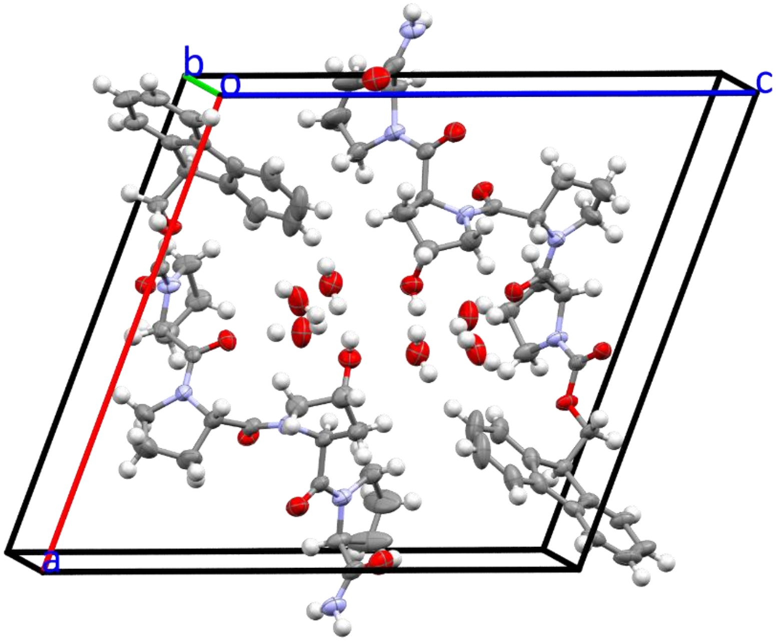

Figure S33 – Crystal structure of peptide P_2HP showing the unit cell, atomic displacement parameters are shown at 50 % probability .

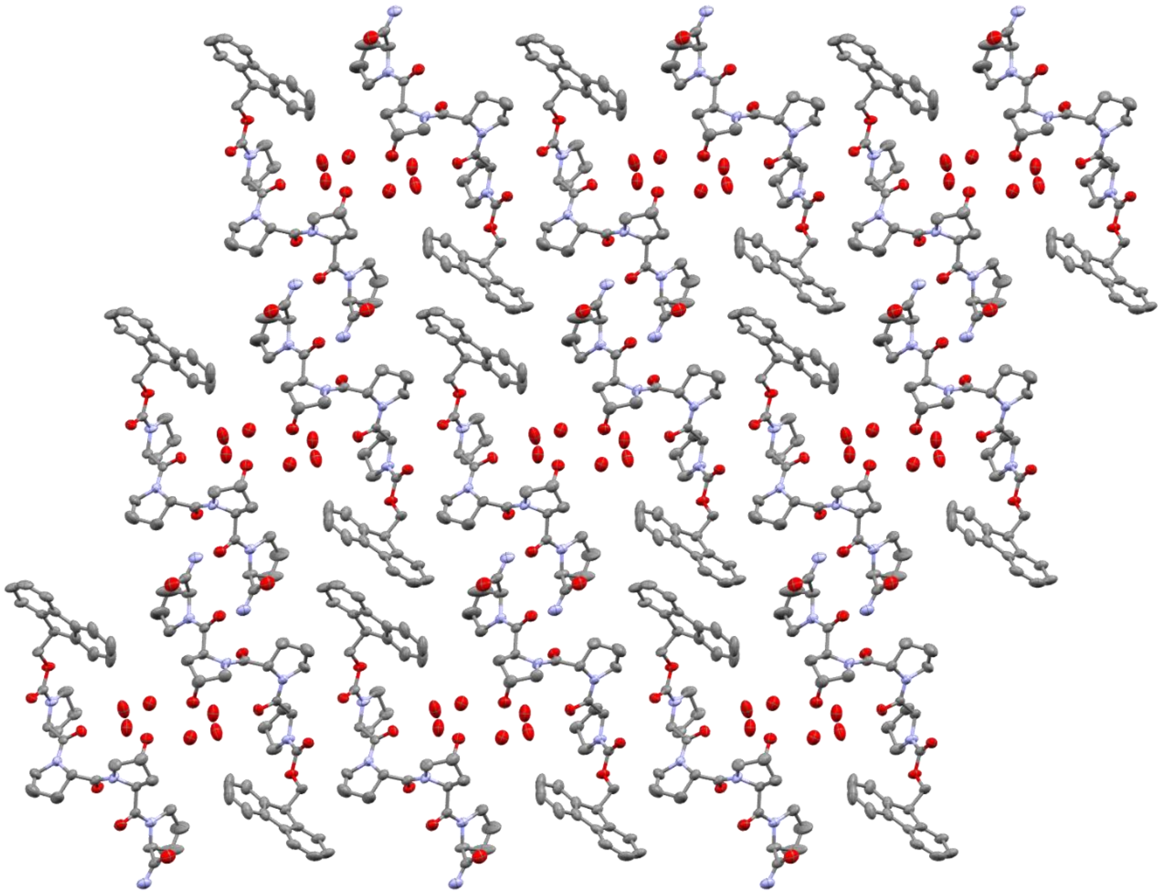

Figure S34 – Crystal structure of peptide P_2HP showing the packed extended structure (3x3x3), view along the *b* axis, hydrogens not shown, atomic displacement parameters are shown at 50 % probability

**Crystal data and structure refinement for P_2HP**	
Identification code	P2HP
Empirical formula	$C_{35}H_{45}N_5O_9$
Formula weight	679.76
Temperature/K	150.15
Crystal system	monoclinic
Space group	$P2_1$
a/Å	16.6440(5)
b/Å	6.3071(2)
c/Å	17.8450(5)
α/°	90
β/°	110.012(3)
γ/°	90
Volume/Å^3	1760.18(10)
Z	2
ρ_{calc}g/cm^3	1.283
μ/mm^{-1}	0.770
F(000)	724.0
Crystal size/mm^3	0.12 × 0.07 × 0.06
Radiation	CuKα (λ = 1.54184)
2Θ range for data collection/°	8.956 to 136.434

Index ranges	-20 ≤ h ≤ 20, -7 ≤ k ≤ 7, -15 ≤ l ≤ 21
Reflections collected	18801
Independent reflections	6417 [R_{int} = 0.0425, R_{sigma} = 0.0447]
Data/restraints/parameters	6417/1/470
Goodness-of-fit on F^2	1.051
Final R indexes [I>=2σ (I)]	R_1 = 0.0515, wR_2 = 0.1389
Final R indexes [all data]	R_1 = 0.0621, wR_2 = 0.1473
Largest diff. peak/hole / e Å^{-3}	0.35/-0.29
Flack parameter	-0.10(10)
CCDC No.	2238161

SI 3.1.5 P_3H SC-XRD:

The crystal structure data was obtained from colourless plank crystals, the crystal was mounted on a Mitegen micromount in Paratone immersion oil and cooled to 150 K using an Oxford Cryosystems 800-series Cryostream. The structure was resolved with two peptides comprising the asymmetric unit (Z' = 2), joined by the typical C-terminal amide NH_2 hydrogen bonds. Each of these peptides was modelled with a disordered EtOH molecules within the pores with partial occupancies (0.5). The oxygen atoms of each were disordered as such split SAME was applied to each (O8-O8X). While a model of the atomic positions of this disordered solvent is included it is likely that an atomistic model is not fully appropriate for the disordered electron density inside the pore.

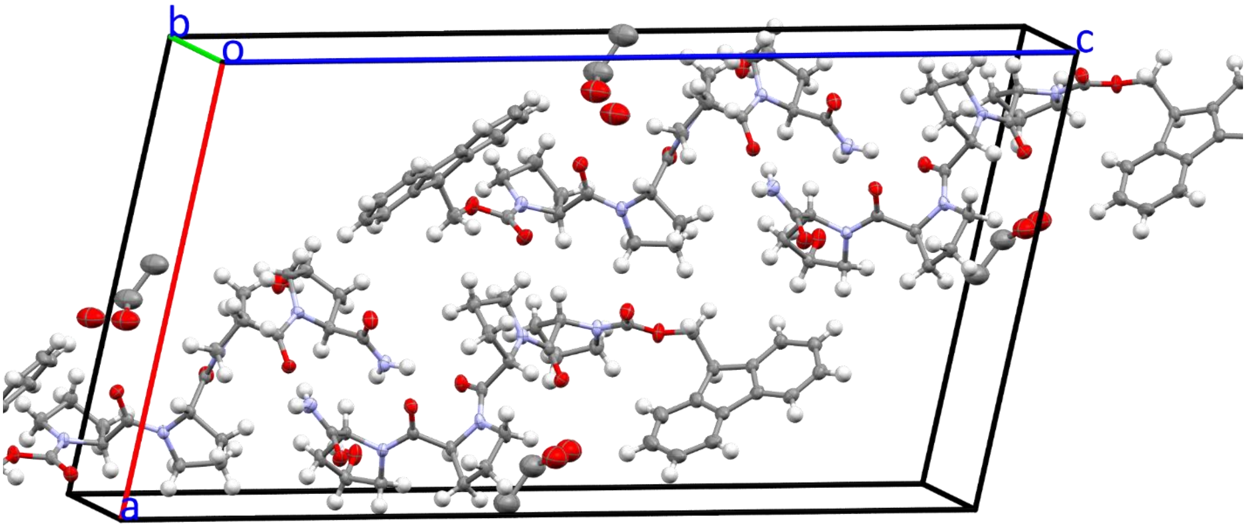

Figure S35 – Crystal structure of peptide P_3H showing the unit cell, Atomic displacement parameters are shown at 50 % probability

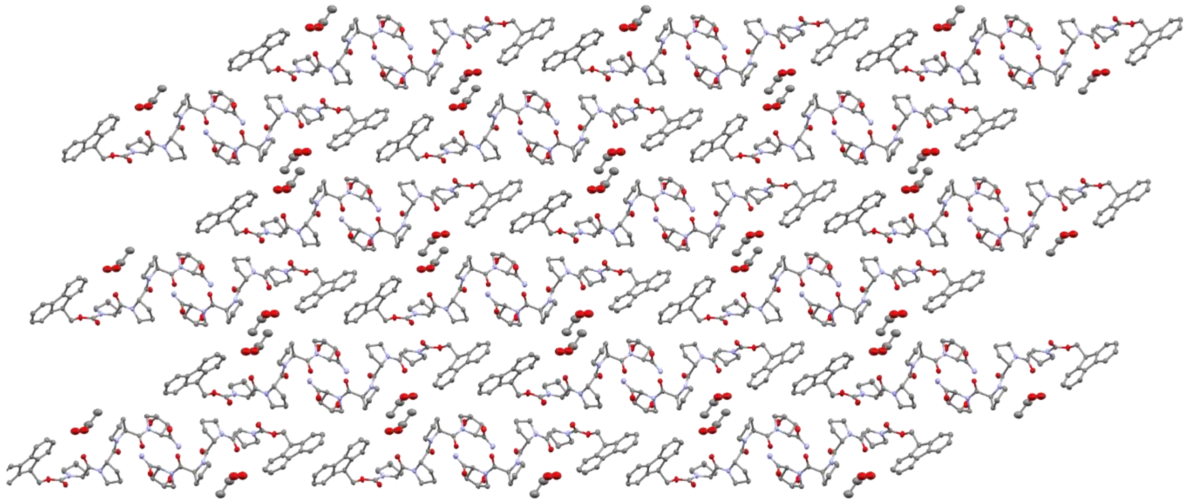

Figure S36 – Crystal structure of peptide P3H showing the packed extended structure (3x3x3), view along the *b* axis, hydrogens not shown, atomic displacement parameters are shown at 50 % probability

**Crystal data and structure refinement for P_3H**	
Identification code	P3H
Empirical formula	$C_{36}H_{41}N_5O_8$
Formula weight	671.74
Temperature/K	150.00(10)
Crystal system	monoclinic
Space group	$P2_1$
a/Å	16.8723(2)
b/Å	6.45220(10)
c/Å	32.1004(4)
α/°	90
β/°	101.2140(10)
γ/°	90
Volume/Å^3	3427.84(8)
Z	4
ρ_{calc}g/cm^3	1.302
μ/mm^{-1}	0.766
F(000)	1424.0
Crystal size/mm^3	0.16 × 0.11 × 0.07
Radiation	CuKα (λ = 1.54184)
2Θ range for data collection/°	6.956 to 144.164
Index ranges	-18 ≤ h ≤ 20, -7 ≤ k ≤ 7, -39 ≤ l ≤ 39
Reflections collected	38516
Independent reflections	13166 [R_{int} = 0.0224, R_{sigma} = 0.0216]
Data/restraints/parameters	13166/51/909
Goodness-of-fit on F^2	1.043
Final R indexes [I>=2σ (I)]	R_1 = 0.0416, wR_2 = 0.1251
Final R indexes [all data]	R_1 = 0.0431, wR_2 = 0.1274
Largest diff. peak/hole / e Å^{-3}	1.24/-0.86
Flack parameter	0.07(4)
CCDC No.	2238152

SI 3.1.6 P_4-P_2HP SC-XRD:

The crystal structure data was obtained from colourless plank crystals crystallised via slow cooling of a hot supersaturated ethanol solution of peptides $\mathbf{P_4}$ and $\mathbf{P_2}$**HP** in equimolar concentrations. The crystal was mounted on a Mitegen micromount in Paratone immersion oil and kept at 290 K using an Oxford Cryosystems 800-series Cryostream. The mixed peptides crystal structure was isostructural to $\mathbf{P_2}$**HP** and $\mathbf{P_4}$. Disordered EtOH molecules were modelled in the pores with a partial occupancy of 0.75. Split same was applied to the EtOH atoms C36-37. H atoms were not assigned to the EtOH molecules due to the high level of disorder. Interestingly this framework adopted the *endo* conformation similarly to $\mathbf{P_4}$ alone, having significantly less impact on the channel volume (Volume 233.02 Å^3, 12.8 % / unit cell, Probe r = 1.2 Å, Grid spacing 0.4 Å), and thus contained EtOH within the pores rather than being selective towards H_2O (*Figure S37 and S38*).

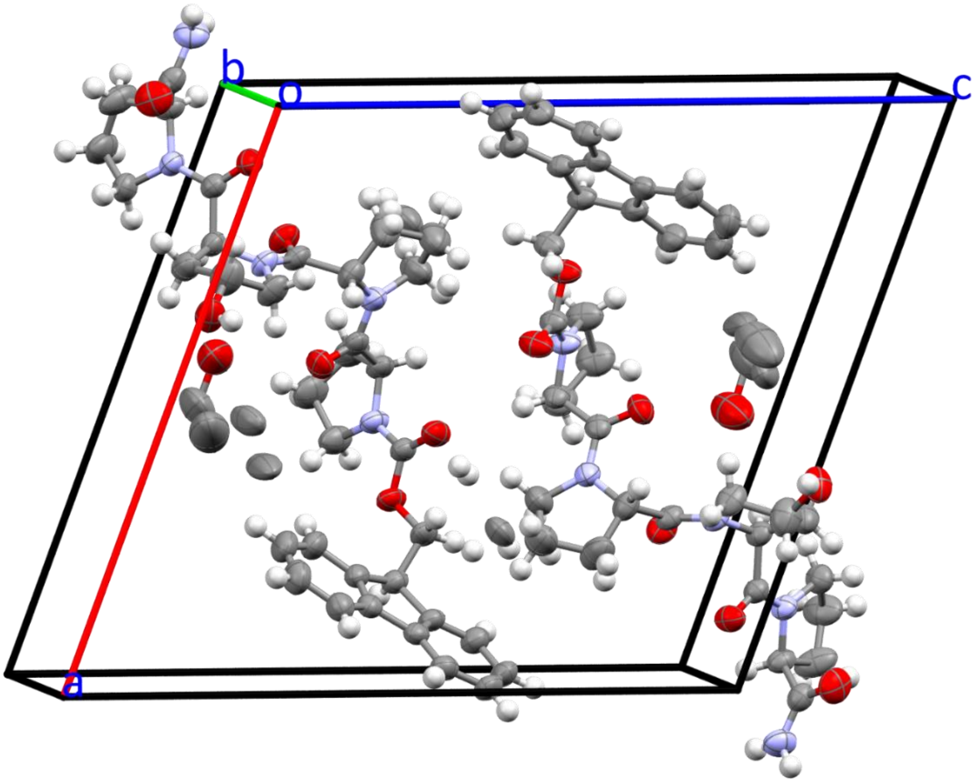

Figure S37 – Crystal structure of peptide P_4+P_2HP showing the unit cell, atomic displacement parameters are shown at 50 % probability .

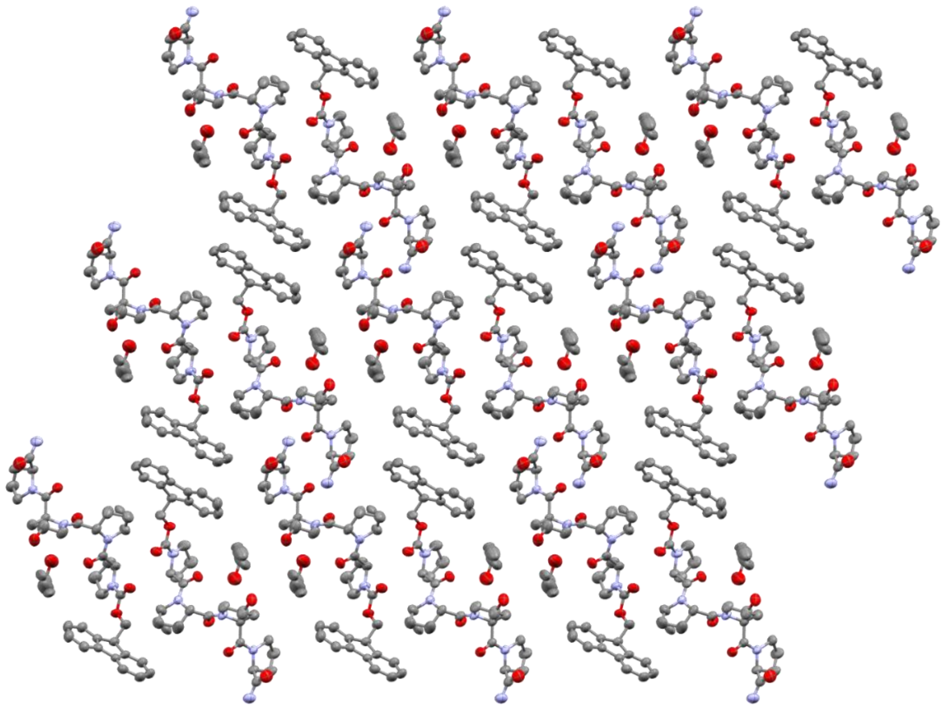

Figure S38 – Crystal structure of peptide P_4+P_2HP showing the packed extended structure (3x3x3), view along the *b* axis, hydrogens not shown, atomic displacement parameters are shown at 50 % probability .

**Crystal data and structure refinement for P_4-P_2HP**	
Identification code	P2HP-P4
Empirical formula	$C_{36.5}H_{42.36}N_5O_{7.13}$
Formula weight	665.19
Temperature/K	290.0(2)
Crystal system	monoclinic
Space group	$P2_1$
a/Å	16.6077(8)
b/Å	6.3463(3)
c/Å	18.3211(9)
α/°	90
β/°	109.933(5)
γ/°	90
Volume/Å^3	1815.31(16)
Z	2
ρ_{calc}g/cm^3	1.217
μ/mm^{-1}	0.699
F(000)	707.0
Crystal size/mm^3	0.18 × 0.06 × 0.04
Radiation	Cu Kα (λ = 1.54184)
2Θ range for data collection/°	9.896 to 143.886
Index ranges	-20 ≤ h ≤ 20, -7 ≤ k ≤ 7, -22 ≤ l ≤ 19
Reflections collected	13382
Independent reflections	6926 [R_{int} = 0.0244, R_{sigma} = 0.0353]
Data/restraints/parameters	6926/29/481

Goodness-of-fit on F^2	1.039
Final R indexes [I>=2σ (I)]	R_1 = 0.0480, wR_2 = 0.1294
Final R indexes [all data]	R_1 = 0.0604, wR_2 = 0.1398
Largest diff. peak/hole / e Å^{-3}	0.40/-0.20
Flack parameter	-0.01(11)
CCDC No.	2238180

SI 3.1.7 HP_2H SC-XRD:

The crystal structure data was obtained from colourless plank crystals, crystallised via slow evaporation of an ethanol solution of peptide **HP_2H**. The crystal was mounted on a Mitegen micromount in Paratone immersion oil and kept at 295 K using an Oxford Cryosystems 800-series Cryostream. The first hydroxyproline residue was disordered on hydroxyl group and at the Cγ position (C18), likely to due to the presence of *endo* and *exo* ring puckering within the structure. The crystal structure is nonporous containing no significant void space. Both hydrogen bonds present in the **HP_3** and **P_3H** crystal structure are present within the crystal structure between the same atoms, extending along the *b*-axis.

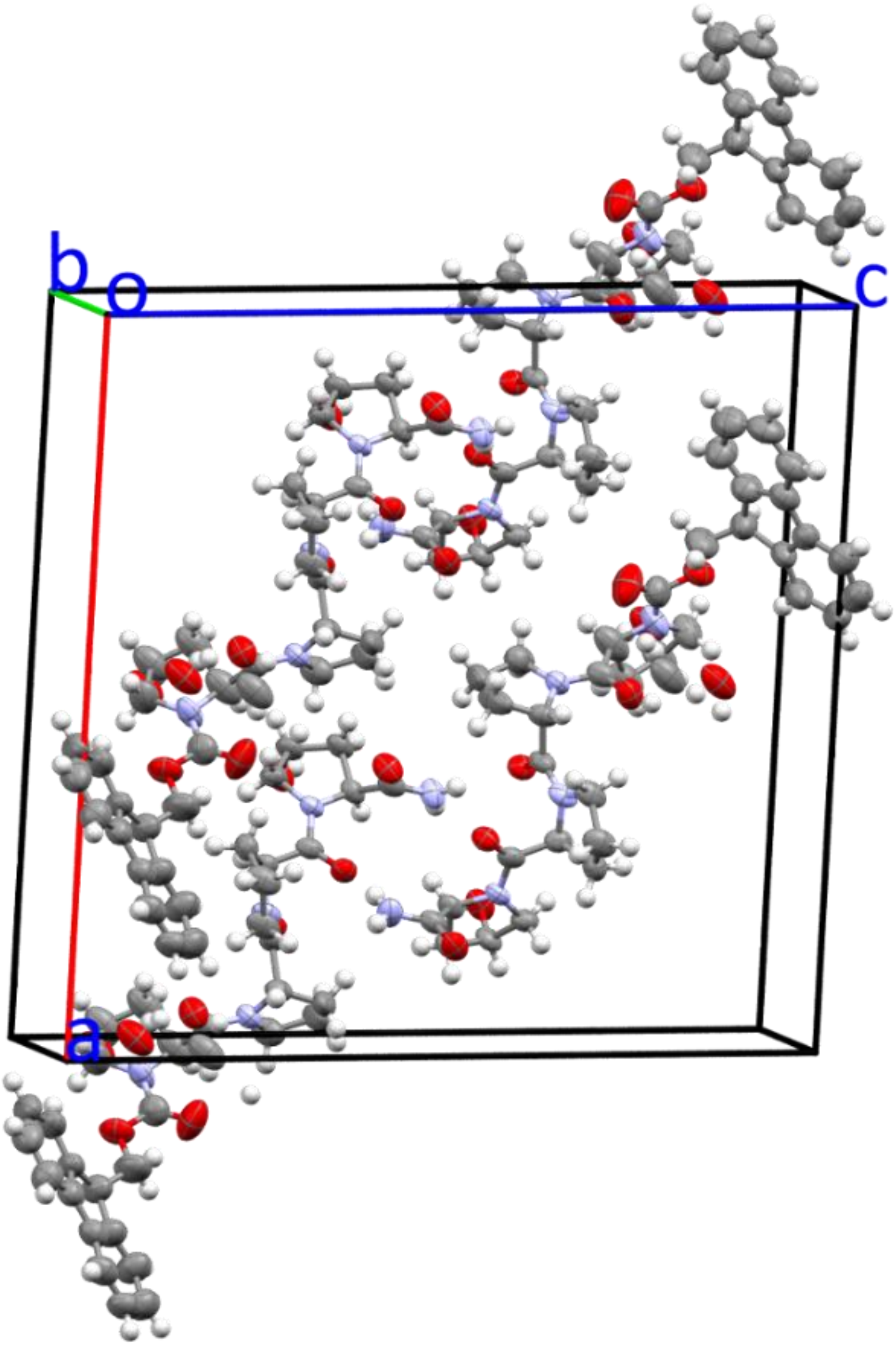

Figure S39 – Crystal structure of peptide **HP_2H** showing the unit cell, atomic displacement parameters are shown at 50 % probability .

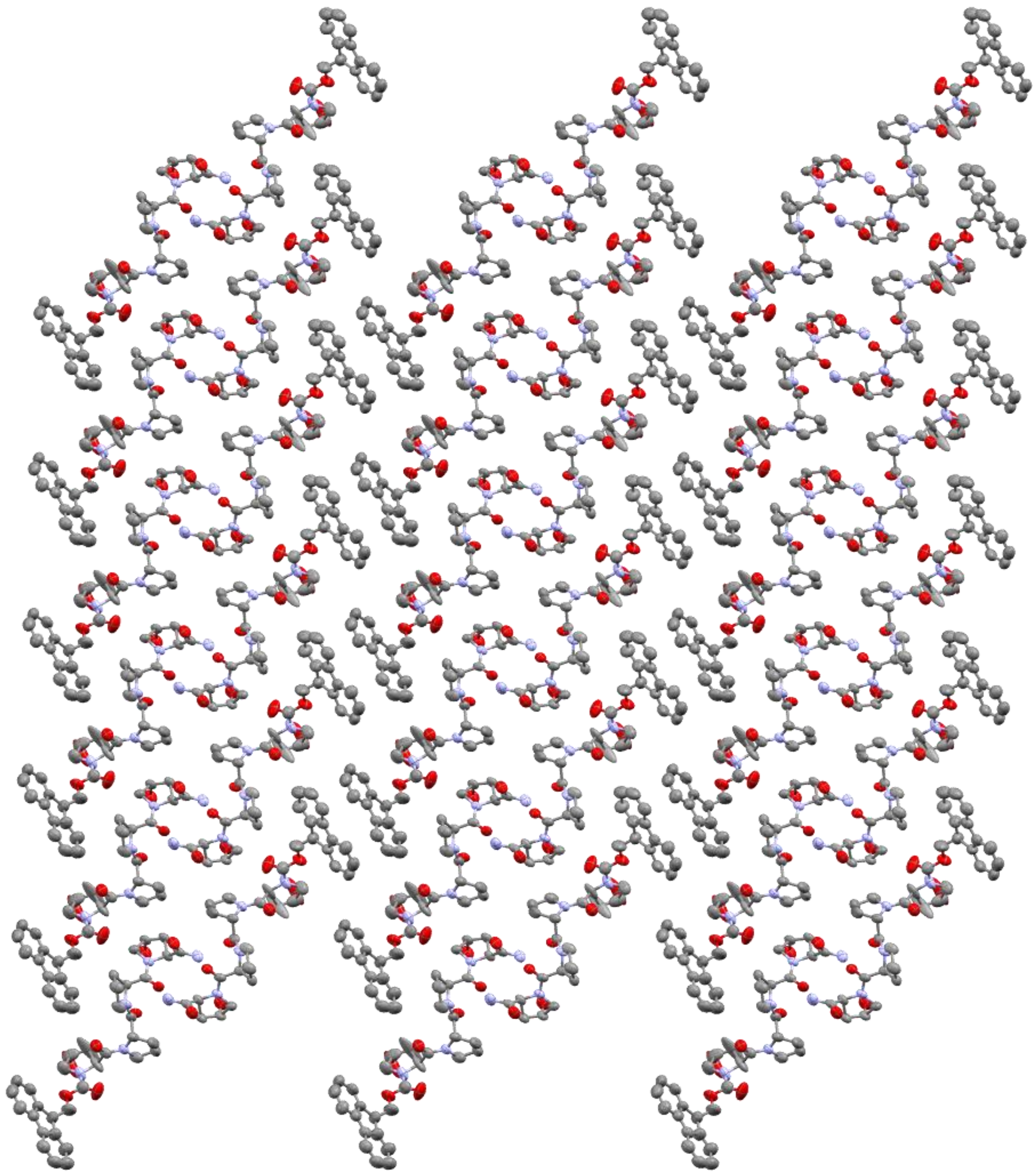

Figure S40 – Crystal structure of peptide **HP$_2$H** showing the packed extended structure (3x3x3), view along the *b* axis, hydrogens not shown, atomic displacement parameters are shown at 50 % probability

Crystal data and structure refinement for HP_2H	
Identification code	HP2H
Empirical formula	$C_{35}H_{40}N_5O_8$
Formula weight	658.72
Temperature/K	295
Crystal system	monoclinic
Space group	C2
a/Å	22.476(2)
b/Å	6.2932(7)
c/Å	23.245(3)
α/°	90
β/°	92.036(10)
γ/°	90
Volume/Å^3	3285.8(6)
Z	4
ρ_{calc}g/cm^3	1.332
μ/mm^{-1}	0.788
F(000)	1396.0
Crystal size/mm^3	0.16 × 0.03 × 0.02
Radiation	Cu Kα (λ = 1.54184)
2Θ range for data collection/°	7.612 to 136.478
Index ranges	-19 ≤ h ≤ 26, -7 ≤ k ≤ 7, -27 ≤ l ≤ 27
Reflections collected	11409
Independent reflections	6003 [R_{int} = 0.1158, R_{sigma} = 0.1952]
Data/restraints/parameters	6003/5/421
Goodness-of-fit on F^2	0.840
Final R indexes [I>=2σ (I)]	R_1 = 0.0835, wR_2 = 0.1224
Final R indexes [all data]	R_1 = 0.1873, wR_2 = 0.1585
Largest diff. peak/hole / e Å^{-3}	0.40/-0.21
Flack parameter	0.7(5)
CCDC No.	2238160

SI 3.1.8 Cis-HP$_2$H SC-XRD:

The crystal structure data was obtained from colourless plank crystals, crystallised via slow evaporation of an ethanol/acetonitrile solution of peptide **Cis-HP$_2$H**. The crystal was mounted on a Mitegen micromount in Paratone immersion oil and kept at 150 K using an Oxford Cryosystems 800-series Cryostream. The crystal structure obtained shows the presence of channels within the structure, filled with disordered solvent (volume 628.9 Å^3, 17.1 % / unit cell, Figure S42) and no suitable model could be obtained for the disordered solvent (EtOH and acetonitrile) within the pores of the structure and so a solvent masking routine was used. The electron density was therefore accounted for using a solvent mask within Olex2,[2] giving a solvent accessible volume of 167 Å^3 and containing 36 electrons asymmetric unit. Screening of multiple crystallites suitable for single crystal analysis gave the same crystal structure. The extended structure of **Cis-HP$_2$H** differs from the other Fmoc peptide frameworks as the Fmoc moieties of adjacent peptides no longer face one another, resulting in staggered rather than linear H-bonded layers of the peptides (Figure 4e). The last hydroxyproline's (C-terminus) hydroxyl group is aligned into the pore space, clearly hydrogen bonding to a solvent molecule, with significant electron density adjacent to this group. Therefore, the only intermolecular hydrogen-bonding between peptides is the typical C-terminal NH_2 amide bonding present in all the structures seen previously. This

highlights how small changes in the placement of functional groups can be used to affect the assembly process, with control over even the flexible pyrrolidine ring endo/exo conformations possible by use of 4S versus 4R functional groups, while the polyproline II helix remains as a rigid ligand for placement of these functional groups.

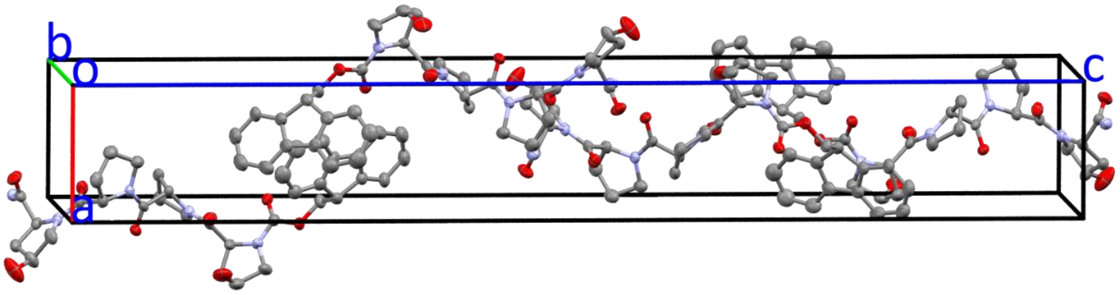

Figure S41 – Crystal structure of peptide ***Cis*-HP$_2$H** showing the unit cell, Atomic displacement parameters are shown at 50 % probability .

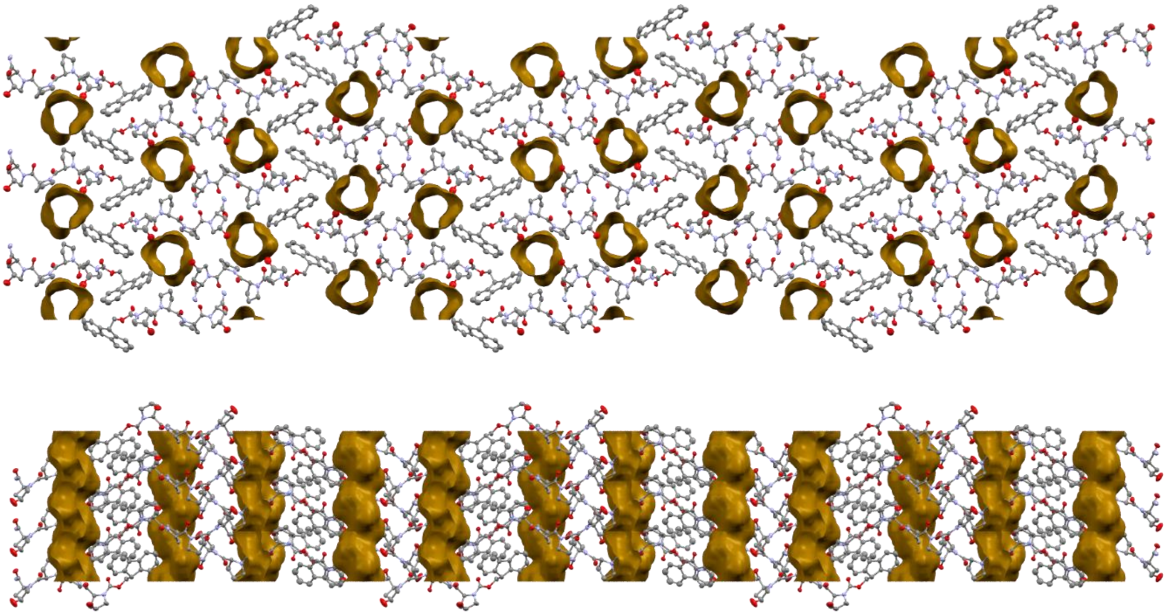

Figure S42 – Crystal structure of peptide ***Cis*-HP$_2$H** showing the packed extended structure (3x3x3), solvent filled channels highlighted in yellow (volume 628.9 Å^3, 17.1 % / unit cell, Probe *r* = 1.2 Å, Grid spacing 0.4 Å). Hydrogens not shown, atomic displacement parameters are shown at 50 % probability . Top: view along *a*-axis, bottom: view along *b*-axis

**Crystal data and structure refinement for *Cis*-HP$_2$H.**	
Identification code	Cis-HP2H
Empirical formula	$C_{35}H_{41}N_5O_8$
Formula weight	659.73
Temperature/K	150.15
Crystal system	orthorhombic
Space group	$P2_12_12_1$

a/Å	6.4160(2)
b/Å	12.0836(6)
c/Å	47.4708(17)
α/°	90
β/°	90
γ/°	90
Volume/Å^3	3680.3(3)
Z	4
ρ_{calc}g/cm^3	1.191
μ/mm^{-1}	0.085
F(000)	1400.0
Crystal size/mm^3	0.26 × 0.06 × 0.05
Radiation	MoKα (λ = 0.71073)
2Θ range for data collection/°	6.578 to 57.246
Index ranges	-6 ≤ h ≤ 8, -16 ≤ k ≤ 15, -50 ≤ l ≤ 63
Reflections collected	18190
Independent reflections	7790 [R_{int} = 0.0457, R_{sigma} = 0.0744]
Data/restraints/parameters	7790/0/440
Goodness-of-fit on F^2	1.020
Final R indexes [I>=2σ (I)]	R_1 = 0.0600, wR_2 = 0.1215
Final R indexes [all data]	R_1 = 0.0926, wR_2 = 0.1348
Largest diff. peak/hole / e Å^{-3}	0.23/-0.25
Flack parameter	0.0(6)
CCDC No.	2238252

SI 3.1.9 AcHP$_2$H SC-XRD:

The crystal structure data was obtained from colourless needle crystals, crystallised via slow evaporation of an acetonitrile solution of peptide **AcHP$_2$H**. The crystals obtained formed packed fibrous assemblies that did not diffract well due to low crystal volumes requiring long exposure times. The crystal was mounted on a Mitegen micromount in Paratone immersion oil and at 294. The crystal structure obtained showed a porous structure (Volume 415.06 Å^3, 15.6 % / unit cell, Probe *r* = 1.2 Å, Grid spacing 0.4 Å), in the orthorhombic $P2_12_12_1$ spacegroup, with channels extending along the *a*-axis containing ordered acetonitrile molecules with no apparent strong interactions between the solvent and the peptide within the structure. The crystal was weakly diffracting and no data above 2 I/sig was observed above 1.1 angstroms. Consequently, the data were truncated at this resolution.

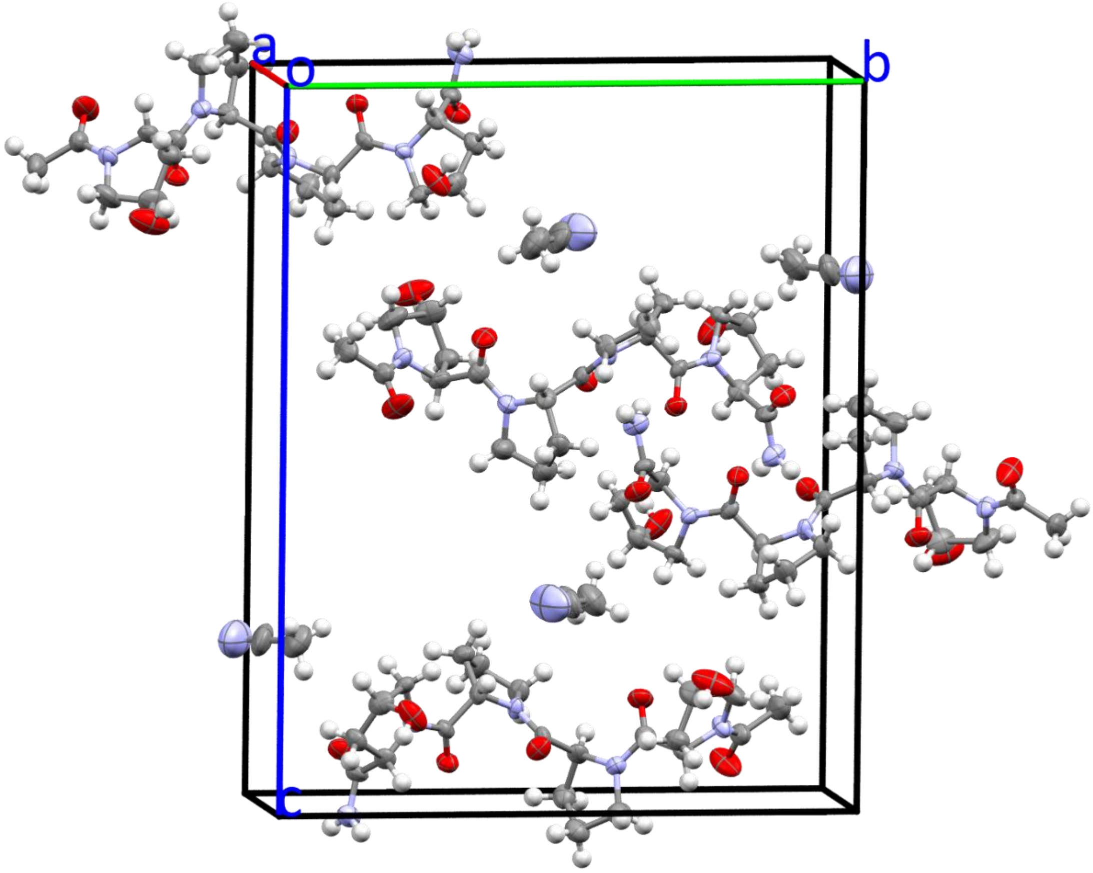

Figure S43 – Crystal structure of peptide **AcHP$_2$H** showing the unit cell, Atomic displacement parameters are shown at 50 % probability .

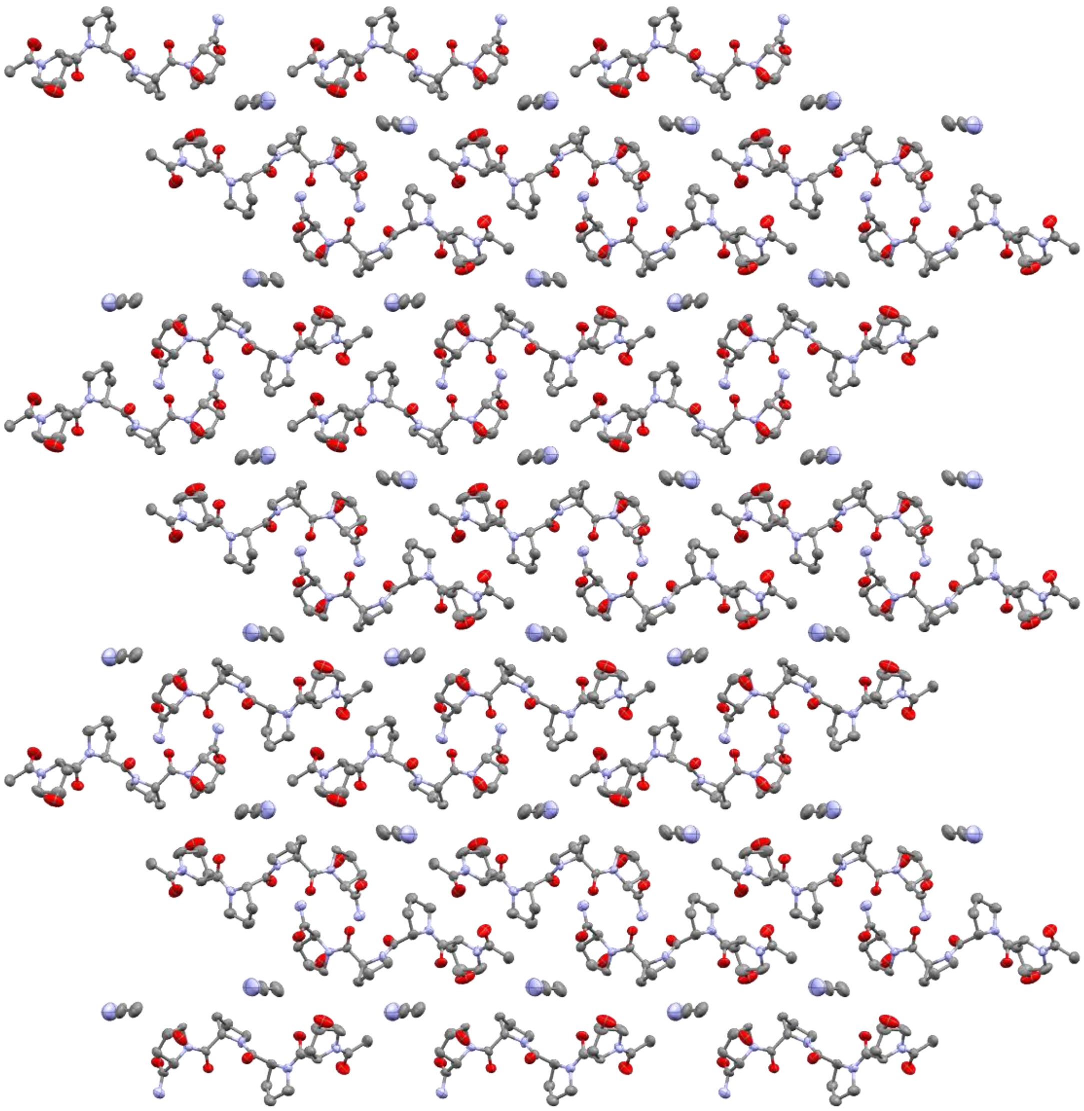

Figure S44 – Crystal structure of peptide **AcHP₂H** showing the packed extended structure (3x3x3), view along the *a* axis, hydrogens not shown, atomic displacement parameters are shown at 50 % probability.

**Crystal data and structure refinement for AcHP$_2$H**	
Identification code	AcHP2H
Empirical formula	$C_{24}H_{36}N_6O_7$
Formula weight	520.59
Temperature/K	294.0(5)
Crystal system	orthorhombic
Space group	$P2_12_12_1$
a/Å	6.3773(10)
b/Å	18.232(4)
c/Å	22.857(5)
α/°	90
β/°	90

γ/°	90
Volume/Å^3	2657.6(9)
Z	4
ρ_{calc}g/cm^3	1.301
μ/mm^{-1}	0.097
F(000)	1112.0
Crystal size/mm^3	0.19 × 0.02 × 0.02
Radiation	Mo Kα (λ = 0.71073)
2Θ range for data collection/°	6.634 to 59.564
Index ranges	-8 ≤ h ≤ 8, -25 ≤ k ≤ 23, -30 ≤ l ≤ 28
Reflections collected	26353
Independent reflections	6766 [R_{int} = 0.2746, R_{sigma} = 0.4001]
Data/restraints/parameters	6766/0/338
Goodness-of-fit on F^2	0.966
Final R indexes [I>=2σ (I)]	R_1 = 0.1033, wR_2 = 0.1305
Final R indexes [all data]	R_1 = 0.3459, wR_2 = 0.1936
Largest diff. peak/hole / e Å^{-3}	0.18/-0.23
Flack parameter	-1.7(10)
CCDC No.	2234312

SI 3.1.10 AcP$_4$ SC-XRD:

The crystal structure data was obtained from colourless crystalline needles, crystallised via vapour diffusion of Et_2O into a solution of **AcP$_4$** in $CHCl_3$. The crystal was mounted on a Mitegen micromount in Paratone immersion oil and kept at 150 K using an Oxford Cryosystems 800-series Cryostream. The crystal structure obtained showed a porous structure (Volume 572.37 Å^3, 36.1 % / unit cell, Probe *r* = 1.2 Å, Grid spacing 0.4 Å), with in the monoclinic P2$_1$ spacegroup, with channels extending in a 2D layer along the *b*- and *a*-axis containing ordered chloroform molecules, with two solvent molecules per asymmetric unit, hydrogen bonding to the Pro1 and Pro4 carbonyl groups.

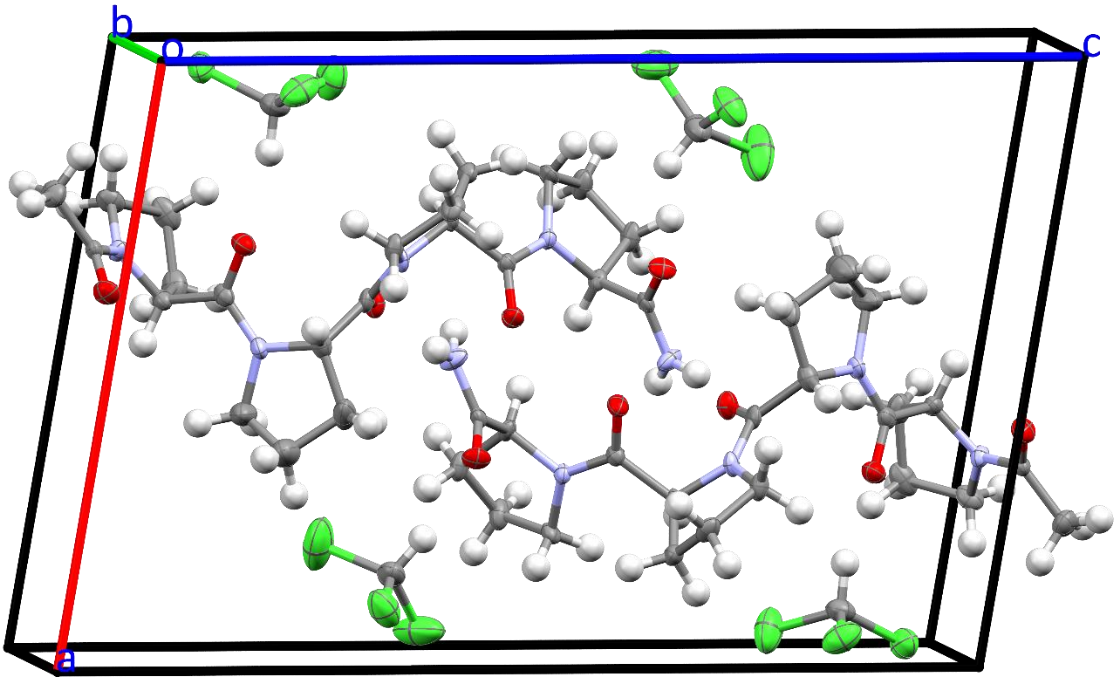

Figure S45 – Crystal structure of peptide **AcP$_4$** showing the unit cell, Atomic displacement parameters are shown at 50 % probability .

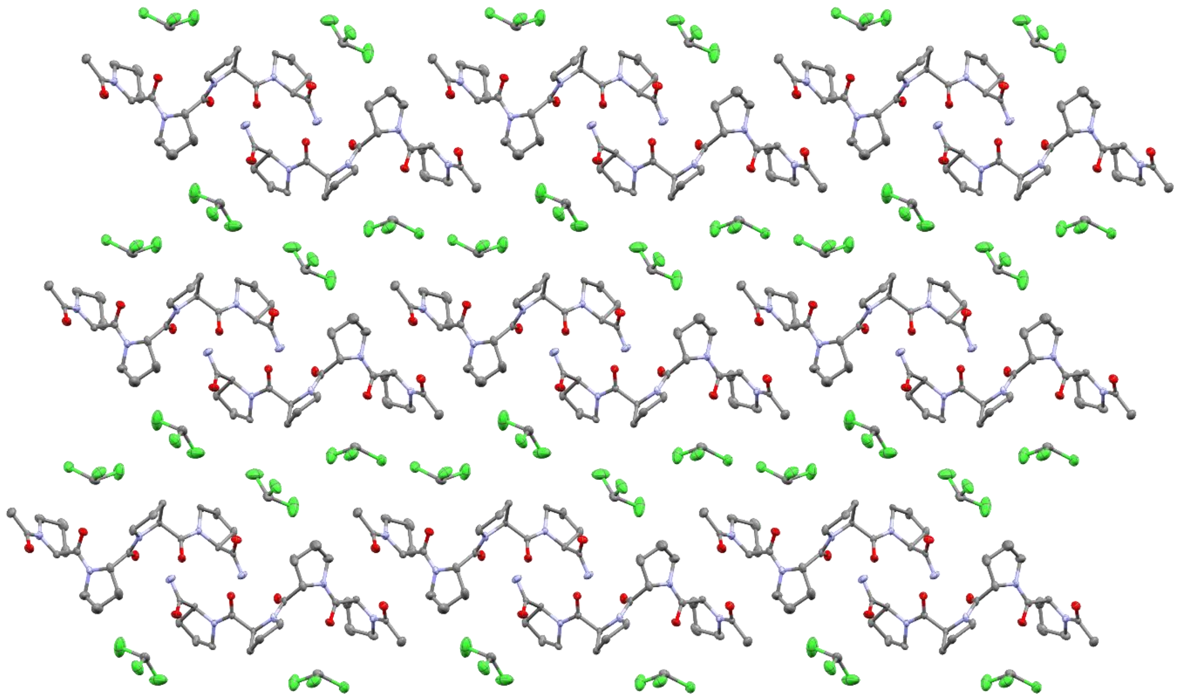

Figure S46 – Crystal structure of peptide **AcP$_4$** showing the packed extended structure (3x3x3), view along the *b* axis, hydrogens not shown, atomic displacement parameters are shown at 50 % probability.

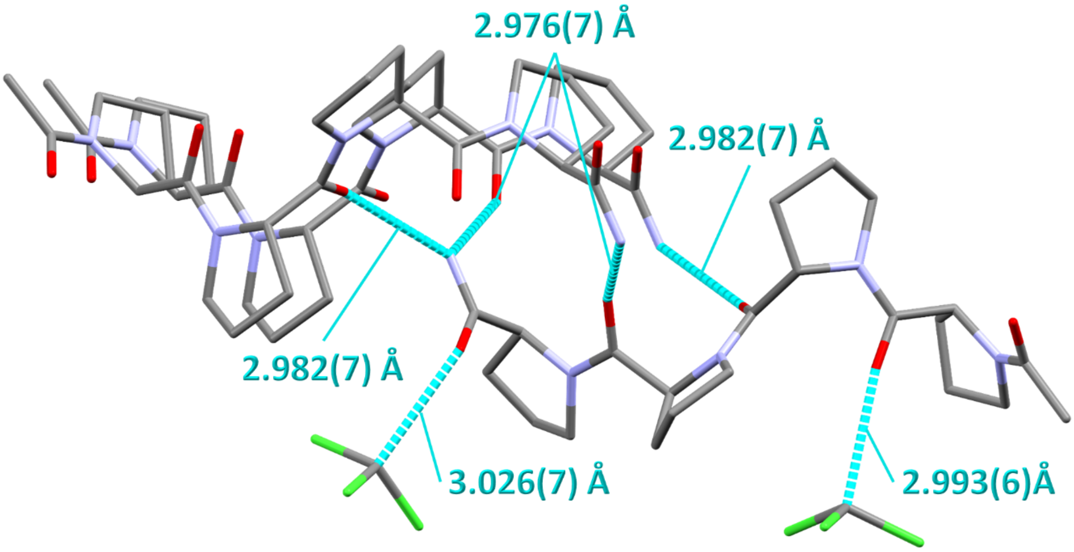

Figure S47 – Crystal structure of peptide **AcP$_4$** showing hydrogen bonding interactions of a single peptide unit, hydrogens not shown, modelled as capped sticks.

**Crystal data and structure refinement for AcP$_4$**	
Identification code	AcP4
Empirical formula	$C_{24}H_{35}Cl_6N_5O_5$
Formula weight	686.27
Temperature/K	150.0(7)
Crystal system	monoclinic
Space group	$P2_1$
a/Å	13.0167(8)
b/Å	6.2940(6)
c/Å	19.6114(12)
α/°	90
β/°	99.377(6)
γ/°	90
Volume/Å^3	1585.2(2)
Z	2
ρcalcg/cm^3	1.438
μ/mm^{-1}	0.584
F(000)	712.0
Crystal size/mm^3	0.51 × 0.06 × 0.04
Radiation	Mo Kα (λ = 0.71073)
2Θ range for data collection/°	6.59 to 50.038
Index ranges	-15 ≤ h ≤ 15, -7 ≤ k ≤ 7, -22 ≤ l ≤ 23
Reflections collected	14583
Independent reflections	5615 [R_{int} = 0.0679, R_{sigma} = 0.0900]
Data/restraints/parameters	5615/1/370
Goodness-of-fit on F^2	1.017
Final R indexes [I>=2σ (I)]	R_1 = 0.0512, wR_2 = 0.0969
Final R indexes [all data]	R_1 = 0.0783, wR_2 = 0.1084
Largest diff. peak/hole / e Å^{-3}	0.46/-0.38
Flack parameter	-0.09(5)
CCDC No.	2264145

SI 3.2 Powder XRD data:

SI 3.2.1 P_4 PD-XRD:

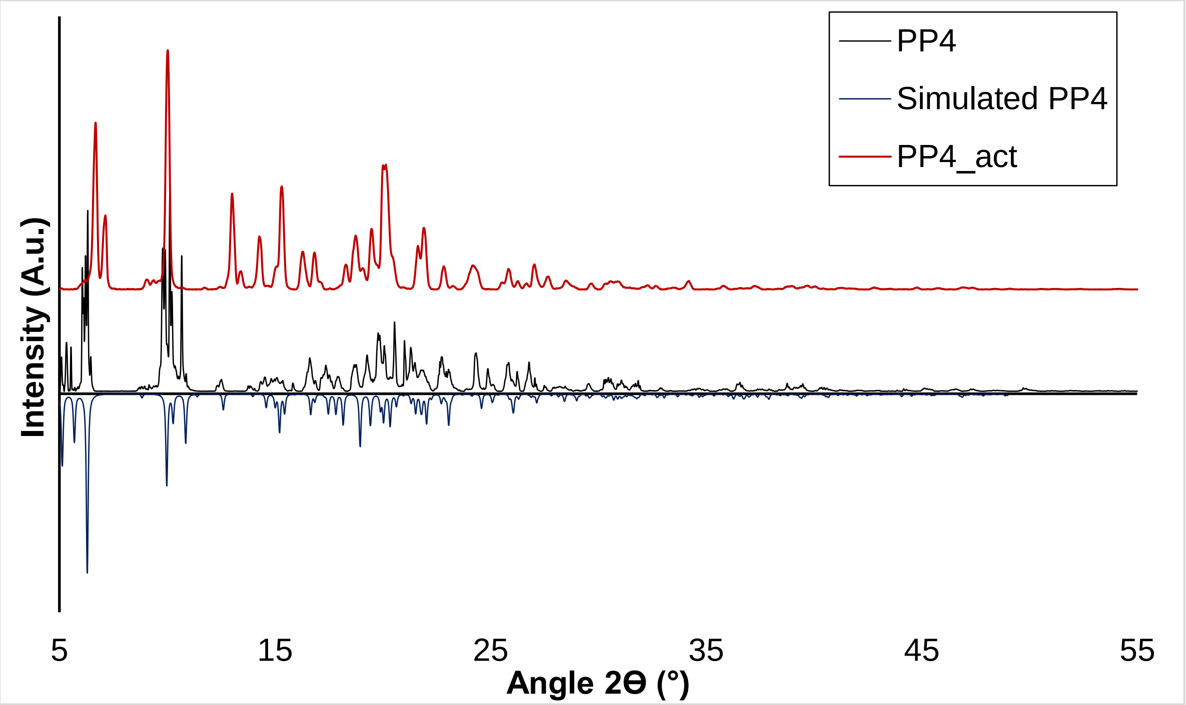

Figure S48 – PD-XRD of $\mathbf{P_4}$ simulated from SC-XRD data (**black**), $\mathbf{P_4}$ (unground) experimental PD-XRD (**blue,** bottom) $\mathbf{P_4}$ after activation at 45 °C under high vacuum (**red,** top)[1]

SI 3.2.2 HP_3 PD-XRD:

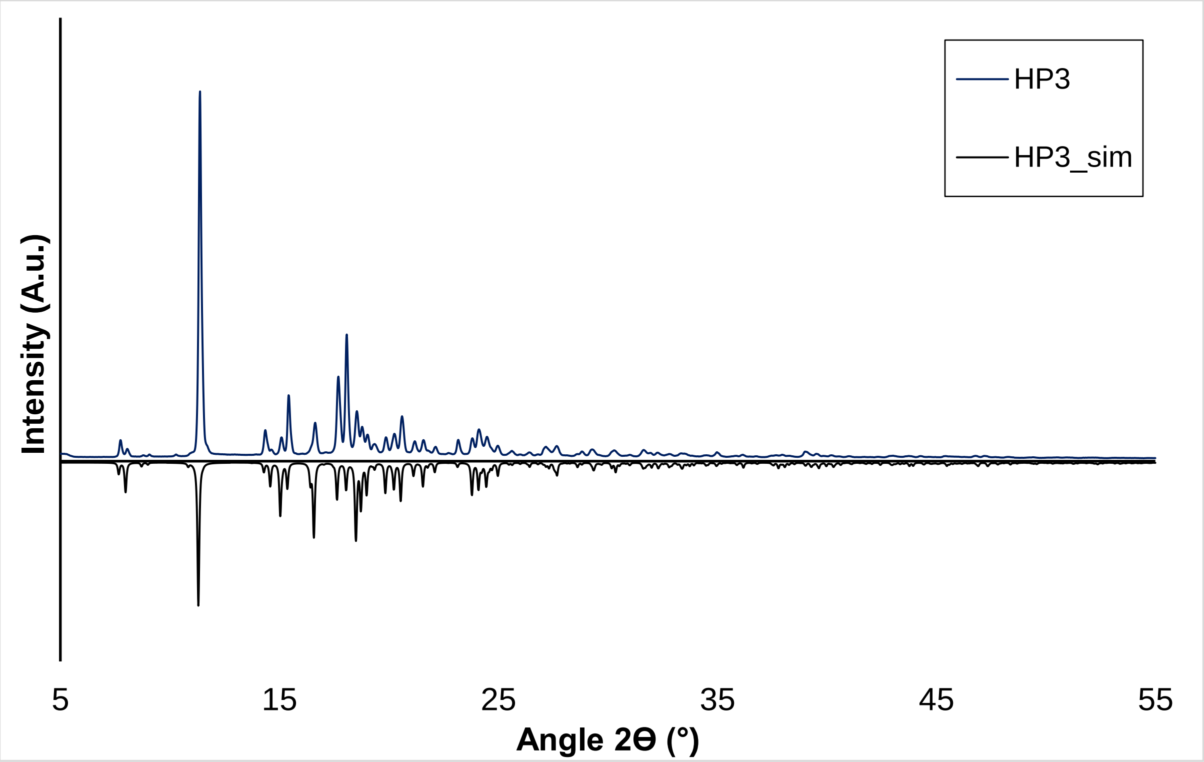

Figure S49 – PD-XRD of $\mathbf{HP_3}$ simulated from SC-XRD data (**black,** bottom), $\mathbf{HP_3}$ experimental PD-XRD (**blue,** top)

SI 3.2.3 PHP_2 PD-XRD:

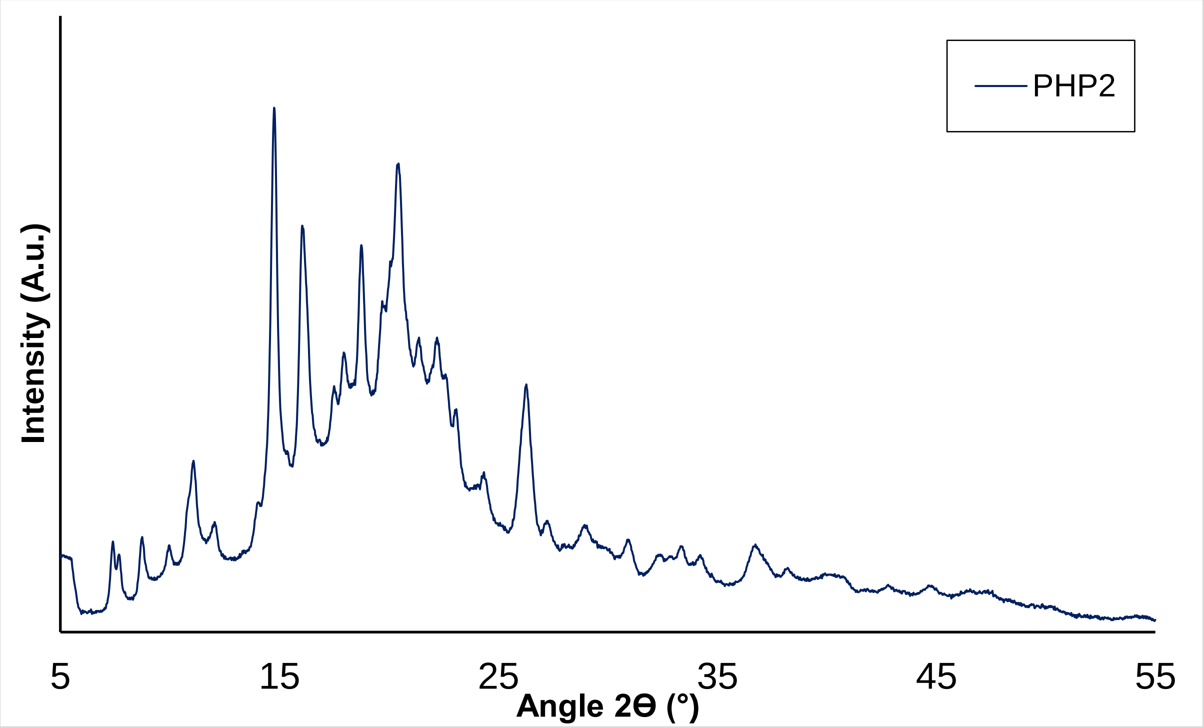

Figure S50 – PHP_2 experimental PD-XRD, sample was poorly crystalline and contained amorphous peptide producing a broad background peak.

SI 3.2.4 P_2HP PD-XRD:

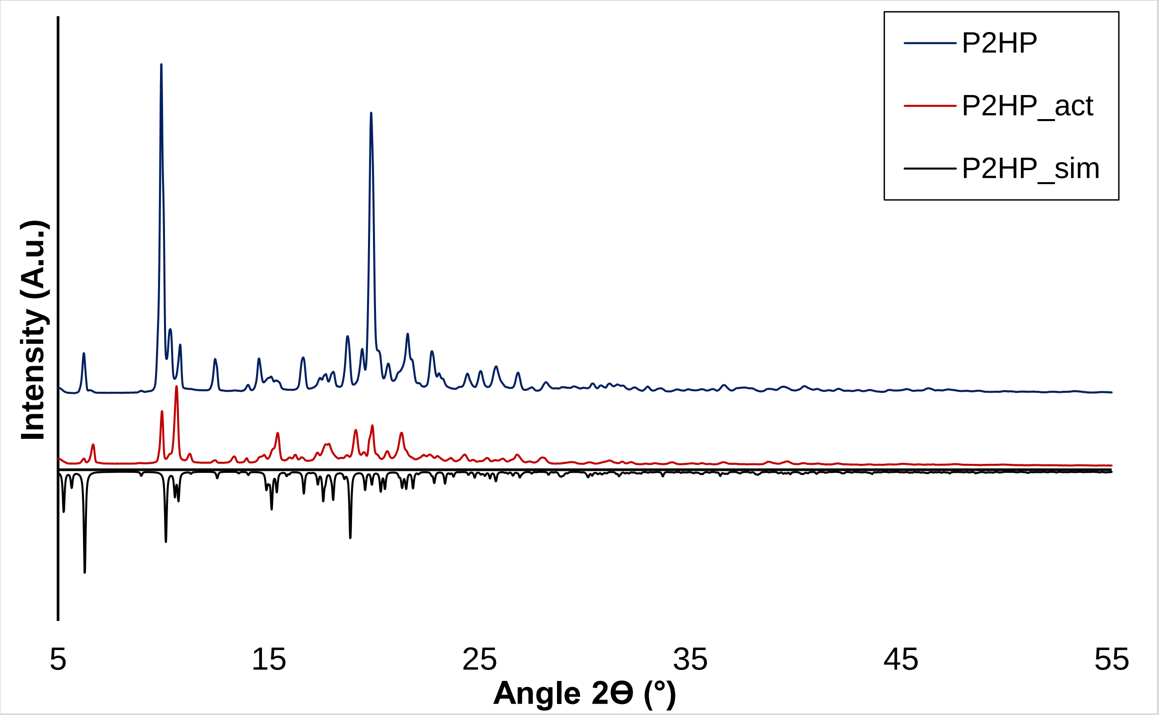

Figure S51 – PD-XRD of **P_2HP** simulated from SC-XRD data (**black, bottom**), **P_2HP** experimental PD-XRD (**blue, top**), **P_2HP** after activation at 45 °C under high vacuum (**red**)

SI 3.2.5 P_3H PD-XRD:

Peptide **P_3H** did not crystallise well as once precipitated from solution an insoluble white solid formed preventing redissolution, as such a suitable powder pattern could not be obtained, despite the crystallisation of some single crystals suitable for SCXRD, sufficient crystalline material was not obtained for PDXRD analysis.

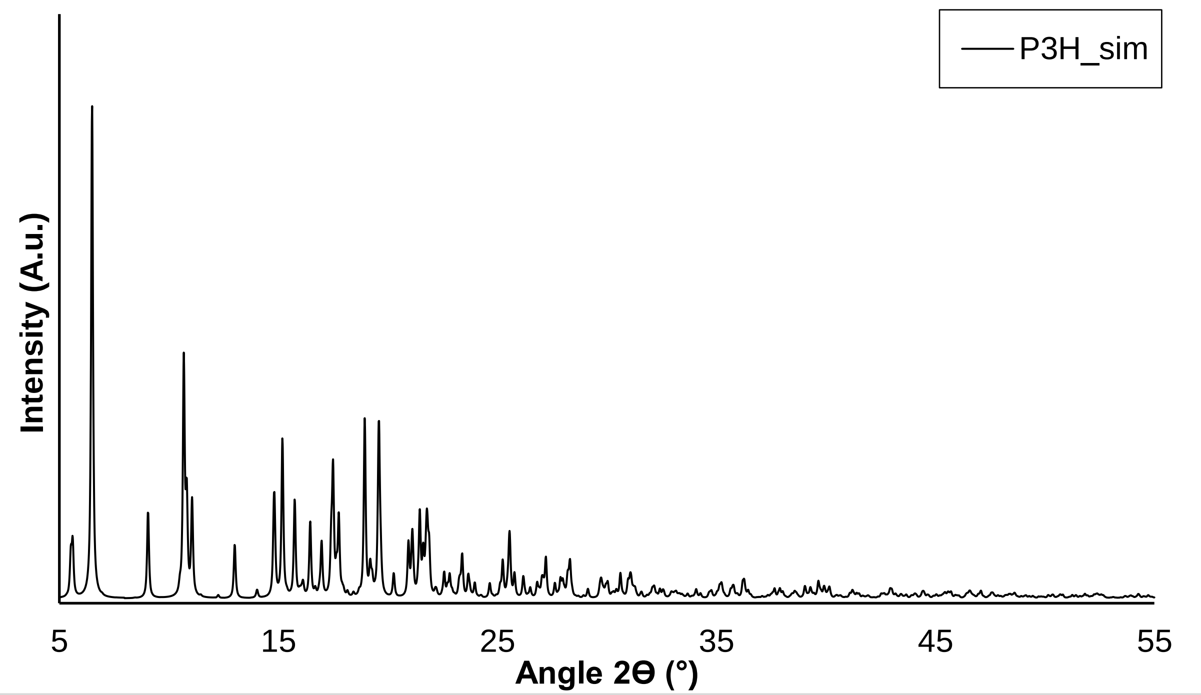

Figure S52 – PD-XRD of **P_3H** simulated from SC-XRD data

SI 3.2.6 P_4+P_2HP PD-XRD:

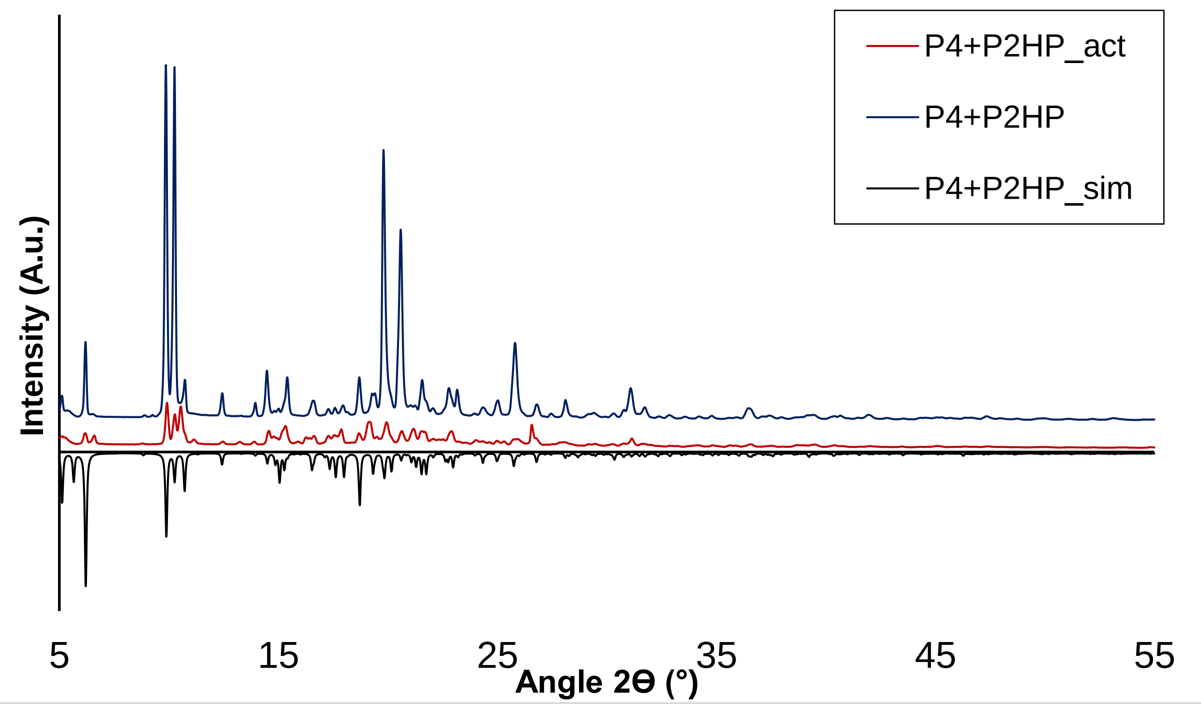

Figure S53 – PD-XRD of **P_4+ P_2HP** simulated from SC-XRD data (**black, bottom**), **P_4+ P_2HP** experimental PD-XRD (**blue, top**), **P_4+ P_2HP** after activation at 45 °C under high vacuum (**red**)

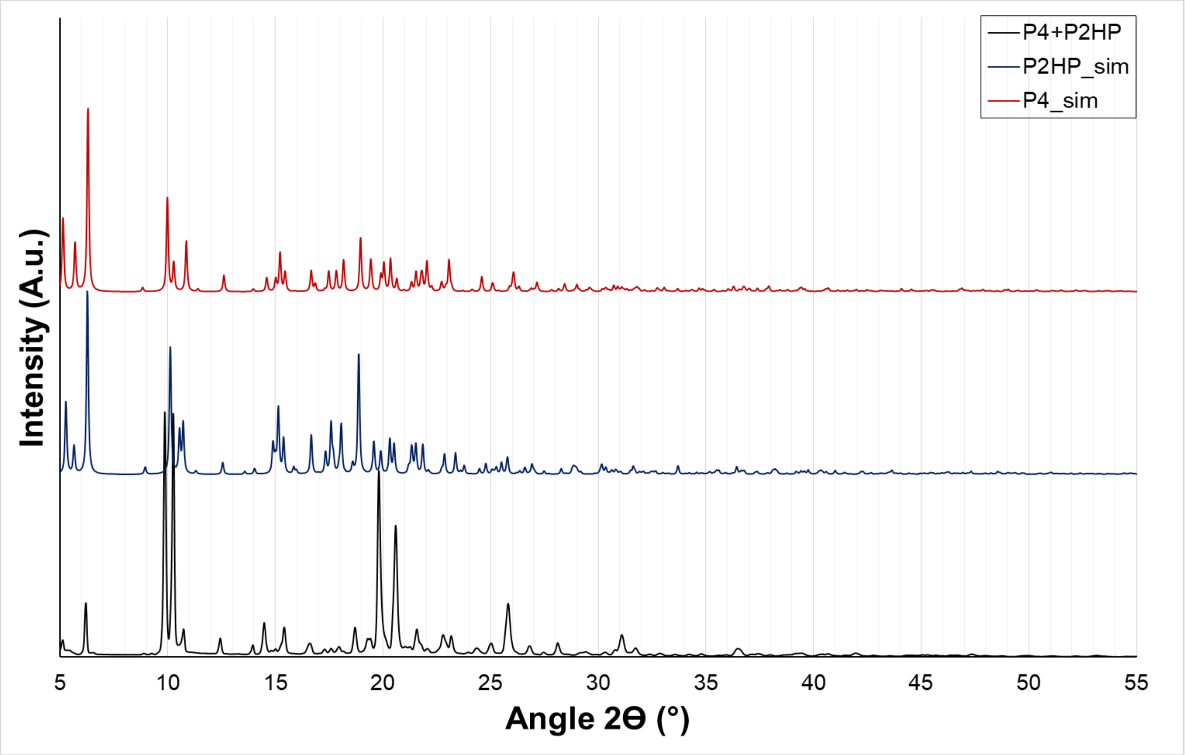

Figure S54 – Comparative powder diffraction pattern showing; **P_4+ P_2HP** experimental PD-XRD (**black, bottom**), **P_2HP** powder pattern simulated from SC-XRD data (**blue**), **P_4** powder pattern simulated from SC-XRD data (**red, top**).

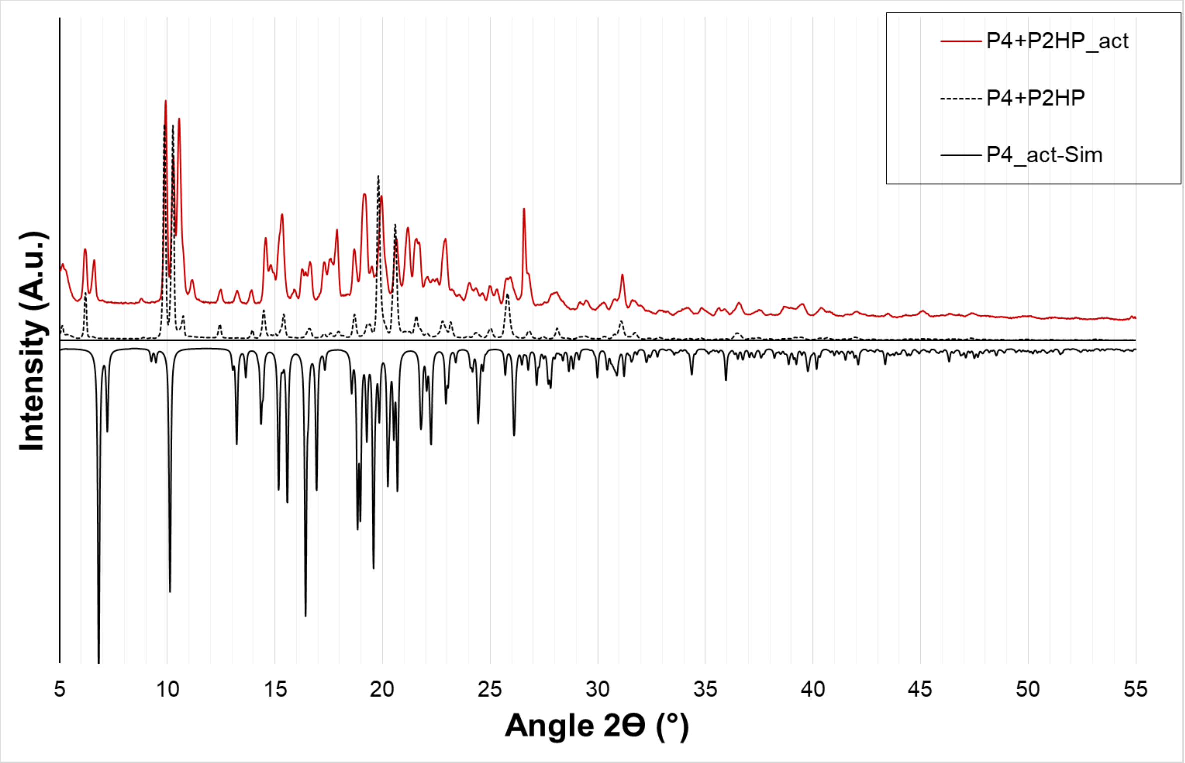

Figure S55 – Comparative powder diffraction pattern showing; **P_4+ P_2HP** experimental PD-XRD (**dotted**), **P_4+ P_2HP** experimental PD-XRD after activation at 45 °C under vacuum (**red**, top), **P_4** activated powder pattern simulated from SC-XRD data (**black, bottom**).[1] Highlights the partial activation and change in phase of the mixed peptide framework.

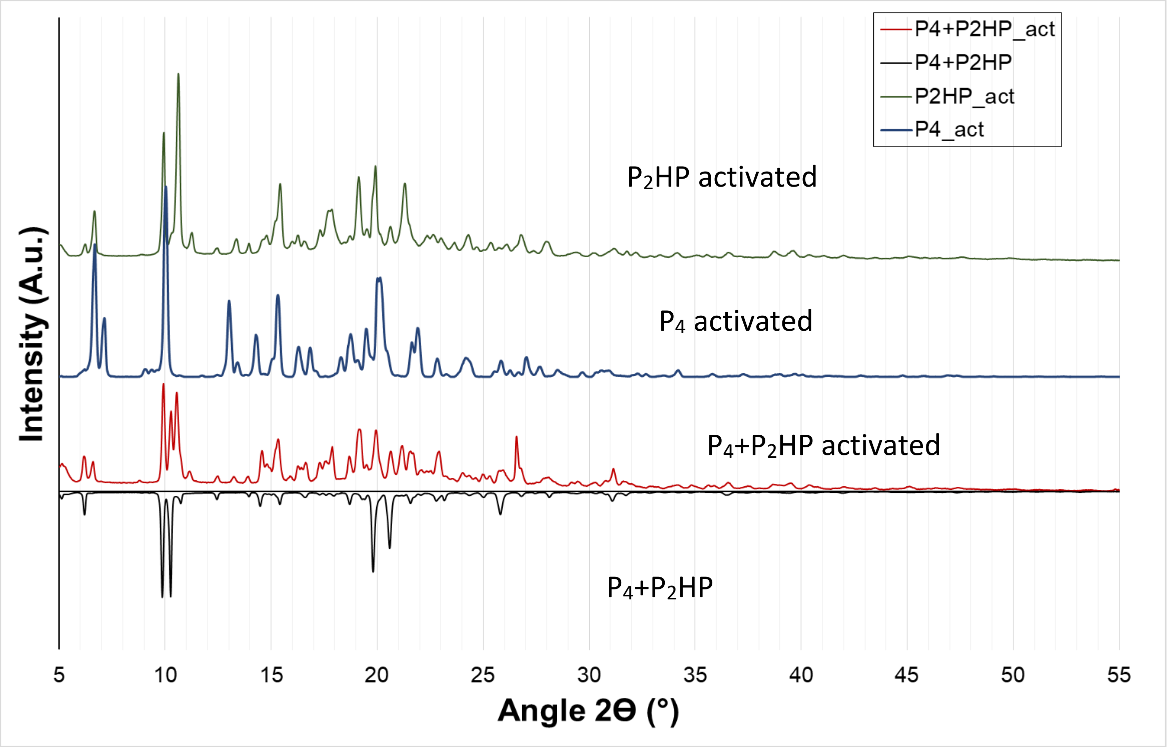

Figure S56 – Comparative powder diffraction pattern showing; **P_4+ P_2HP** experimental PD-XRD (**black, bottom**), **P_4+ P_2HP** experimental PD-XRD after activation at 45 °C under vacuum (**red**), **P_4** experimental PD-XRD after activation at 45 °C under vacuum (**blue**),[1] **P_2HP** experimental PD-XRD after activation at 45 °C under vacuum (**green**). Highlights the partial activation, and subsequent change in phase, of the mixed framework compared to **P_4/ P_2HP** activation, showing similarities to both pure frameworks.

SI 3.2.7 HP_2H PD-XRD:

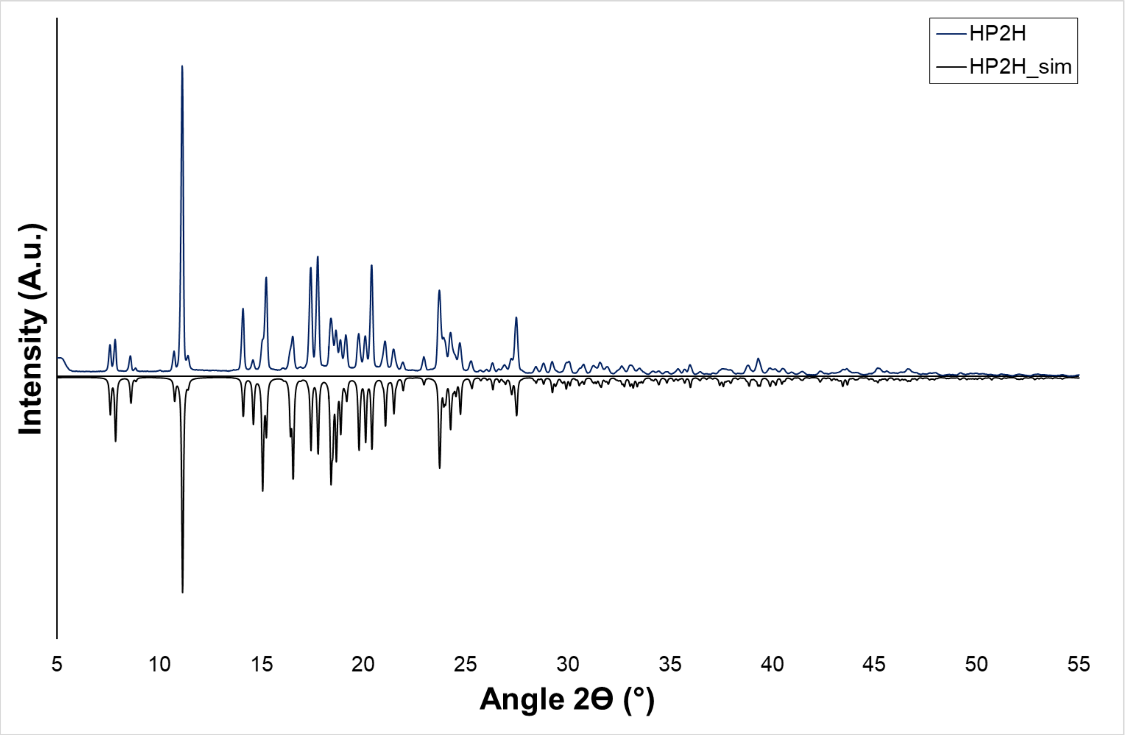

Figure S57 – PD-XRD of **HP_2H** simulated from SC-XRD data (**black, bottom**), **HP_2H** experimental PD-XRD (**blue, top**)

SI 3.2.8 Cis-HP$_2$H PD-XRD:

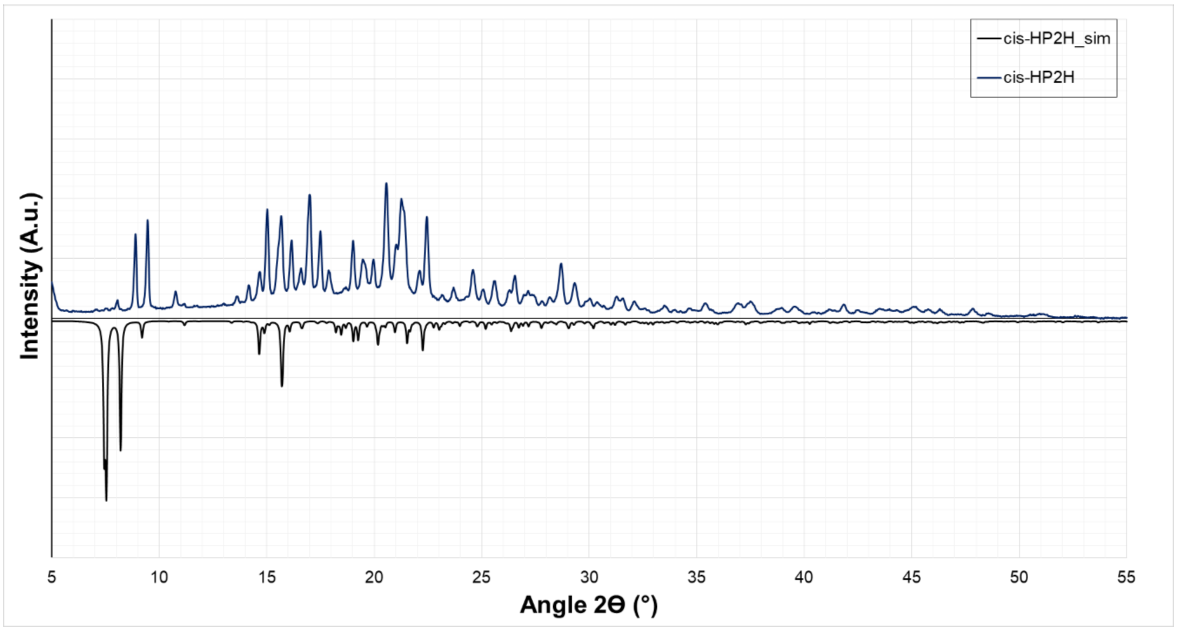

Figure S58 – PD-XRD of ***Cis*-HP$_2$H** simulated from SC-XRD data (**black, bottom**), ***Cis*-HP$_2$H** experimental PD-XRD (**blue, top**)

The experimental spectra for ***Cis*-HP$_2$H** differs significantly from the simulated powder pattern (*Figure S58*), from the crystal structure obtained, suggesting the bulk material differs from the singly crystalline material. Screening of crystals for alternate crystal structures was unsuccessful, all crystals suitable for single crystal analysis exhibited the previously obtained structure.

SI 3.2.9 $AcHP_2H$ PD-XRD:

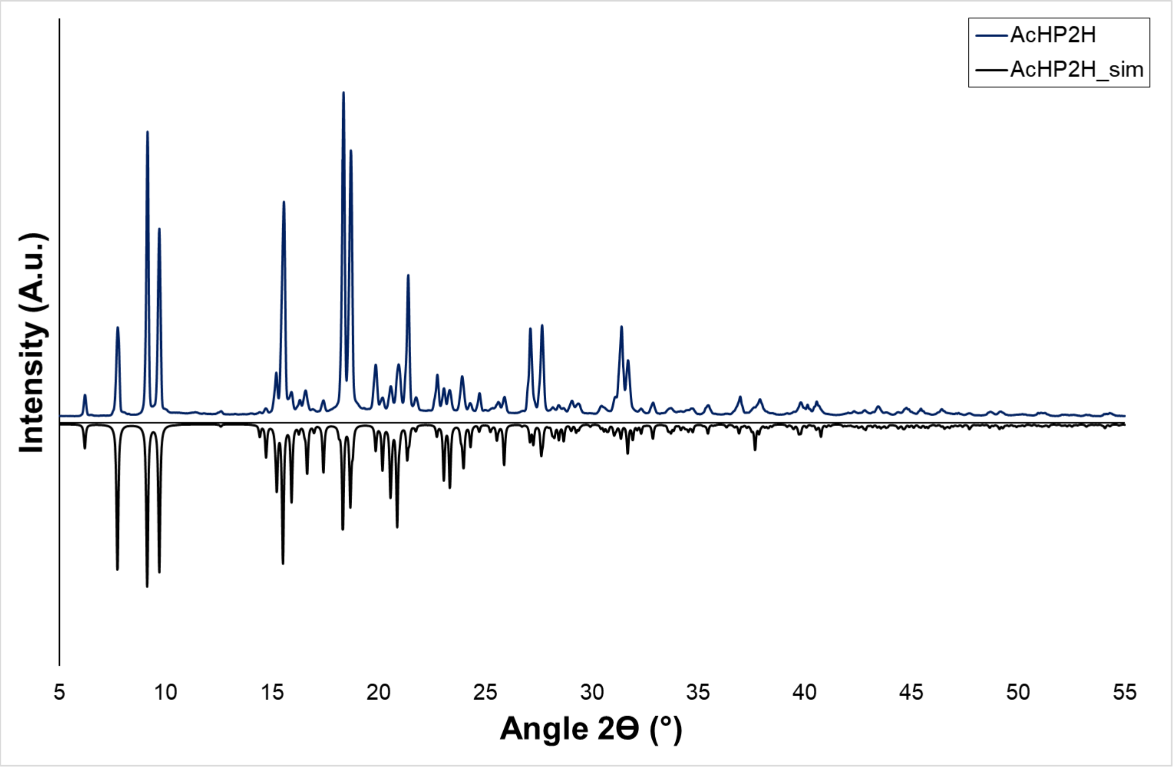

Figure S59 – PD-XRD of **$AcHP_2H$** simulated from SC-XRD data (**black, bottom**), **$AcHP_2H$** experimental PD-XRD (**blue, top**)

SI 3.2.10 AcP_4 PD-XRD:

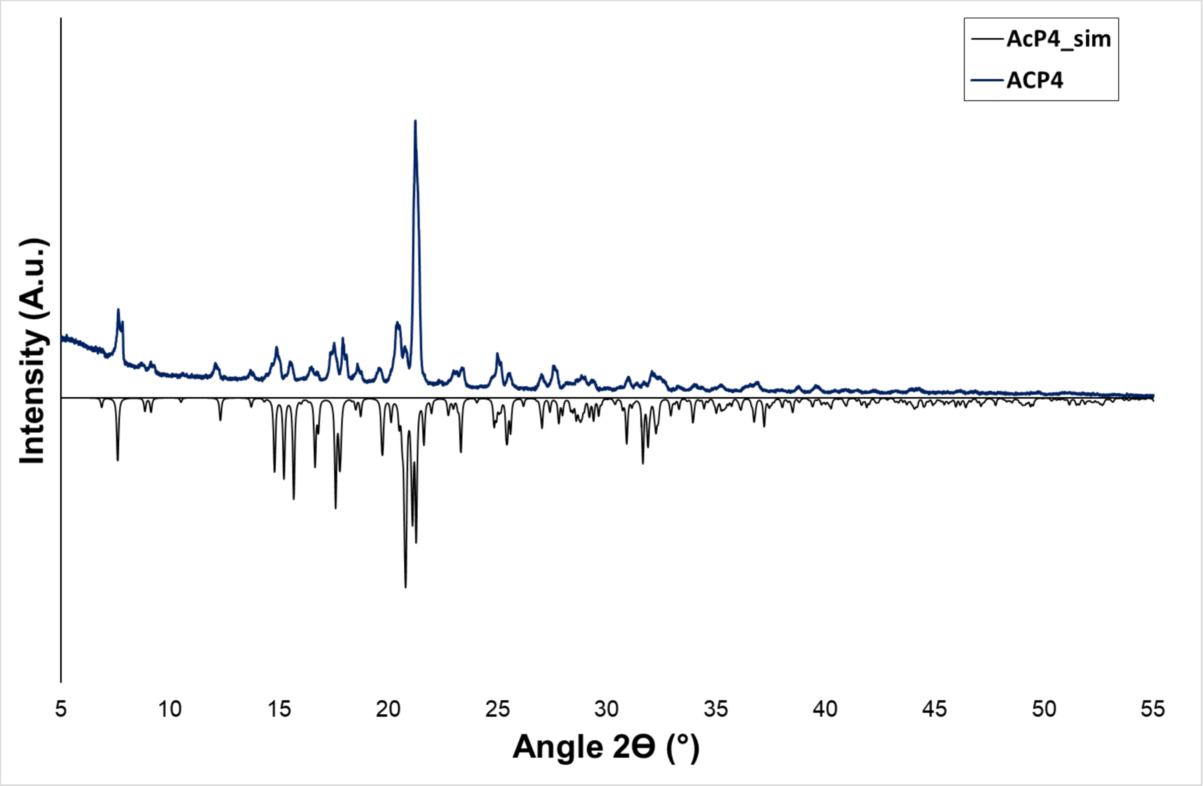

Figure S60 – PD-XRD of **AcP_4** simulated from SC-XRD data (**black, bottom**), **AcP_4** experimental PD-XRD (**blue, top**)

References

(1) Brightwell, D. F.; Truccolo, G.; Samanta, K.; Fenn, E. J.; Holder, S. J.; Shepherd, H. J.; Hawes, C. S.; Palma, A. A Reversibly Porous Supramolecular Peptide Framework. *Chem. – A Eur. J.* **2022**, *28* (66), e202202368. https://doi.org/10.1002/CHEM.202202368.

(2) Dolomanov, O. V.; Bourhis, L. J.; Gildea, R. J.; Howard, J. A. K.; Puschmann, H. OLEX2: A Complete Structure Solution, Refinement and Analysis Program. *J. Appl. Crystallogr.* **2009**, *42* (2), 339–341. https://doi.org/10.1107/S0021889808042726.

(3) Sheldrick, G. M. A Short History of SHELX. *Acta Crystallographica Section A: Foundations of Crystallography*. 2008, pp 112–122. https://doi.org/10.1107/S0108767307043930.

(4) Sheldrick, G. M. Crystal Structure Refinement with SHELXL. *Acta Crystallogr. Sect. C Struct. Chem.* **2014**, *71* (1), 3–8. https://doi.org/10.1107/S2053229614024218.

(5) Sheldrick, G. M. SHELXT – Integrated Space-Group and Crystal-Structure Determination. *Acta Crystallogr. Sect. A Found. Adv.* **2015**, *71* (1), 3–8. https://doi.org/10.1107/S2053273314026370.
